# The Area of Secondary Hyperalgesia following Heat Stimulation in Healthy Male Volunteers: Inter- and Intra-Individual Variance and Reproducibility

**DOI:** 10.1371/journal.pone.0155284

**Published:** 2016-05-11

**Authors:** Morten Sejer Hansen, Jørn Wetterslev, Christian Bressen Pipper, Rebecca Østervig, Mohammad Sohail Asghar, Jørgen Berg Dahl

**Affiliations:** 1 Department of Anesthesiology 4231, Centre of Head and Orthopedics, Rigshospitalet, Copenhagen, Denmark; 2 Department 7812, Copenhagen Trial Unit, Centre for Clinical Intervention Research, Copenhagen, Denmark; 3 Section of Biostatistics, Faculty of Health, Copenhagen University, Copenhagen, Denmark; 4 Department of Anesthesiology 4231, Centre of Head and Orthopedics, Rigshospitalet, Copenhagen, Denmark; 5 Department of Anesthesiology, Bispebjerg and Frederiksberg Hospitals, Copenhagen, Denmark; Nagoya University Graduate School of Medicine, JAPAN

## Abstract

**Introduction:**

Clinical pain models can be applied when investigating basic physiologic pain responses in healthy volunteers. Several pain models exist; however, only few have been adequately validated. Our primary aim with this prospective study was to investigate the intra- and inter-individual variation in secondary hyperalgesia elicited by brief thermal sensitization (45°C for 3 min) in healthy volunteers.

**Material and Methods:**

Fifty healthy volunteers were included. Areas of secondary hyperalgesia following brief thermal sensitization were investigated by 2 observers on 4 experimental days, with a minimum interval of 7 days. Additionally, heat pain detection threshold and pain during thermal stimulation (45°C for 1 min.), and the psychological tests Pain Catastrophizing Scale and Hospital Anxiety and Depression Score were applied.

**Results:**

For areas of secondary hyperalgesia, an intra-observer intra-person correlation of 0.85, 95% CI [0.78, 0.90], an intra-observer inter-person correlation of 0.03, 95% CI [0.00, 0.16], and a coefficient of variation of 0.17, 95% CI [0.14, 0.21] was demonstrated. Four percent of the study population had areas of secondary hyperalgesia both below the 1^st^ and above the 3^rd^ quartile considering all included participants. Heat pain detection threshold predicted area of secondary hyperalgesia with an adjusted R^2^ of 0.20 (P = 0.0006).

**Conclusions:**

We have demonstrated a low intra-individual, and a high inter-individual variation in thermally induced secondary hyperalgesia. We conclude that brief thermal sensitization produce secondary hyperalgesia with a high level of reproducibility, which can be applied to investigate different phenotypes related to secondary hyperalgesia in healthy volunteers.

**Trial Registration:**

clinicaltrials.gov NCT02166164

## Introduction

Clinical pain models are important in order to investigate basic physiologic pain responses in both healthy volunteers and patients. Such models play an important role in translational studies and are necessary to bridge the gap between animal and human pain research.

There are numerous pain models, employed to investigate different aspects of the human physiologic pain response [1, 2]. Models applying nociceptive stimulation to the skin, by heat [3–8], cold [9] or electrical stimuli [10] can be used in investigation of injury-induced sensitization of the central nervous system. Central sensitization is believed to be an important factor in the development and maintenance of pain, and represents an uncoupling of the nociceptive stimulus and the nociceptive response [11]. Likewise, central sensitization may have a prominent role in the inter-individual differences in pain sensitivity; the concept that different individuals experience different levels of pain when exposed to identical noxious or nociceptive stimuli.

A standardized heat injury of the skin results in primary hyperalgesia at the site of injury, and secondary hyperalgesia surrounding the traumatized area [1, 3–8]. Injury-induced secondary hyperalgesia is characterized by reduced thresholds for mechanical stimulation, and is supposed to result from an altered central processing of mechano- and nociceptive input in A-fibers from the periphery, so that activation of these fibers produce painful sensations [11–14].

Moreover, a significant inter-individual difference in the size of the area of secondary hyperalgesia may persist, implying that the development of secondary hyperalgesia may be a phenotypic expression [15]. The inter-individual differences in areas of secondary hyperalgesia may be due to genetic [16], physiologic, and psychological differences [17], as well as differences in brain activation during pain stimulation [18]; however, further studies are needed to confirm this hypothesis.

Several pain models investigating cutaneous sensitization exist—each investigating different aspects of cutaneous sensitization. Brief thermal sensitization (BTS) [4–8] induces short lasting cutaneous sensitization, ideal for multiple inductions throughout a study day. With the BTS-model the skin is heated to 45°C for 3 min., resulting in mild pain perception, and short lasting secondary hyperalgesia [4–8]. Thus, the BTS-model can be applied in investigation of central sensitization. To our knowledge there have been no prospective trials investigating intra-individual, inter-individual, and inter-investigator variances of areas of secondary hyperalgesia following BTS. Validation of the models is paramount, and methodological sound studies investigating the inter-and intra-individual reproducibility, as well as the inter- and intra-investigator reproducibility are needed in order to validate the use of the models in future scientific research [19, 20].

The primary aim of this prospective cohort study was to investigate the intra-individual and inter-individual variance in secondary hyperalgesia elicited by brief thermal sensitization in healthy male volunteers.

## Material and Methods

The study was registered at clinicaltrials.gov (NCT02166164), and approved by the Danish Regional Committee on Health Research Ethics (Identifier: H-4-2014-027), and the Danish Data Protection Agency (Identifier: 30–1217). Informed written consent was obtained from all participants before inclusion in the study. The study was conducted at the Department of Anaesthesiology, 4231, Rigshospitalet, Copenhagen, Denmark, in the period from June 10, 2014 to September 17, 2014.

### Study design

This prospective cohort study was designed to evaluate the method of BTS, and consisted of four identical experimental days and one information/inclusion day. To prevent carry-over effects, the information day and each of the four experimental days were separated by a minimum of seven days.

The participants were tested with three procedures, brief thermal sensitization (BTS), heat pain detection threshold (HPDT), and pain during 1 min. thermal stimulation (p-TS) (for definitions, see below) on the four separate experimental days in a predefined sequence (see Randomization and allocation concealment).

All the pain models were performed with the computer-controlled Somedic Senselab MSA Thermotester^™^; size 2.5x5 cm.

On the information day the participants were given the psychological tests Pain Catastrophizing Scale (PCS) and Hospital Anxiety and Depression Score (HADS). The participants completed the PCS and HADS questionnaires and handed them back on the first experimental day in a concealed opaque envelope to ensure blinding. Opening of the envelopes were postponed until all participants had completed all four experimental days.

In order to investigate the inter-investigator variance, two different investigators were responsible for the testing on the different experimental days. Each participant was tested on four different study days. Two different investigators performed the testing. Every participant was thus tested by each investigator independently on two separate days—the order of the days being randomized.

The investigators were trained in performing the assessments similarly, but conducted the tests independently of each other. Test results were placed in an opaque sealed envelope to ensure blinding between the two investigators.

### Study participants

**Fig 1:**
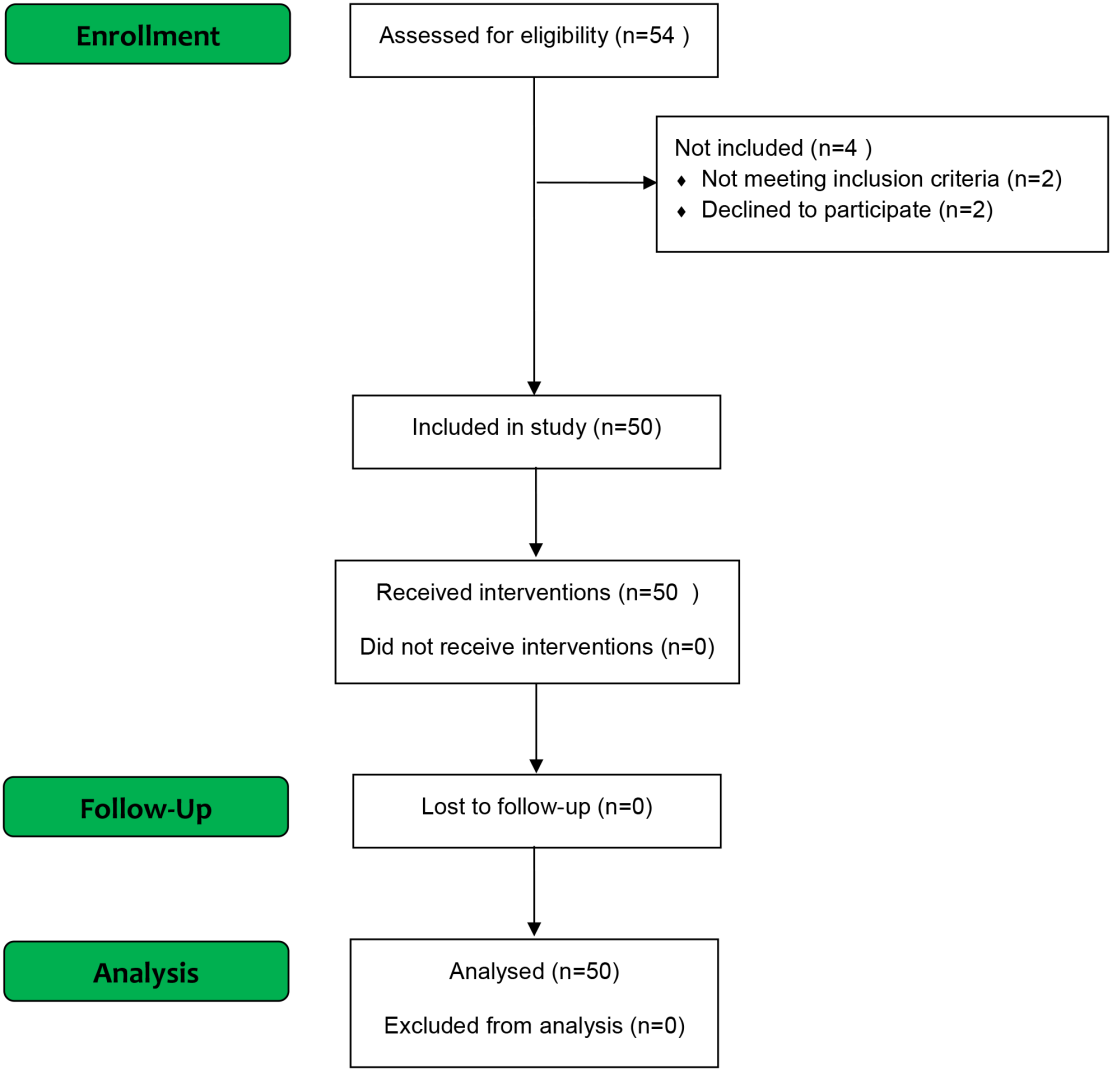
Flowchart of included study participants.

50 healthy male volunteers were included (Fig 1). Informed consent was obtained from all included participants. Participants were recruited by advertisement in the medical student magazine and online at www.forsøgspersoner.dk. Inclusion criteria were: Male sex, age ≥18 years and ≤35 years, speak and understand the Danish language, and signed informed consent. Exclusion criteria were: Failure to cooperate to the tests, alcohol and/or substance abuse, consummation of analgesics within 48 hours before experimental day, consummation of prescription medicine within the last 30 days before experimental day, history of chronic pain, psychiatric diagnoses, tattoos on the extremities, and a Body Mass Index (BMI) of >30 kg/m^2^ and < 18 kg/m^2^.

### Setting

The study was conducted in a quiet secluded room (temperature 22–25 degrees Celsius), where only the study participant and the responsible investigator were present. The participant was placed in a supine position during the assessment. The study was conducted during the time from 8.00 AM to 5.00 PM.

### Pain models

#### Brief thermal sensitization (BTS)

Induction of BTS was performed anterior on the right thigh, in the midline between the anterior superior iliac spine and the base of patella [4–8]. The skin was heated to 45°C for 3 min. After 3 min., while the 45°C thermode was still placed on the skin, the assessment of secondary hyperalgesia was performed (see section “assessment of secondary hyperalgesia”). The assessment of secondary hyperalgesia took approximately 1–2 minutes, with a maximum duration of heat stimulation of 5 min.

#### Pain during 1 min. thermal stimulation (p-TS)

Evaluation of p-TS was performed on the anterior aspect of the non-dominant volar side of the forearm [5, 21–24]. The participant’s skin was heated to 45°C for 1 min., while the participant performed continuous evaluation of pain on an electronic visual analogue scale (VAS, 0 mm = no pain; 100 mm = worst pain imaginable). A maximum (Max.)-VAS and a VAS-Area under the curve (AUC) were registered. The participant was not able to see the computer-screen during the assessment.

#### Heat pain detection threshold (HPDT)

The skin on the dominant anterior volar side of the forearm was heated with an increase in temperature of 1°C/sec (initial temperature 32°C) [5, 8, 21–24]. The study participant stated when the heat was perceived as painful by pressing a button, and the temperature was registered. The HPDT was calculated as an average of 4 stimulations. Each stimulation was performed with an interval of 6–10 seconds. The participant was not able to see the computer-screen during the assessment.

#### Assessment of secondary hyperalgesia

The area of secondary hyperalgesia was evaluated following BTS. The area was quantified by stimulation with a 19G monofilament (Von Frey hair) in 4 linear paths arranged 90° around the center of the heat-stimulation. The monofilament stimulation was initiated in normal skin, and advanced in steps of 5 mm with 1-second intervals towards the center of the heat-stimulation until the participant stated a clear change in the sensation (burning, intense pricking, tenderness). The borders were marked with a felt pen, and the transverse and longitudinal axes were measured with a pliable measuring tape for rectangular area calculation [4–8, 15, 21–27].

The area of primary hyperalgesia was defined as the area directly heated by the thermode (2.5x5 cm). The surrounding area with decreased mechanical thresholds was defined as the area of secondary hyperalgesia. For calculations, the area of the thermode (2.5x5 cm) was not subtracted from the total area of secondary hyperalgesia.

### Psychological testing

#### Hospital Anxiety and Depression Scale (HADS)

*HADS* is a questionnaire consisting of 14 questions [28]. HADS evaluates depression and anxiety, and can be subdivided in HADS-Anxiety and HADS-Depression. The highest achievable score is 42.

#### Pain Catastrophizing Score (PCS)

*PCS* is a questionnaire consisting of 13 questions [29] and can be subdivided into 3 subtests that each evaluates the central elements in catastrophizing: Rumination, magnification, and helplessness. The highest achievable score is 52.

### Outcomes

#### Primary outcome

To determine the intra- and inter-participant variance, and the intra- and inter-investigator variance of the secondary hyperalgesia areas following BTS on 4 separate experimental days with two different observers.

#### Secondary outcomes

To investigate:

1. How precise the scores of PCS and HADS predict the size of the area of secondary hyperalgesia.
2. How precise the subscales in PCS and HADS (PCS-rumination, PCS-magnification, PCS-helplessness, and HADS-Anxiety, HADS-Depression) predict the size of the area of secondary hyperalgesia.
3. How precise the HPDT evaluated on the 4 experimental days predicts the area of secondary hyperalgesia on the respective 4 experimental days.
4. How precise the Maximum VAS-score following p-TS evaluated on the 4 experimental days predicts the area of secondary hyperalgesia on the respective 4 experimental days.
5. How precise the VAS-AUC following p-TS evaluated on the 4 experimental days predicts the area of secondary hyperalgesia on the respective 4 experimental days

### Sample size estimation

Estimation of the number of participants, investigators and experimental days were based on statistical simulations based on data from a previous study [4]. The simulations demonstrated that a scenario with 2 investigators, 4 experimental days and 50 study participants would enable us to discern the relevant variance components with acceptable precision.

For full documentation of statistical simulations, please see supporting information available online (S1 Appendix).

### Randomization and allocation concealment

**Fig 2:**
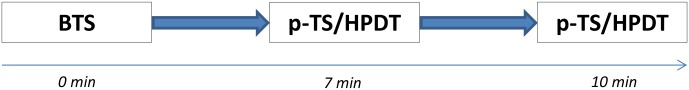
Sequence of clinical pain stimulation. Sequence of clinical pain stimulation. Sequence of p-TS and HPDT depends on randomization. Abbreviations: BTS, brief thermal sensitization; p-TS, pain during 1 min. thermal stimulation; HPDT, heat pain detection threshold; min, minutes.

The reproducibility of the area of secondary hyperalgesia following BTS was the primary outcome. Thus, in order to avoid possible carry over effects of the HPDT and p-TS, all study days began with BTS. Testing with HPDT and p-TS were therefore subsequent to BTS on all study days; However, the sequence of testing (HPDT and p-TS) was randomized for each patient and each experimental day, so that on two of the four experimental days the sequence was: 1) BTS, 2) HPDT, 3) p-TS, and on the remaining two study days the sequence was: 1) BTS, 2) p-TS, 3) HPDT (Fig 2).

The investigator responsible for testing and registration of data on the respective experimental day was randomized, so the same investigator was not responsible for testing the same participant on two consecutive experimental days. The allocation sequence of participants to investigator, and the test allocation sequence (HPDT and p-TS) were randomly generated via a computer by the data manager at Copenhagen Trial Unit.

The randomization of the test allocation sequence was kept in opaque sealed envelopes prepared by Copenhagen Trial Unit to ensure allocation concealment. The envelopes remained sealed until immediately before the testing.

### Statistical analysis

The variation in the areas of secondary hyperalgesia derived from the study participant, the experimental day, and the investigator was determined using a variance component model.

For each of the 5 secondary outcomes, the ability of PCS, HADS, HPDT, Max-VAS and VAS-AUC (following p-TS), to predict individual variations in areas of secondary hyperalgesia was investigated by linear regression on the estimated best linear unbiased predictors (EBLUPS) of individual secondary hyperalgesia extracted from the primary analysis. HPDT, Max-VAS, and VAS-AUC profiles were also summarized in terms of EBLUPS.

Significance of the predictors was assessed by Analysis of variance (ANOVA) methods and their predictive abilities were quantified with various summaries of prediction errors including 95% prediction intervals for the predictions. Model reduction was done by means of backwards elimination with a cut-off value of 5%.

The variation in the areas of secondary hyperalgesia described in the primary outcomes will be reported as Intraclass Correlations (ICC) and Coefficient of Variations (CV).

The predictive abilities of the variables described in the secondary outcomes are summarized by adjusted R^2^ and illustrated by 95% predictive intervals for selected values of the remaining predictor.

### Reproducibility of area of secondary hyperalgesia

We planned to categorize the participants in three groups according to the mean size of the area of secondary hyperalgesia: “Small-area” (1^st^ quartile), “medium-area” (2^nd^ and 3^rd^ quartile), and “large-area” (4^th^ quartile).

Based on the study performed by Werner et al. [15] we expected measures of reproducibility as detailed below:

1. Intraclass correlation coefficient (ICC) around 0.74, corresponding to an intra-participant variance of approximately 25% of the inter-individual variance.
2. A pooled mean intra-participant CV around 0.25
3. No more than 10% of the study participants change group from “small-area” to “large-area” or vice versa

## Results

All study participants completed the study, and all study participants were analyzed for primary and secondary outcomes (Fig 1).

Data on participants’ characteristics are presented in Table 1. The median interval between the information day, experimental day 1, 2, 3 and 4 was 12 (Range; 7–33), 9 (7–35), 15 (7–61) and 8.5 (7–37) days respectively.

**Table 1:** Characteristics of included participants.

Variable	Mean (SD)	Range (min.-max.)
Age (years)	24 (3)	18–32
Height (m)	1,85 (0,1)	1.69–1.98
Weight (kg)	78 (9)	65–105
BMI (m^2^/kg)	22,8 (2,1)	18.4–27.3

Abbreviations: BMI, Body Mass Index

No adverse or serious adverse events were reported.

The results from this study has previously been presented in abstract form [30].

### Secondary hyperalgesia following BTS

The secondary hyperalgesia following BTS was evaluated on the 4 separate experimental days by 2 different investigators (1 investigator per experimental day). We found (i) an intra-investigator intra-participant correlation of 0.85, 95% CI (0.78, 0.90), (ii) an inter-investigator intra-participant correlation of 0.82 (0.69, 0.89), (iii) an intra-investigator inter-participant correlation of 0.03 (0.00, 0.16), and (iv) a coefficient of variation of 0.17 (0.14, 0.21).

**Fig 3:**
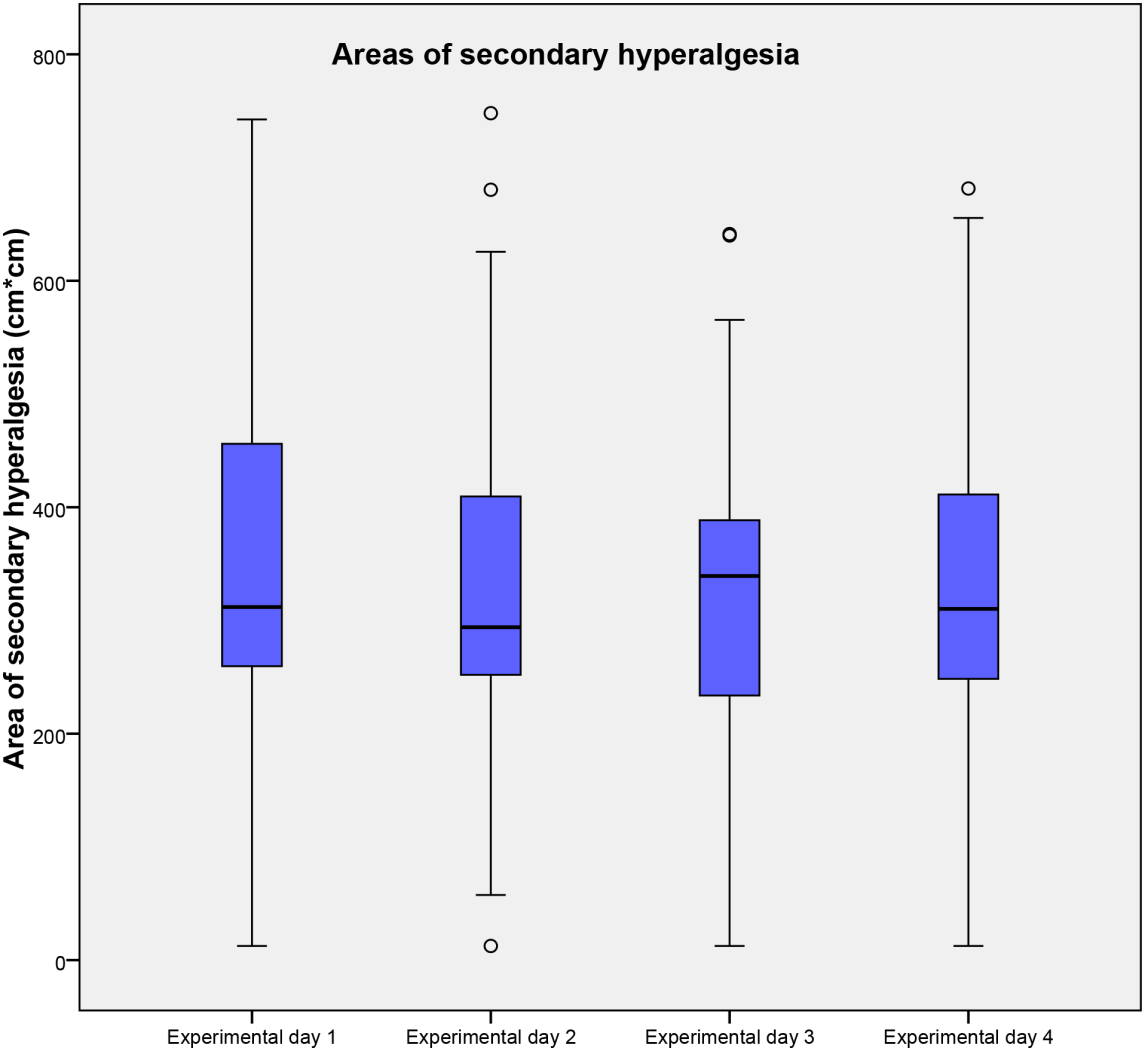
Areas of secondary hyperalgesia following BTS. Areas of secondary hyperalgesia on the 4 experimental days following brief thermal sensitization. Medians and interquartile ranges are displayed. Values higher than 1.5 times of upper quartile or lower quartile are designated as outliers and marked with °. Abbreviations: Cm, centimeter; BTS, Brief thermal sensitization.

Only 2 participants, 4% (1%, 13%) of the total population, had areas of secondary hyperalgesia in both below the 1^st^ quartile and above the 3^rd^ quartile, considering the total population. The sizes of the areas of secondary hyperalgesia as well as the results of the ICC are presented in Tables 2 and 3 and Fig 3, respectively.

**Table 2:** Median size of the area of secondary hyperalgesia.

QST	Experimental day 1		Experimental day 2		Experimental day 3		Experimental day 4	
	Median (IQR)	Range	Median (IQR)	Range	Median (IQR)	Range	Median (IQR)	Range
BTS*	311.9 (256.6–457.0)	12.5–742.5	294.0 (250.5–417.3)	12.5–748.0	339.3 (231.7–389.9)	12.5–641.3	310.1 (244.1–413.3)	12.5–681.5

Median size and range of areas of secondary hyperalgesia following BTS on the four experimental days

* Medians, IQRs and ranges are given in Cm^2^

Abbreviations: QST, Quantitative Sensory Testing; BTS, Brief thermal sensitization, IQR, Interquartile range

**Table 3:** Main results.

Parameter	Result (95% CI)
ICC_Intra-investigator intra-participant_	0.85 (0.78–0.90)
ICC_Inter-investigator intra-participant_	0.82 (0.69–0.89)
ICC_Intra-investigator inter-participant_	0.03 (0.0–0.16)
CV	0.17 (0.14–0.21)

Intraclass Correlations and Coefficient of Variation. Abbreviations: ICC, Intra Class Correlation; CV, Coefficient of Variation

### Predictive factors of the area of secondary hyperalgesia

**Fig 4:**
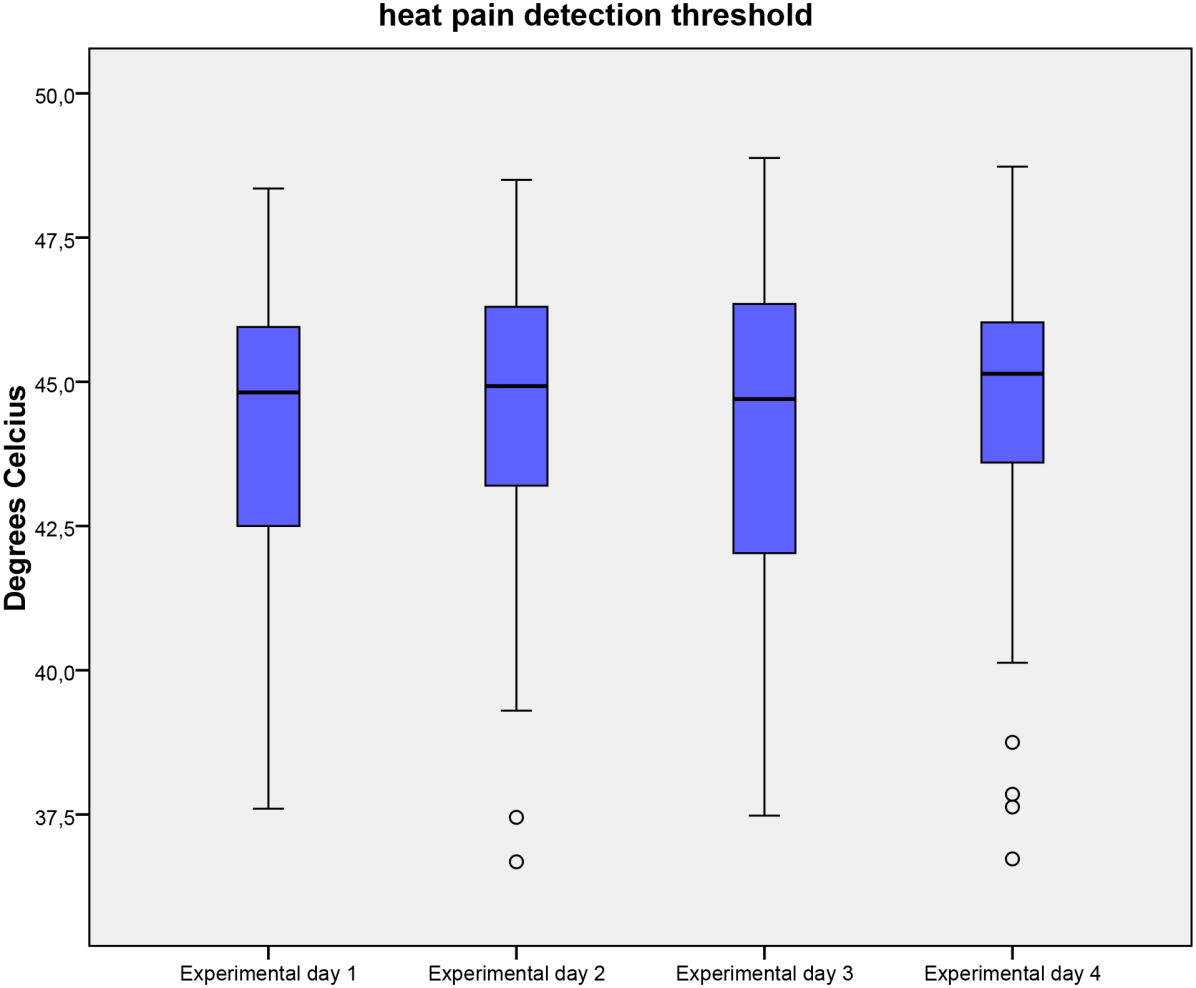
Heat pain detection threshold. Heat pain detection threshold on the 4 experimental days. Medians and interquartile ranges are displayed. Values higher than 1.5 times of upper quartile or lower quartile are designated as outliers and marked with °.

**Fig 5:**
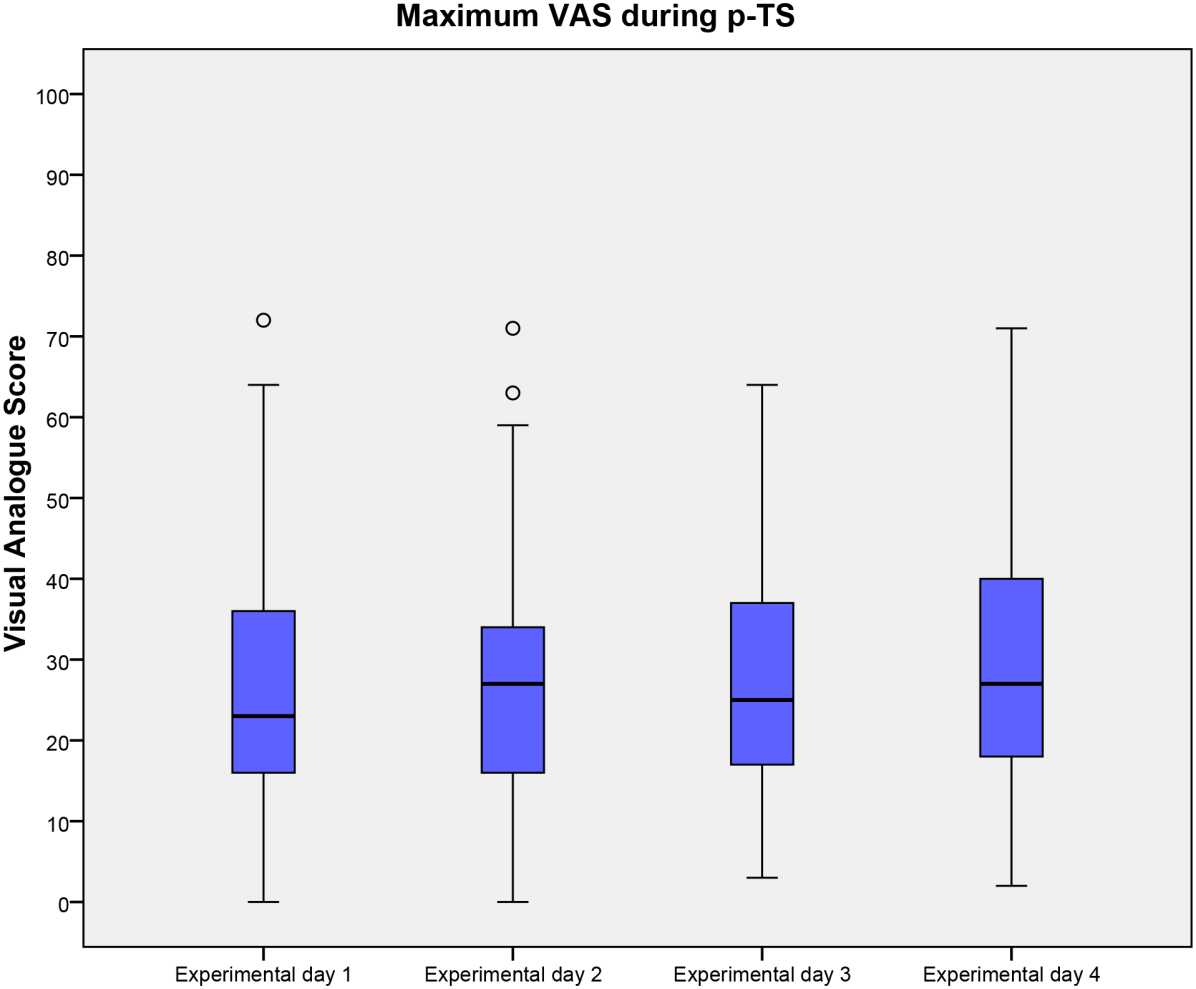
maximum VAS during p-TS. Maximum visual analogue score during 1 min. thermal stimulation on the 4 experimental days. Medians and interquartile ranges are displayed. Values higher than 1.5 times of upper quartile or lower quartile are designated as outliers and marked with °. Abbreviations: VAS, Visual analogue score; p-TS, Pain during 1 min. thermal stimulation.

**Fig 6:**
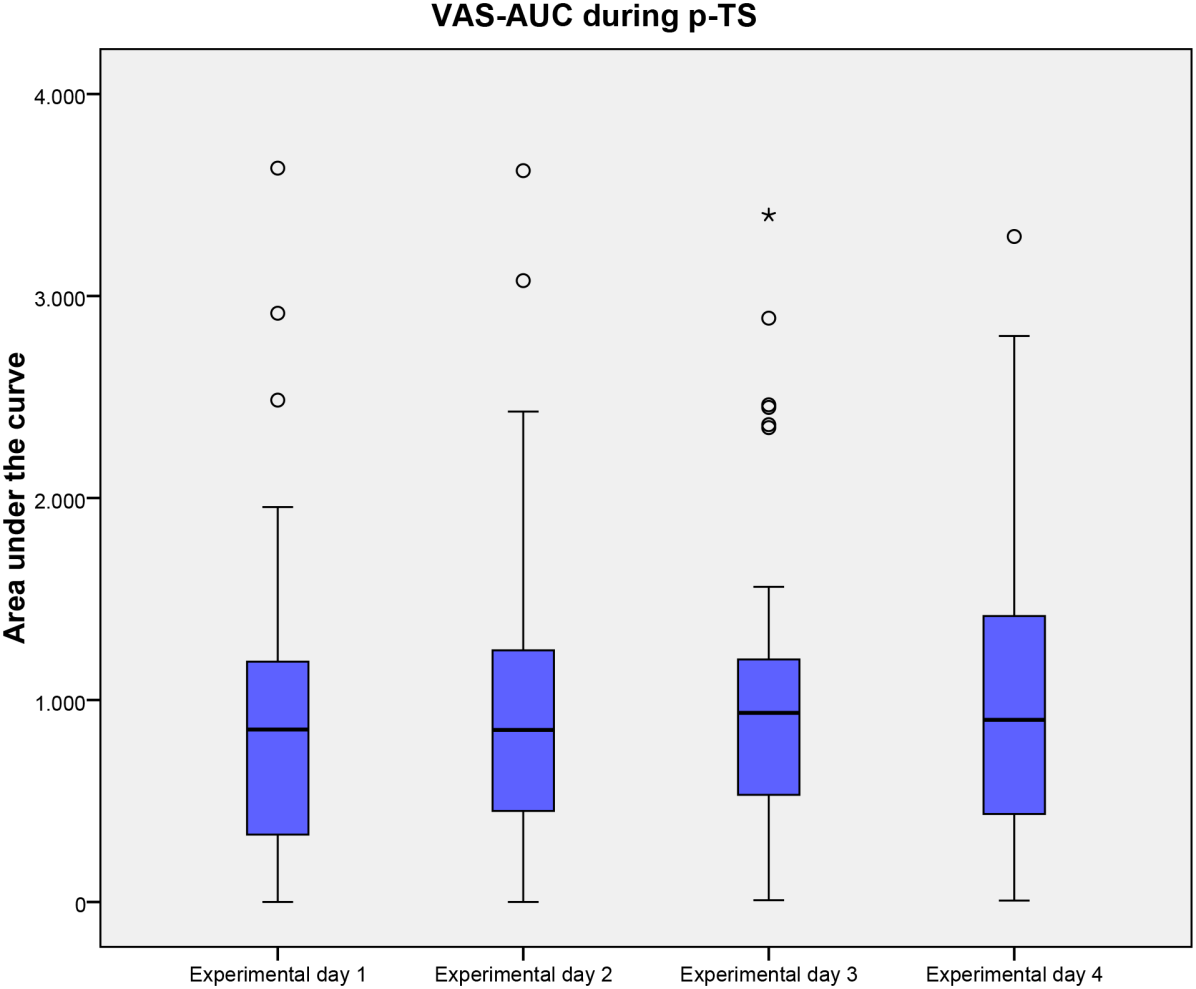
VAS-AUC during p-TS. Visual analogue score during 1 min. thermal stimulation on the 4 experimental days. Medians and interquartile ranges are displayed. Values higher than 1.5 times of upper quartile or lower quartile are designated as outliers and marked with °. Abbreviations: VAS, Visual analogue score; p-TS, Pain during 1 min. thermal stimulation; AUC, Area under the curve.

The ability of HPDT, Max-VAS (following p-TS), VAS-AUC (following p-TS), PCS and HADS to predict inter-individual variations in area of secondary hyperalgesia was investigated. After backwards elimination with a cut-off value of 5% only HPDT offered a statistically significant prediction of the area of secondary hyperalgesia with an adjusted R^2^ of 0.20 (P = 0.0006). No other evaluated factors significantly predicted the area of secondary hyperalgesia. Results of HPDT, Max-VAS (following p-TS), VAS-AUC (following p-TS), PCS and HADS are presented in Tables 4 and 5 and Figs 4, 5 and 6.

**Table 4:** Heat pain detection threshold, and pain during 1 min. thermal stimulation.

QST	Experimental day 1		Experimental day 2		Experimental day 3		Experimental day 4	
	Median (IQR)	Range	Median (IQR)	Range	Median (IQR)	Range	Median (IQR)	Range
HPDT*	44.8 (42.4–46)	37.6–48.4	44.9 (43.1–46.3)	36.7–48.5	44.7 (42–46.4)	37.5–48.9	45.1 (43.6–46)	36.7–48.7
p-TS-max. VAS	23.0 (16–36)	0.0–72.0	27.0 (16–34)	0.0–71	25.0 (16.8–37.3)	3.0–64.0	27.0 (17.5–40)	2.0–71.0
p-TS-AUC VAS	853.8 (325.2–1198)	0.0–3663.0	851.8 (448.3–1275.3)	0.0–3620.50	936.2 (523.4–1216.2)	8.8–3400.7	901.7 (421.1–1416.6)	6.9–3294.5

Median, IQR and range of HPDT, p-TS-max VAS and p-TS-AUC VAS on the 4 experimental days.

* Medians, IQRs and ranges are given in °C

Abbreviations: QST, Quantitative Sensory Test; HPDT, Heat pain detection threshold; p-TS, pain during 1 min. thermal stimulation, Max., maximum; AUC, Area Under the Curve; VAS, Visual Analog Scale, IQR, Interquartile range

**Table 5:** Scores of Pain Catastrophizing Scale and Hospital Anxiety and Depression Scale.

Psychological test	Median (IQR)	Range
PCS_Rumination_	6 (3.8–8)	0–15
PCS_Magnification_	2 (1–4)	0–7
PCS_Helplessness_	3 (2–7)	0–19
PCS_Total_	12 (7.8–18)	0–38
HADS_Anxiety_	4 (1–6)	0–12
HADS_Depression_	1 (1–3)	0–9
HADS_Total_	5 (3–10)	0–18

Median, IQRs and range of the PCS and HADS. Total scores and scores of individual subtests are displayed. Abbreviations: PCS, Pain Catastrophizing Score; HADS, Hospital Anxiety and Depression Scale, IQR, Interquartile range

## Discussion

The aim of this study was to investigate the intra-individual, inter-individual, and inter-investigator variances of BTS-elicited areas of secondary hyperalgesia, in order to examine the reproducibility of the pain model. There are no gold standards for reproducibility; however, prior to our study we defined three criteria, hypothesized using data from previous studies. In order to confirm the reproducibility of the model, all three criteria had to be fulfilled (see methods). Firstly, we demonstrated an inter-investigator intra-participant correlation of 0.82 (0.69, 0.89), secondly we found a coefficient of variation of 0.17 (0.14, 0.21), and lastly we demonstrated that only two participants, 4% (1%, 13%) of the total population, had areas of secondary hyperalgesia in both below the 1^st^ quartile and above the 3^rd^ quartile, considering the total population. By evaluating the point estimates, all three criteria are fulfilled, and according to our pre-defined criteria, we have demonstrated that BTS is a reproducible model in regards to eliciting secondary hyperalgesia in healthy volunteers. The rather high ICCs and low CV demonstrate a high reliability and reproducibility respectively.

We also demonstrated that HPDT significantly predicted the area of secondary hyperalgesia. Our study was not designed to detect the correlation between HPDT and areas of secondary hyperalgesia; however, we find a highly significant result (P = 0.0006). An adjusted R^2^ of 0.20 is nonetheless an indication that HPDT only offers a very modest explanation of the variation in BTS, which poses the question: How precise does HPDT predict areas of secondary hyperalgesia? In the current study, we estimated a prediction interval for BTS to (53.14–515.43) given a HPDT of 46°C, indicating wide prediction intervals. The possible variation of BTS for a given HPDT value is huge and HPDT and secondary hyperalgesia following BTS may thus represent two different pain entities. HPDT has been demonstrated to be highly reproducible [31], and both HPDT and brief thermal sensitization activates peripheral A-delta and C-fibers [32]. However, the secondary hyperalgesia to punctate mechanical stimuli, that occurs as a result of central neuronal plasticity of the nociceptive system is mediated by A-fiber nociceptors, not C-fibers [11–14]. Thus, secondary hyperalgesia as a result of central sensitization elicited by BTS may be significantly distinct from HPDT. To our knowledge, no studies have investigated this issue, and further studies are needed to confirm this hypothesis.

Individual characteristics, such as sex, and obesity, may influence pain thresholds and tolerance [24, 33–37]. Likewise it remains unclear whether the menstrual cycle influences the pain sensitivity in healthy women [38]. In addition we have no knowledge of what effect tattoos have on peripheral cutaneous sensitivity. To account for these variables, we applied strict inclusion and exclusion criteria, in order to minimize the unknown factors of variation. This enabled us to focus on the ability of BTS to produce an area of secondary hyperalgesia, rather than the influence of the individual characteristics of the participant.

Several clinical studies have demonstrated an association between psychological factors and pain [39, 40]. In the present study the two psychological tests, HADS and PCS, did not significantly predict the area of secondary hyperalgesia. Our study was not designed to detect the correlation between psychological test scores and area of secondary hyperalgesia; however, post hoc analyses demonstrated that in order to investigate such a correlation with sufficiently high power, 300 healthy participants should have been included in the study. A plausible reason for the weak correlation between psychological test scores and secondary hyperalgesia may be that we included a very homogenous population of healthy volunteers, that all had relatively low scores on HADS and PCS. By including healthy volunteers in an experimental pain study, it can be speculated that only few persons with a high anxiety-index or high-catastrophizing scores are enrolled, because they rarely consider volunteering in experimental pain studies. Thus, avoidance of sampling bias may be difficult. Therefore, in order to minimize sampling bias when investigating a possible association of high psychological vulnerability and experimental pain entities, consecutive inclusion of patients awaiting surgery, or specific inclusion of healthy volunteers with high psychological vulnerability seems necessary.

When evaluating the area of secondary hyperalgesia there exist different methods [4–8, 15, 18, 21–27, 41]. We chose a pragmatic approach that can be easily applied in a clinical setting by non-specialists, and evaluated the area of secondary hyperalgesia with the same polyamide filament (19g), in all the participants. Likewise, we chose a simple approach in calculating the area of secondary hyperalgesia, by using a 4 vector rectangular area calculation that has been applied in several previous studies [4–8, 15, 21–27]. More favorable results may have been achieved by using the up-down method when applying punctate mechanical stimuli with the polyamide monofilaments [42]; however, our results demonstrate that the methods we applied were sufficient to demonstrate that BTS produce a reproducible area of secondary hyperalgesia.

The area of secondary hyperalgesia elicited by BTS may represent the level of central sensitization in the individual participant. Woolf describes that different pain hypersensitivity syndromes may share a common contribution of central sensitization, and hypothesizes that the comorbidity of different clinical pain syndromes may be explained by a “central sensitization syndrome” [11]. Thus, individuals with high pain sensitivity may share common factors that may be identified prior to the development of chronic pain. Moreover, it unlocks the possibility that individuals can be “phenotyped” in regards to their pain hypersensitivity. The assessment of secondary hyperalgesia may be a tool for investigation of central sensitization, and thus, be applied as a predictive factor of e.g. postoperative pain. To our knowledge, only few studies have investigated the assessment of secondary hyperalgesia as a possible predictive factor of postoperative pain, with one study demonstrating no correlations between area of secondary hyperalgesia following burn injury [43], and another demonstrating that postoperative secondary hyperalgesia around the surgical incision following an iliac crest bone harvest was predictive for the development of chronic postsurgical neuropathic pain [44]. Our results demonstrate a high inter-participant variance in the area of secondary hyperalgesia. The primary aim with this study was not to identify possible causal factors that could explain the high inter-participant variance; however our results are interesting and several factors could provide explanation for the remarkable inter-participant variance in an otherwise homogenous population. Factors such as stress [45], diet, including tryptophan intake [46, 47], hormone levels [45], skin receptor density, anatomical and functional brain differences [18], as well as genetics [16] could have influenced our results. The area of secondary hyperalgesia as a phenotypic indicator of pain hypersensitivity, and as a predictor for the development of acute and chronic pain is yet unexplored and further research is needed in order to clarify this.

The applicability of quantitative sensory testing (QST) and experimental pain models in translational and clinical studies has been widely debated. Should QST be implemented in the daily clinical practice, and should trials investigating analgesics implement the use of experimental pain models? When evaluating the increasingly body of research performed on QST and pain models, the main problem appears to be the heterogeneity in study methodology and statistical approaches [1, 48]. When using QST it is recommend that intra-participant reliability is determined [19]. Thus, before QST and experimental pain models can be fully implemented in clinical studies, reliability of the individual models is necessary. This means that several prospective methodological studies of the individual models should be performed [49]; evaluating intra- and inter-participant variance, as well as intra- and inter-investigator variance. Moreover, general accepted measures of reproducibility are needed. So far, no general recommendations have been proposed on how reproducible or reliable the various pain models or QSTs should be before they are implemented in translational or clinical research [50]. In the present study, we attempted to pre-define measures of reproducibility based on an earlier retrospective study [15]. This is in our opinion an important strength, and hypotheses regarding reproducibility/reliability should be implemented in study design as well as sample size analysis in future studies. If a test is not reliable and/or reproducible, then it cannot be used as tool in a diverse scientific community.

Our study has some limitations. Firstly, the two investigators were trained to perform BTS in precisely the same manner. That includes the assessment of secondary hyperalgesia, as well as the information given to the participants. This means, that if two entirely independent investigators were to perform the assessment without rigorous simultaneous training, the inter-investigator variance might increase. Moreover, even though our simulation study (see supporting information: S1 Appendix, simulation study 2, Figs 4 and 5) demonstrated that a scenario with 2 investigators and 50 study participants would enable us to discern the relevant variance components with acceptable precision, a higher number of independent observers is required to obtain final conclusions on the inter-observer variance and inter-study comparisons.

Secondly, we applied BTS on a highly homogenous population of healthy, male volunteers. Inclusion of women, elderly patients and chronic pain patients could potentially have increased the inter-participant variance with a clustering of effects in chronic pain patients. Thus, studies investigating a heterogeneous clinical population are needed in order to clarify the potential of BTS as a tool for evaluating both male and female patients, as well as the young and elderly population. Thirdly, we did not evaluate the participants’ dietary intake or hormone levels. Studies have demonstrated that tryptophan may increase the pain sensitivity [46, 47], and cortisol and testosterone levels may influence the pain sensitivity [45]. Consequently, control of the dietary intake may have decreased intra-participant variance, and evaluation of hormone levels may have been an explanatory factor in the high inter-participant variance in the area of secondary hyperalgesia.

Lastly, even though our HPDT was within the range reported in previous studies [51], it may be that the three participants with a mean HPDT below 40°C possibly misunderstood the procedure.

In conclusion, our rigorous prospective study confirms earlier retrospective indications, that BTS produce a reproducible area of secondary hyperalgesia [15]. Furthermore, we have demonstrated a low intra-participant variance and a high inter-participant variance compared to inter-observer variance. BTS can therefore be applied in investigations of secondary hyperalgesia in healthy volunteers. However, to thoroughly determine that BTS is a reliable tool for pain research, other independent research groups should continue investigation of BTS in healthy volunteers and in more heterogeneous clinical populations.

## Supporting Information

S1 Appendix Statistical simulation study. (PDF)

S2 Appendix All relevant study data. (XLSX)

S3 Appendix Trend Checklist. (PDF)

S4 Appendix Study protocol, original language, Danish. (DOCX)

S5 Appendix Study protocol, translated, English. (DOCX)
