# Correction: The Area of Secondary Hyperalgesia following Heat Stimulation in Healthy Male Volunteers: Inter- and Intra-Individual Variance and Reproducibility

**DOI:** 10.1371/journal.pone.0176081

**Published:** 2017-04-13

**Authors:** Morten Sejer Hansen, Jørn Wetterslev, Christian Bressen Pipper, Rebecca Østervig, Mohammad Sohail Asghar, Jørgen Berg Dahl

There is an error in the second sentence under the sub-heading “Assessment of secondary hyperalgesia.” within the “Pain models” section of the “Material and Methods”. The correct sentence is: The area was quantified by stimulation with a monofilament (Von Frey hair) with a nominal value of 18 (bending force of 490 mN) in 4 linear paths arranged 90° around the center of the heat-stimulation.
