# Supplementary material for: The Area of Secondary Hyperalgesia following Heat Stimulation in Healthy Male Volunteers: Inter- and Intra-Individual Variance and Reproducibility
Source: PLoS One. 2016 May 11;11(5):e0155284. doi: 10.1371/journal.pone.0155284 (PMC4864410; doi:10.1371/journal.pone.0155284)
Supplement: S1 Appendix — (PDF) [file pone.0155284.s001.pdf]

Department of Biostatistics, University of Copenhagen

May 8, 2014
2013
CP

Internal report I version 2

A simulation study on the precision of measures of reproducibility for the area of hyperalgesia with BTS stimulation

Morten Sejer Hansen (RH)

Contents

1 Simulation design **1**

2 Estimation **1**

3 Measures of Reproducibility **2**

3.1 Intra class correlation 2

3.2 Coefficient of variation 2

3.3 Root mean squared error of EBLUPS 2

4 Obtaining parameter values for the simulations **3**

4.1 The methods data 3

4.2 The gaba data 5

4.3 The final choice of parameter-values 6

5 Results: Simulation study 1 **7**

5.1 Results for ICC 8

5.2 Results for CV 10

5.3 Results for RMSE 12

6 Results: Simulation study 2 **14**

6.1 Results for ICC 16

6.2 Results for CV 18

6.3 Results for RMSE 20

7 Appendix: R code **20**

Christian Pipper

Internal reports are preliminary working papers which are confidential unless an explicit agreement to the contrary has been reached between the clients and the author of the report.

1 Simulation design

We consider n persons measured at m days. For each day and person 1 out of l observers is assigned randomly so that the same observer does not observe the same person at two consecutive days. For the ith person on the jth day we simulate the response according to the model

$$Response_{ij} = \mu + U_i + V_{Observer(ij)} + \varepsilon_{ij}, \tag{1}$$

where μ is the overall level of the response, $U_i \sim N(0, \sigma^2_{person})$ are the random effects corresponding to person, $V_{Observer(ij)} \sim N(0, \sigma^2_{observer})$ are the random effects corresponding to observer, and $\epsilon_{ij} \sim N(0, \sigma^2_e)$ are the measurement errors

For the p percent persons with the largest responses on the first day responses on the subsequent days are set to be missing.

All simulations are conducted in R version 3.0.2 (www.r-project.org)

2 Estimation

For a given simulated data set Estimates of μ,σ_{person},$\sigma_{observer}$, and σ_e are obtained by REML estimation based on the model (1). Furthermore the random person effects U_i are estimated by the corresponding EBLUPS $\hat{U}_i$.

All analyses are conducted with the R-function `lmer()` available from the `lme4` add-on R package.

3 Measures of Reproducibility

3.1 Intra class correlation

In the context of this design we are looking at the intra person correlation when comparing measurements from the same person on different days (and accordingly with different observers). In terms of the parameters of the model this is defined as

$$ICC = \frac{\sigma^2_{person}}{\sigma^2_{person} + \sigma^2_{observer} + \sigma^2_e}$$

This quantity is easily obtained by plugging in estimated model parameters

3.2 Coefficient of variation

This is given as

$$CV = \frac{\sigma_e}{\mu}$$

and may be estimated by plugging in estimated model parameters. Note that CV as defined here corresponds to relating the standard deviation of two observations from the same person with the same observer to their expected value. Alternatively one could consider measurements from the same person with different observers. Then the coefficient of variation would be expressed as

$$CV_{alt} = \frac{\sqrt{\sigma^2_e + \sigma^2_{observer}}}{\mu}$$

3.3 Root mean squared error of EBLUPS

To assess how accurately we may estimate person characteristics in terms of the random person effect U_i we may consider the root mean squared error given by

$$RMSE = \sqrt{\frac{1}{n}\sum_{i=1}^{n}(\hat{U}_i - U_i)^2}.$$

Note that it is not possible to calculate this quantity without knowing the true values of the random person effects

4 Obtaining parameter values for the simulations

4.1 The methods data

To obtain values of μ,σ_{person},$\sigma_{observer}$, and σ_e we analysed the methods data for which data on the first person is listed below.

	hyperalg	day	treatment	period	person
1	223	1	1	1	1
2	219	1	1	2	1
3	216	1	1	3	1
4	257	2	2	1	1
5	219	2	2	2	1
6	191	2	2	3	1
7	155	3	3	1	1
8	226	3	3	2	1
9	134	3	3	3	1
10	104	4	1	1	1
11	232	4	1	2	1
12	267	4	1	3	1

On these data we fitted a linear mixed model including a combined effect of treatment and period and random effects of person and person within day. The resulting model fit is shown below

```
> fit<-lmer(hyperalg~-1+factor(treatment):factor(period)+(1|person)+(1|person*day),
+ data=anadata)
> summary(fit)
Linear mixed model fit by REML
Formula: hyperalg ~ -1 + factor(treatment):factor(period) +(1 | person) + (1 | person * day)
```

```
   Data: anadata
  AIC  BIC logLik deviance REMLdev
 2688 2729  -1332     2729    2664
Random effects:
 Groups       Name        Variance Std.Dev.
 person * day (Intercept)   736.18  27.133
 person       (Intercept) 14633.45 120.969
 Residual                  3384.48  58.176
Number of obs: 240, groups: person * day, 50; person, 20

Fixed effects:
                                   Estimate Std. Error t value
factor(treatment)1:factor(period)1   183.43      28.93   6.341
factor(treatment)2:factor(period)1   232.84      30.52   7.630
factor(treatment)3:factor(period)1   204.16      30.56   6.680
factor(treatment)1:factor(period)2   218.63      28.93   7.558
factor(treatment)2:factor(period)2   238.14      30.52   7.804
factor(treatment)3:factor(period)2   223.81      30.56   7.323
factor(treatment)1:factor(period)3   228.98      28.93   7.916
factor(treatment)2:factor(period)3   263.54      30.52   8.636
factor(treatment)3:factor(period)3   246.96      30.56   8.080
```

From this we extract the overall mean as the average level in the first period of a trial day, that is:

$$\mu = (183.43 + 232.84 + 204.16)/3 \simeq 207$$

Moreover we extract

$$\sigma_{person} \simeq 121,$$

$$\sigma_e \simeq 58.$$

Finally, assuming that all random day to day variation within person can be ascribed to different observers we extract

$$\sigma_{observer} \simeq 27.$$

4.2 The gaba data

To partially validate the above parameter values we also analysed the so called gaba data. Datalines corresponding to the first person in this data set are given below

```
   personid treatment day hyperalg
1         1         1   1     80.9
26        1         2   2    100.5
```

These data were analysed with a mixed linear model including a combined effect of treatment and day and a random effect of person. The resulting model fit is shown below

```
> fit2<-lmer(hyperalg~-1+factor(day):factor(treatment)+(1|personid),
+ data=anadata2)
> summary(fit2)
Linear mixed model fit by REML
Formula: hyperalg ~ -1 + factor(day):factor(treatment) + (1 | personid)
   Data: anadata2
   AIC   BIC logLik deviance REMLdev
 550.1 561.6 -269.1      570   538.1
Random effects:
 Groups   Name        Variance Std.Dev.
 personid (Intercept) 9889.2   99.445
 Residual             1514.9   38.922
Number of obs: 50, groups: personid, 25
```

```
Fixed effects:
                                    Estimate Std. Error t value
factor(day)1:factor(treatment)1       194.61      26.70   7.290
factor(day)2:factor(treatment)1       206.61      35.59   5.805
factor(day)1:factor(treatment)2       210.07      35.59   5.902
factor(day)2:factor(treatment)2       226.58      26.70   8.487
```

From this we extract the overall mean as the level for day=1 treatment=1, that is

$$\mu \simeq= 195$$

Moreover we extract

$$\sigma_{person} \simeq 99,$$
$$\sigma_e \simeq 39.$$

4.3 The final choice of parameter-values

Based on the above analyses we conduct our simulations with the following parameter values

$$\mu = 200,$$
$$\sigma_{person} = 110,$$
$$\sigma_{observer} = 30,$$
$$\sigma_e = 50.$$

For reference, the above parameter values correspond to an ICC value of 0.78 and a CV value of 0.25.

5 Results: Simulation study 1

We considered the 6 scenarios:

- Scenario 1: $l = m = 4$, $p = 0$. Corresponding to 4 observers, 4 trial days per person, and no missing observations
- Scenario 2: $l = m = 7$, $p = 0$. Corresponding to 7 observers, 7 trial days per person, and no missing observations
- Scenario 3: $l = m = 10$ and $p = 0$. Corresponding to 10 observers, 10 trial days per person, and no missing observations
- Scenario 4: $l = m = 4$ and $p = 0.1$. Corresponding to 4 observers, 4 trial days per person, and 10% of the persons in the trial with missing observations after trial day 1. observations
- Scenario 5: $l = m = 7$ and $p = 0.1$. Corresponding to 7 observers, 7 trial days per person, and 10% of the persons in the trial with missing observations after trial day 1.
- Scenario 6: $l = m = 10$ and $p = 0.1$. Corresponding to 10 observers, 10 trial days per person, and 10% of the persons in the trial with missing observations after trial day 1.

all with n ranging from 20 to 100 in steps of 5.

For each scenario and value of n 1000 data sets were generated. For each data set values of ICC, CV, and $RMSE$ were calculated as described above.

5.1 Results for ICC

Below we present a summary of the simulations with respect to ICC

Scenario	Sample mean	Sample bias	Sample standard deviation
1	0.7794726	-0.001172594	0.05303596
2	0.7756883	-0.004956840	0.04645277
3	0.7781922	-0.002452985	0.04341995
4	0.7773741	-0.003271055	0.05780629
5	0.7724449	-0.008200232	0.04868458
6	0.7739216	-0.006723540	0.04733749

Table 1: Simulation summaries for ICC in the 6 scenarios with 50 persons in the study

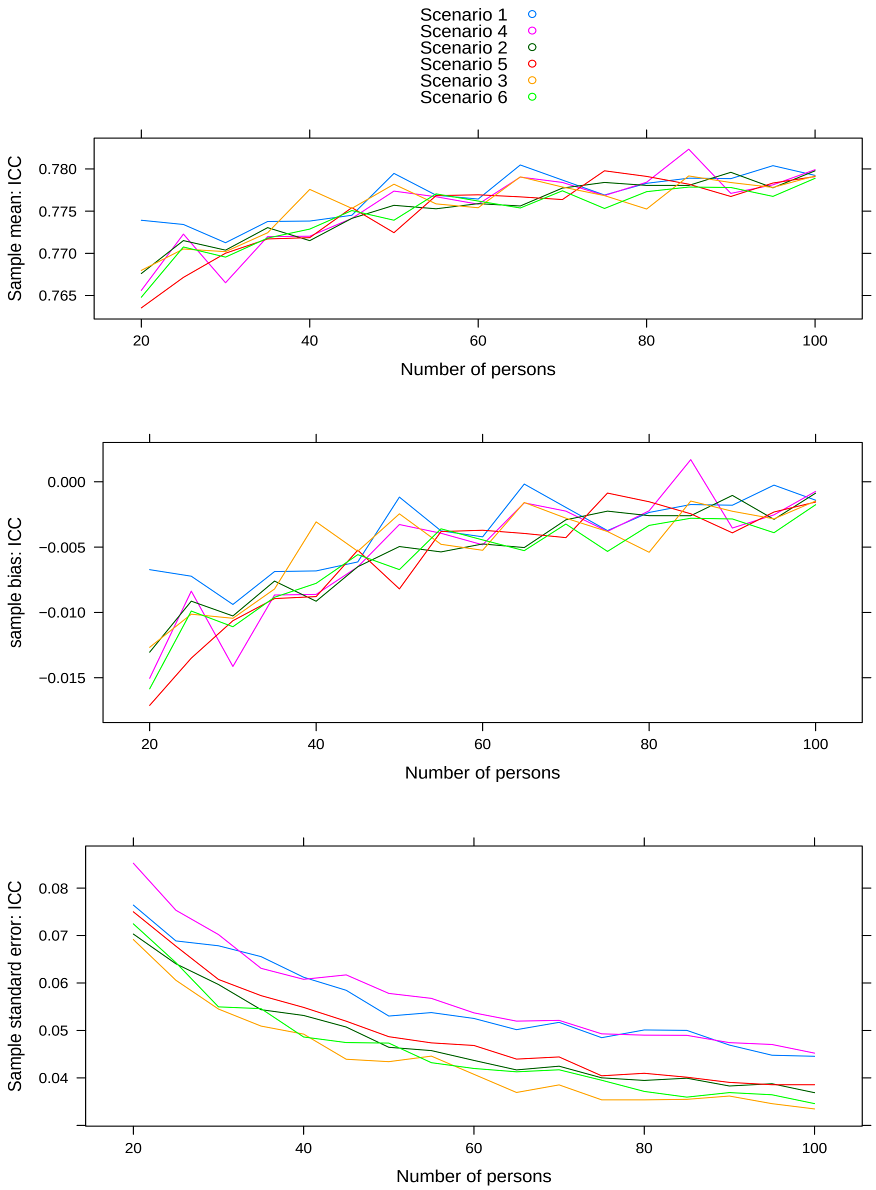

Figure 1: Simulation summaries for ICC in the 6 scenarios as a function of number of persons in the study

5.2 Results for CV

Below we present a summary of the simulations with respect to CV

Scenario	Sample mean	Sample bias	Sample standard deviation
1	0.2508317	0.0008316605	0.03108593
2	0.2525562	0.0025561947	0.02673882
3	0.2520031	0.0020031495	0.02511481
4	0.2543971	0.0043971271	0.03357481
5	0.2520360	0.0020359639	0.02870648
6	0.2525919	0.0025918701	0.02540891

Table 2: Simulation summaries for CV in the 6 scenarios with 50 persons in the study

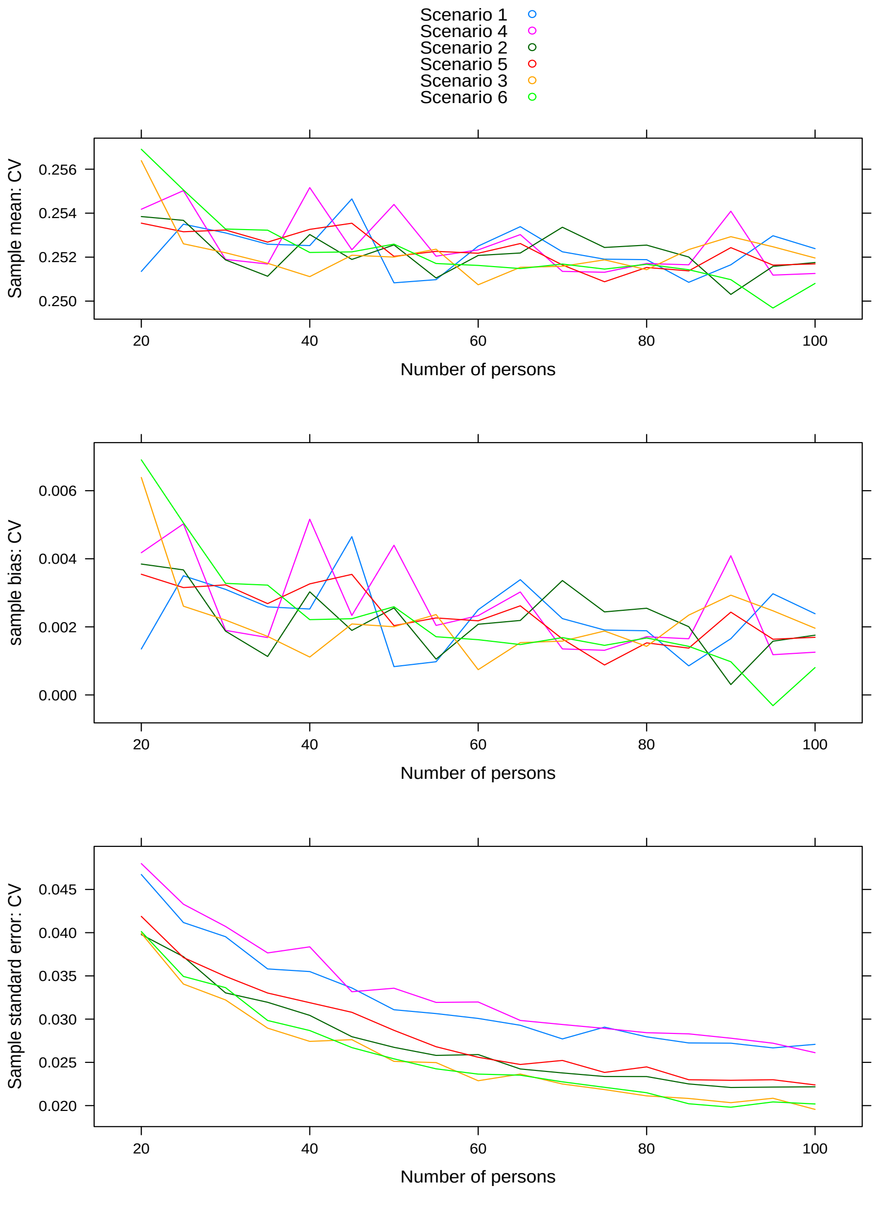

Figure 2: Simulation summaries for CV in the 6 scenarios as a function of number of persons in the study

5.3 Results for RMSE

Below we present a summary of the simulations with respect to $RMSE$

Scenario	Sample mean
1	28.33112
2	23.25717
3	21.07783
4	30.87935
5	27.08502
6	24.92916

Table 3: Sample mean of RMSE in the 6 scenarios with 50 persons in the study

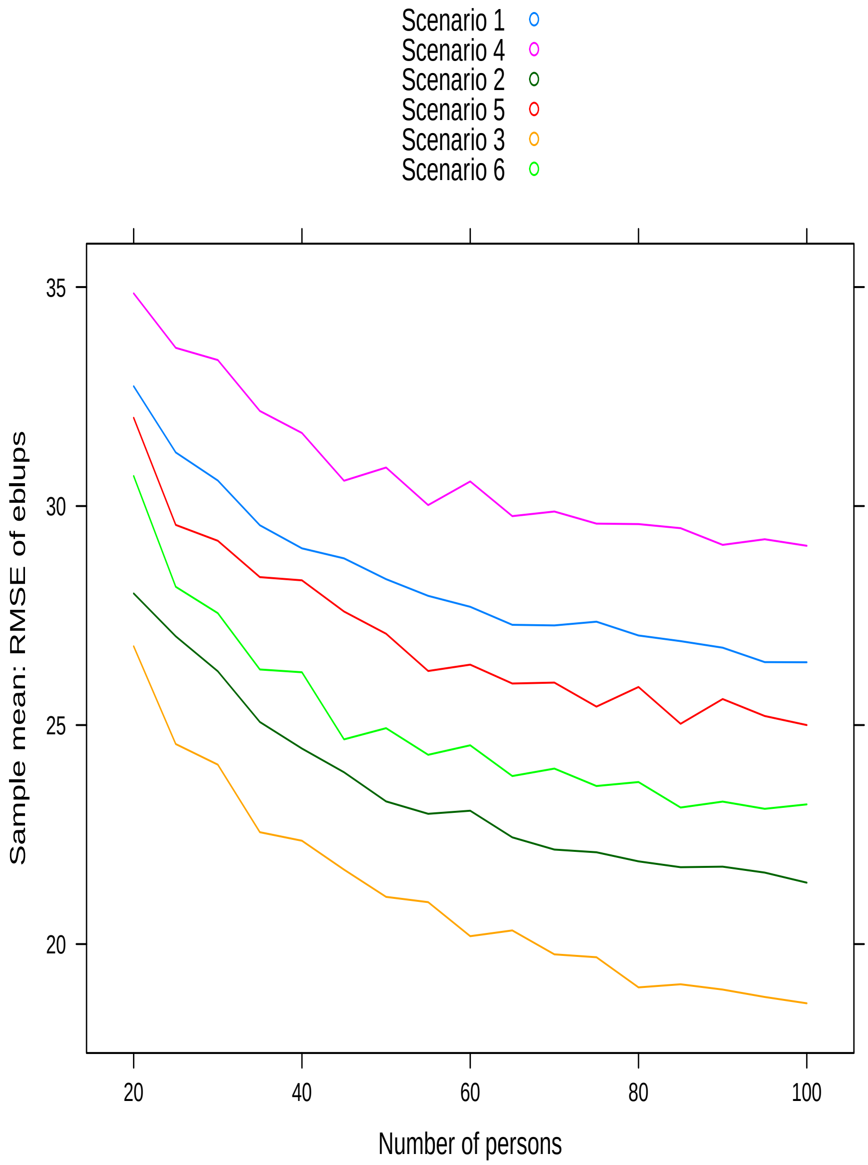

Figure 3: Sample mean of RMSE in the 6 scenarios as a function of number of persons in the study

6 Results: Simulation study 2

We considered the 8 scenarios:

- Scenario 1: $l = 2$, $m = 3$, $p = 0$. Corresponding to 2 observers, 3 trial days per person, and no missing observations
- Scenario 2: $l = 3$, $m = 3$, $p = 0$. Corresponding to 3 observers, 3 trial days per person, and no missing observations
- Scenario 3: $l = 2$, $m = 4$ and $p = 0$. Corresponding to 2 observers, 4 trial days per person, and no missing observations
- Scenario 4: $l = 3$, $m = 4$ and $p = 0$. Corresponding to 3 observers, 4 trial days per person, and no missing observations
- Scenario 5: $l = 2$, $m = 3$, $p = 0.1$. Corresponding to 2 observers, 3 trial days per person, and 10% of the persons in the trial with missing observations after trial day 1.
- Scenario 6: $l = 3$, $m = 3$, $p = 0.1$. Corresponding to 3 observers, 3 trial days per person, and 10% of the persons in the trial with missing observations after trial day 1.
- Scenario 7: $l = 2$, $m = 4$ and $p = 0.1$. Corresponding to 2 observers, 4 trial days per person, and 10% of the persons in the trial with missing observations after trial day 1.
- Scenario 8: $l = 3$, $m = 4$ and $p = 0.1$. Corresponding to 3 observers, 4 trial days per person, and and 10% of the persons in the trial with missing observations after trial day 1.

all with n ranging from 20 to 100 in steps of 5.

For each scenario and value of n 1000 data sets were generated. For each data set values of ICC, CV, and $RMSE$ were calculated as described above.

6.1 Results for ICC

Below we present a summary of the simulations with respect to ICC

Scenario	Sample mean	Sample bias	Sample standard deviation
1	0.7778314	-0.002813779	0.07210411
2	0.7756041	-0.005041078	0.06160949
3	0.7781486	-0.002496601	0.07179754
4	0.7759514	-0.004693713	0.05960432
5	0.7774007	-0.003244450	0.07275774
6	0.7758275	-0.004817703	0.06592454
7	0.7818980	0.001252878	0.06685797
8	0.7747113	-0.005933889	0.06352396

Table 1: Simulation summaries for ICC in the 8 scenarios with 50 persons in the study

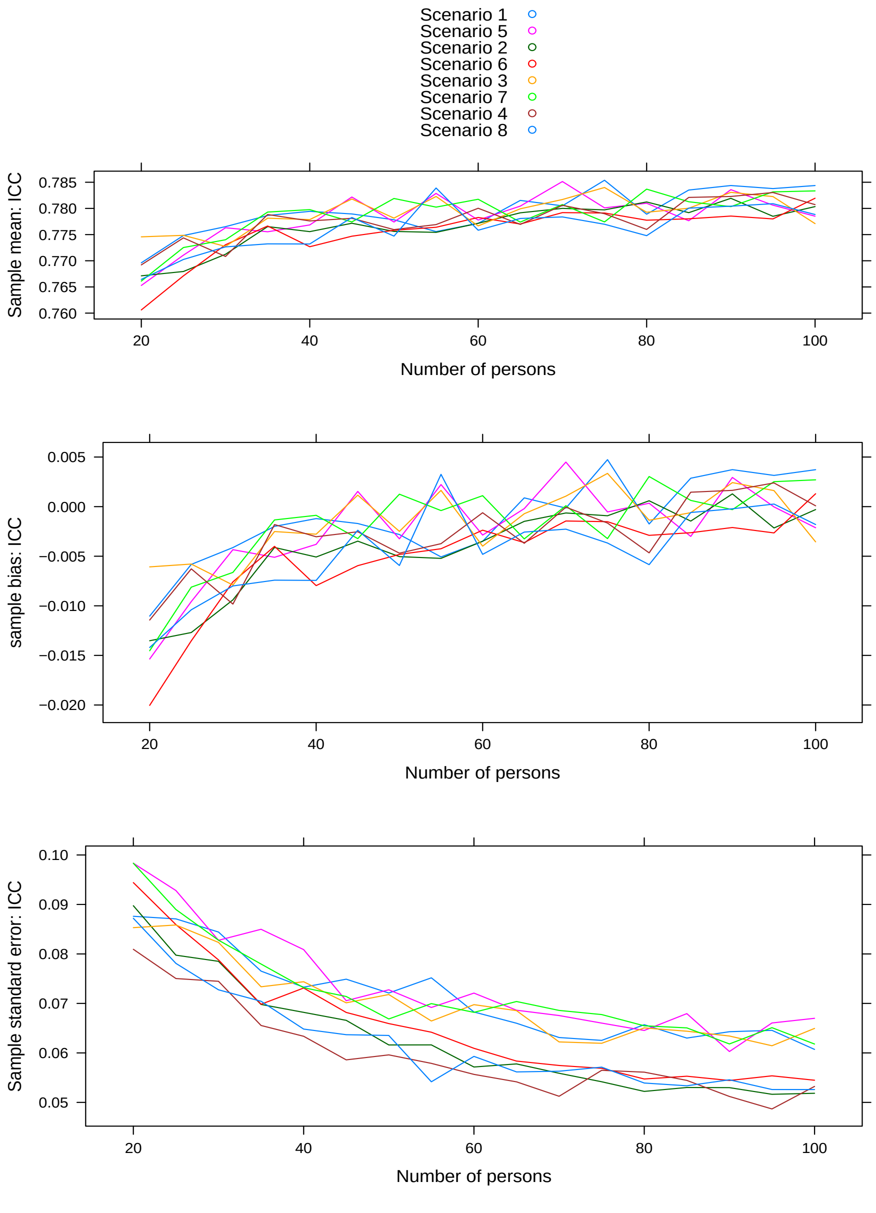

Figure 4: Simulation summaries for ICC in the 8 scenarios as a function of number of persons in the study

6.2 Results for CV

Below we present a summary of the simulations with respect to CV

Scenario	Sample mean	Sample bias	Sample standard deviation
1	0.2545871	0.004587095	0.04165318
2	0.2541242	0.004124157	0.03667416
3	0.2536367	0.003474176	0.03914850
4	0.2515846	0.003474176	0.03278173
5	0.2538751	0.003474176	0.04085610
6	0.2539972	0.003474176	0.03902607
7	0.2524710	0.002471031	0.03925632
8	0.2536266	0.003626568	0.03647348

Table 2: Simulation summaries for CV in the 8 scenarios with 50 persons in the study

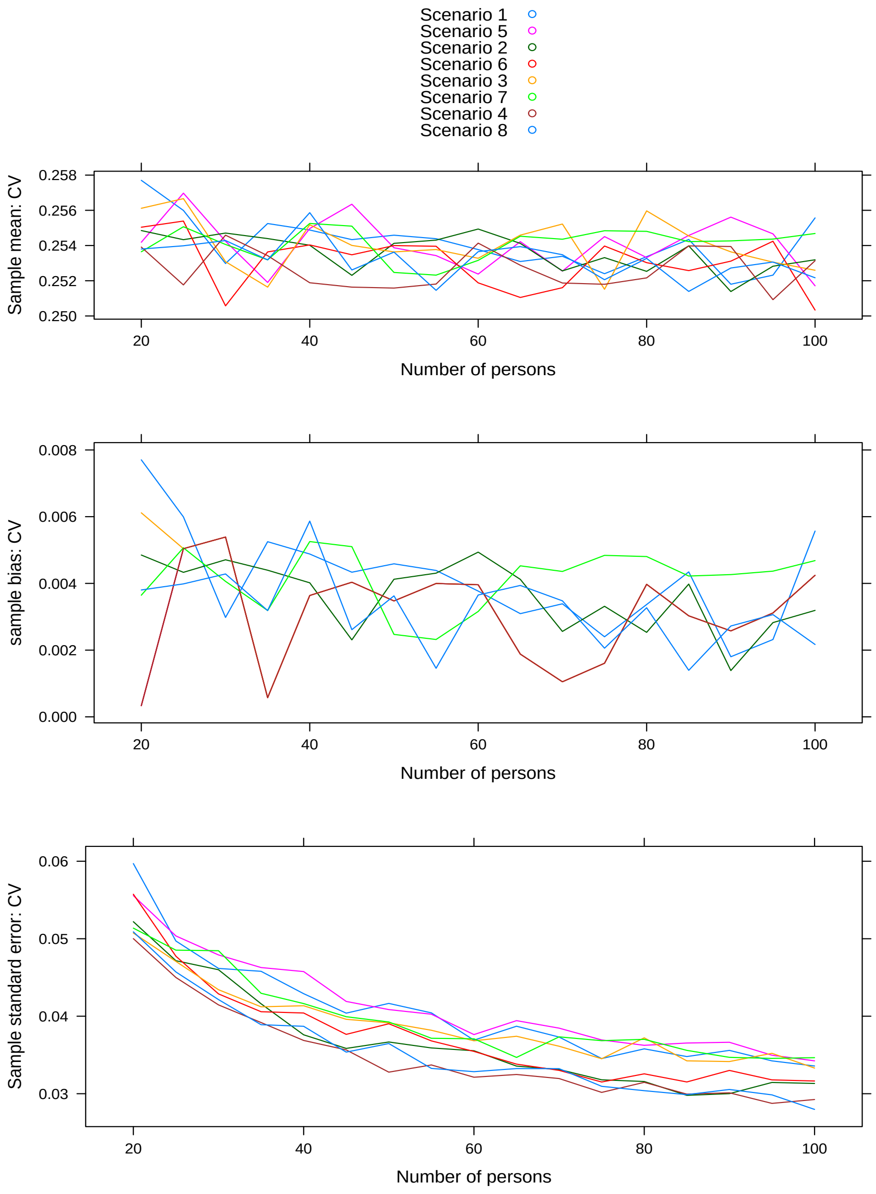

Figure 5: Simulation summaries for CV in the 8 scenarios as a function of number of persons in the study

6.3 Results for RMSE

Below we present a summary of the simulations with respect to $RMSE$

Scenario	Sample mean
1	31.59060
2	31.27784
3	27.98742
4	27.90517
5	33.24145
6	33.88289
7	31.03389
8	30.99810

Table 3: Sample mean of RMSE in the 6 scenarios with 50 persons in the study

7 Appendix: R code

```
#loading necesscary libraries
library(doBy)
library(lme4)

#############################
#Function for simulating bts#
#stimulation                #
#############################

#npers: number of persons in trial
#nobs: number of observers
#ntimepoints: number of days at which bts is conducted on a person
#meanhyperalg: average hyperalg area measurement with bts in population
#sd.obs: standard deviation of variance component for observer
#sd.pers: standard deviation of variance component for person
#sd.res: standard deviation of variance component for time within person
#missing.freq: Frequency of persons missing after first measurement

###################################################################
#Observers are randomly assigned to person and days so that
```

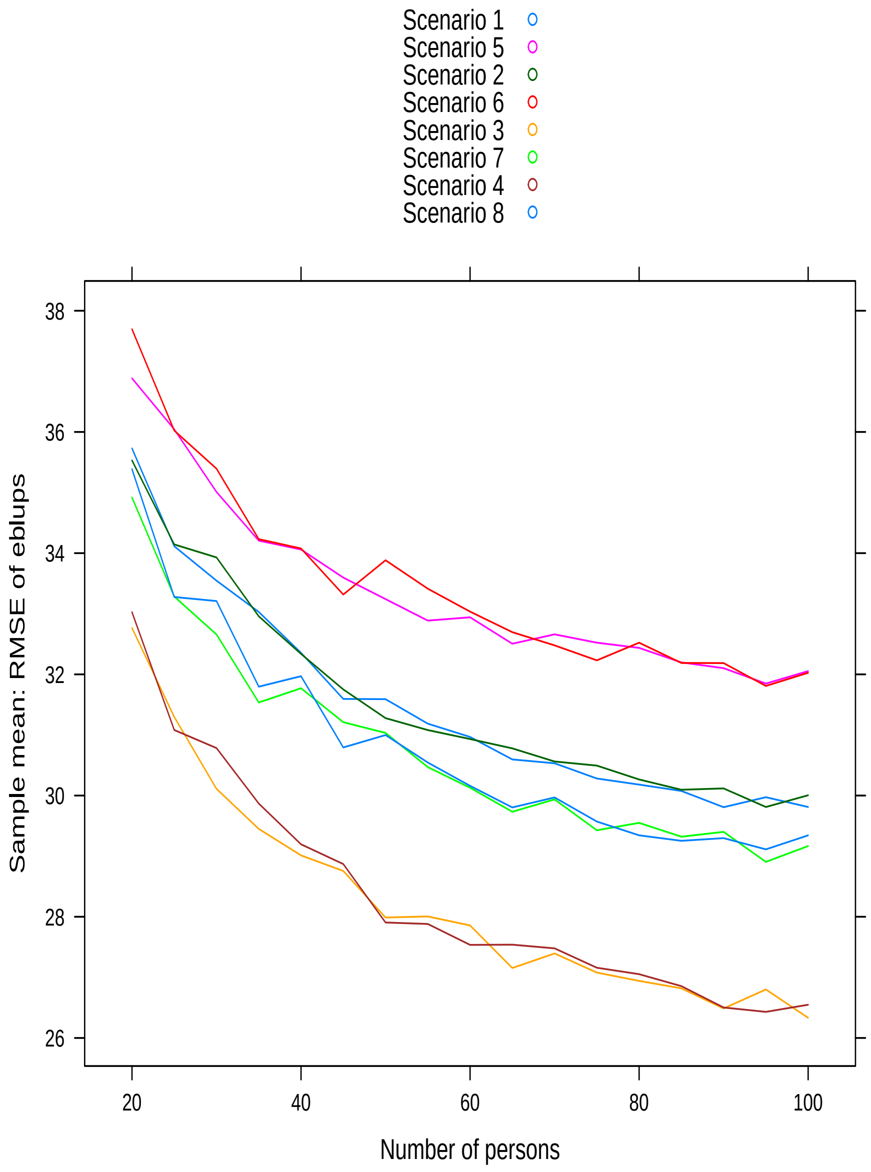

Figure 6: Sample mean of RMSE in the 6 scenarios as a function of number of persons in the study

```
#an observer will not observe the same
#person at two consequtive trial days
#################################################################

#################################################################
#For the fraction missing.freq of persons in the study with the
#largest measurements of bts on the first trial day no more
#measurements of hyperalg with bts are recorded
#################################################################


simulate.hyperalg<-function(npers,nobs,ntimepoints,meanhyperalg,sd.obs,sd.pers,sd.res,missing.freq)
  {
  observerid<-1:nobs
  observermat<-matrix(1,npers,ntimepoints)
  for (i in 1:npers){
    if (nobs>1){
    observermat[i,1]<-sample(nobs,1)
    }
    if (nobs==1){
      observermat[i,1]<-1}

    for (j in 2:ntimepoints){
      if (nobs>1){
      observermat[i,j]<-sample(subset(observerid,observerid!=observermat[i,j-1]),1)
      }
      if (nobs==1){
        observermat[i,j]<-1
      }
    }
}

  randobs<-rnorm(nobs,sd=sd.obs)
  randpers<-rnorm(npers,sd=sd.pers)
  observer<-as.vector(t(observermat))
  time<-rep(1:ntimepoints,npers)
  person<-as.vector(t(matrix(1:npers,npers,ntimepoints)))
  hyperalg.term<-rep(meanhyperalg,npers*ntimepoints)
  obs.term<-randobs[observer]
  pers.term<-randpers[person]
  res.term<-rnorm(npers*ntimepoints,sd=sd.res)
  response<-hyperalg.term+obs.term+pers.term+res.term
  hyperalgdata<-data.frame(person,observer,time,response,pers.term)

  nmiss<-floor(npers*missing.freq)

  tmpdata<-subset(hyperalgdata,time==1)[,c(1,4)]
  personorder<-orderBy(-response~person,data=tmpdata)[,1]
  missingorder<-c(rep(1,nmiss),rep(0,npers-nmiss))
  tmpdata1<-data.frame(personorder,missingorder)
  missing<-rep(orderBy(personorder~missingorder,data=tmpdata1)[,2],rep(ntimepoints,npers))
  response.missing<-hyperalgdata$response
  response.missing[missing==1 & hyperalgdata$time!=1]<-NA

  hyperalgdata$response.missing<-response.missing
  return(hyperalgdata)
}

############################################
```

```
#person sd, meanhyperalg,residual sd,and   #
#obs sd                                    #
#obtained from analysing metode.xls        #
#Extracting meanhyperalg as average        #
#at period 1                               #
############################################

origdata<-read.csv2(file.choose(),header=T)
day<-rep(rep(1:4,rep(3,4)),20)
period<-rep(1:3,4*20)
treatment<-rep(c(rep(1,3),rep(2,3),rep(3,3),rep(1,3)),20)
person<-as.vector(t(matrix(origdata$NR,20,12)))
tmpdata<-origdata[,c(2:4,6:8,10:12,14:16)]
bts<-rep(0,20*12)

for (i in 1:20){
  bts[((i-1)*12+1):(i*12)]<-as.vector(tmpdata[i,])
}
hyperalg<-unlist(bts)


anadata<-data.frame(hyperalg,day,treatment,period,person)

fit<-lmer(hyperalg~-1+factor(treatment):factor(period)+(1|person)+(1|person*day),data=anadata)
summary(fit)

###############
#Extractions#
#meanhyperalg=(183.43+232.84+204.16)/3~=207
#pers.sd~=121
#res.sd~=58
#obs.sd~=27 (assuming that there is a new observer for each
#             person at each day)

################
#intrapersoncorr<-14633.45/(736.18+14633.45+3384.48)
#intrapersoncorr
#[1] 0.7802796
#Coefficient of variation
#CV<-58/207
#0.2801932
##############################################
#Analysing GABA data                         #
##############################################

head(Gabadatacsv)

personid<-rep(1:25,2)
treatment<-c(rep(1,25),rep(2,25))
day<-with(Gabadatacsv,c(dag1,dag2))
hyperalg<-with(Gabadatacsv,c(area1,area2))

anadata2<-data.frame(personid,treatment,day,hyperalg)
orderBy(~personid,data=anadata2)

fit2<-lmer(hyperalg~-1+factor(day):factor(treatment)+(1|personid),
           data=anadata2)
summary(fit2)
###############
```

```
#Extractions#
#mean hyperalg~=195 (treatment=1, day 1)
#pers.sd~=99
#res.sd~=39

################
#intrapersoncorr<-9889.2/(9889.2+1514.9)
#intrapersoncorr
#[1] 0.8671618
#Coefficient of variation
#CV<-39/195
#0.2



############################
#Simulations          ######
############################

############################
#Setting parameters########

meanhyperalg<-200
sd.pers<-110
sd.res<-50
sd.obs<-30

##########################################################
#Simulation ranges from 20-100 persons                   #
#no missing at random                                    #
#summary statistics are based on 1000                    #
#simulated datasets for a given number of persons and    #
#with the above specifications                           #
##########################################################

numberpers<-rep(seq(20,100,by=5),6)
numberobservers<-rep(rep(c(4,7,10),rep(17,3)),2)
numbertimepoints<-rep(rep(c(4,7,10),rep(17,3)),2)
freqofmissing<-rep(c(0,0.1),c(51,51))
ICCbias<-rep(0,102)
ICCmean<-rep(0,102)
ICCsd<-rep(0,102)
CVbias<-rep(0,102)
CVmean<-rep(0,102)
CVsd<-rep(0,102)
sqrtMSEmean<-rep(0,102)

#Scenario 1
Nsim<-1000
npers<-seq(20,100,by=5)
ntimepoints<-4
nobs<-4
missing.freq<-0
intraperscorr<-matrix(0,length(npers),Nsim)
CV<-matrix(0,length(npers),Nsim)
MSEeblups<-matrix(0,length(npers),Nsim)

for (j in 1:length(npers)){
   for (i in 1:Nsim){
```

```
   anadata<-simulate.hyperalg(npers[j],nobs,ntimepoints,meanhyperalg,sd.obs,sd.pers,sd.res,missing.freq)
   fit<-lmer(response.missing~1+(1|observer)+(1|person),
            data=na.omit(anadata))
   variance<-as.numeric(summary(fit)@REmat[,3])
   meanval<-summary(fit)@coefs[1]
   intraperscorr[j,i]<-variance[1]/sum(variance)
   CV[j,i]<-sqrt(variance[3])/meanval
   eblups<-as.vector(unlist(ranef(fit)$person))
   MSEeblups[j,i]<-mean((eblups[anadata$person]-anadata$pers.term)^2)
   print(npers[j])
   print(i)


}
}



trueICC<-sd.pers^2/(sd.pers^2+sd.res^2+sd.obs^2)
ICCmean[1:17]<-apply(intraperscorr,1,mean)
ICCbias[1:17]<-ICCmean[1:17]-trueICC
ICCsd[1:17]<-apply(intraperscorr,1,sd)
trueCV<-sd.res/meanhyperalg
CVmean[1:17]<-apply(CV,1,mean)
CVbias[1:17]<-CVmean[1:17]-trueCV
CVsd[1:17]<-apply(CV,1,sd)
sqrtMSEmean[1:17]<-apply(sqrt(MSEeblups),1,mean)

#scenario 2

Nsim<-1000
npers<-seq(20,100,by=5)
ntimepoints<-7
nobs<-7
missing.freq<-0
intraperscorr<-matrix(0,length(npers),Nsim)
CV<-matrix(0,length(npers),Nsim)
MSEeblups<-matrix(0,length(npers),Nsim)

for (j in 1:length(npers)){
  for (i in 1:Nsim){
    anadata<-simulate.hyperalg(npers[j],nobs,ntimepoints,meanhyperalg,sd.obs,sd.pers,sd.res,missing.freq)
    fit<-lmer(response.missing~1+(1|observer)+(1|person),
             data=na.omit(anadata))
    variance<-as.numeric(summary(fit)@REmat[,3])
    meanval<-summary(fit)@coefs[1]
    intraperscorr[j,i]<-variance[1]/sum(variance)
    CV[j,i]<-sqrt(variance[3])/meanval
    eblups<-as.vector(unlist(ranef(fit)$person))
    MSEeblups[j,i]<-mean((eblups[anadata$person]-anadata$pers.term)^2)
    print(npers[j])
    print(i)


  }
}
```

```
trueICC<-sd.pers^2/(sd.pers^2+sd.res^2+sd.obs^2)
ICCmean[(17+1):(2*17)]<-apply(intraperscorr,1,mean)
ICCbias[(17+1):(2*17)]<-ICCmean[(17+1):(2*17)]-trueICC
ICCsd[(17+1):(2*17)]<-apply(intraperscorr,1,sd)
trueCV<-sd.res/meanhyperalg
CVmean[(17+1):(2*17)]<-apply(CV,1,mean)
CVbias[(17+1):(2*17)]<-CVmean[(17+1):(2*17)]-trueCV
CVsd[(17+1):(2*17)]<-apply(CV,1,sd)
sqrtMSEmean[(17+1):(2*17)]<-apply(sqrt(MSEeblups),1,mean)


#scenario 3

Nsim<-1000
npers<-seq(20,100,by=5)
ntimepoints<-10
nobs<-10
missing.freq<-0
intraperscorr<-matrix(0,length(npers),Nsim)
CV<-matrix(0,length(npers),Nsim)
MSEeblups<-matrix(0,length(npers),Nsim)

for (j in 1:length(npers)){
  for (i in 1:Nsim){
    anadata<-simulate.hyperalg(npers[j],nobs,ntimepoints,meanhyperalg,sd.obs,sd.pers,sd.res,missing.freq)
    fit<-lmer(response.missing~1+(1|observer)+(1|person),
              data=na.omit(anadata))
    variance<-as.numeric(summary(fit)@REmat[,3])
    meanval<-summary(fit)@coefs[1]
    intraperscorr[j,i]<-variance[1]/sum(variance)
    CV[j,i]<-sqrt(variance[3])/meanval
    eblups<-as.vector(unlist(ranef(fit)$person))
    MSEeblups[j,i]<-mean((eblups[anadata$person]-anadata$pers.term)^2)
    print(npers[j])
    print(i)


  }
}



trueICC<-sd.pers^2/(sd.pers^2+sd.res^2+sd.obs^2)
ICCmean[(2*17+1):(3*17)]<-apply(intraperscorr,1,mean)
ICCbias[(2*17+1):(3*17)]<-ICCmean[(2*17+1):(3*17)]-trueICC
ICCsd[(2*17+1):(3*17)]<-apply(intraperscorr,1,sd)
trueCV<-sd.res/meanhyperalg
CVmean[(2*17+1):(3*17)]<-apply(CV,1,mean)
CVbias[(2*17+1):(3*17)]<-CVmean[(2*17+1):(3*17)]-trueCV
CVsd[(2*17+1):(3*17)]<-apply(CV,1,sd)
sqrtMSEmean[(2*17+1):(3*17)]<-apply(sqrt(MSEeblups),1,mean)

#Scenario 4

Nsim<-1000
npers<-seq(20,100,by=5)
ntimepoints<-4
nobs<-4
missing.freq<-0.1
```

```
intraperscorr<-matrix(0,length(npers),Nsim)
CV<-matrix(0,length(npers),Nsim)
MSEeblups<-matrix(0,length(npers),Nsim)

for (j in 1:length(npers)){
  for (i in 1:Nsim){
    anadata<-simulate.hyperalg(npers[j],nobs,ntimepoints,meanhyperalg,sd.obs,sd.pers,sd.res,missing.freq)
    fit<-lmer(response.missing~1+(1|observer)+(1|person),
              data=na.omit(anadata))
    variance<-as.numeric(summary(fit)@REmat[,3])
    meanval<-summary(fit)@coefs[1]
    intraperscorr[j,i]<-variance[1]/sum(variance)
    CV[j,i]<-sqrt(variance[3])/meanval
    eblups<-as.vector(unlist(ranef(fit)$person))
    MSEeblups[j,i]<-mean((eblups[anadata$person]-anadata$pers.term)^2)
    print(npers[j])
    print(i)


  }
}



trueICC<-sd.pers^2/(sd.pers^2+sd.res^2+sd.obs^2)
ICCmean[(3*17+1):(4*17)]<-apply(intraperscorr,1,mean)
ICCbias[(3*17+1):(4*17)]<-ICCmean[(3*17+1):(4*17)]-trueICC
ICCsd[(3*17+1):(4*17)]<-apply(intraperscorr,1,sd)
trueCV<-sd.res/meanhyperalg
CVmean[(3*17+1):(4*17)]<-apply(CV,1,mean)
CVbias[(3*17+1):(4*17)]<-CVmean[(3*17+1):(4*17)]-trueCV
CVsd[(3*17+1):(4*17)]<-apply(CV,1,sd)
sqrtMSEmean[(3*17+1):(4*17)]<-apply(sqrt(MSEeblups),1,mean)



#Scenario 5

Nsim<-1000
npers<-seq(20,100,by=5)
ntimepoints<-7
nobs<-7
missing.freq<-0.1
intraperscorr<-matrix(0,length(npers),Nsim)
CV<-matrix(0,length(npers),Nsim)
MSEeblups<-matrix(0,length(npers),Nsim)

for (j in 1:length(npers)){
  for (i in 1:Nsim){
    anadata<-simulate.hyperalg(npers[j],nobs,ntimepoints,meanhyperalg,sd.obs,sd.pers,sd.res,missing.freq)
    fit<-lmer(response.missing~1+(1|observer)+(1|person),
              data=na.omit(anadata))
    variance<-as.numeric(summary(fit)@REmat[,3])
    meanval<-summary(fit)@coefs[1]
    intraperscorr[j,i]<-variance[1]/sum(variance)
    CV[j,i]<-sqrt(variance[3])/meanval
    eblups<-as.vector(unlist(ranef(fit)$person))
    MSEeblups[j,i]<-mean((eblups[anadata$person]-anadata$pers.term)^2)
    print(npers[j])
```

```
    print(i)


  }
}



trueICC<-sd.pers^2/(sd.pers^2+sd.res^2+sd.obs^2)
ICCmean[(4*17+1):(5*17)]<-apply(intraperscorr,1,mean)
ICCbias[(4*17+1):(5*17)]<-ICCmean[(4*17+1):(5*17)]-trueICC
ICCsd[(4*17+1):(5*17)]<-apply(intraperscorr,1,sd)
trueCV<-sd.res/meanhyperalg
CVmean[(4*17+1):(5*17)]<-apply(CV,1,mean)
CVbias[(4*17+1):(5*17)]<-CVmean[(4*17+1):(5*17)]-trueCV
CVsd[(4*17+1):(5*17)]<-apply(CV,1,sd)
sqrtMSEmean[(4*17+1):(5*17)]<-apply(sqrt(MSEeblups),1,mean)



#Scenario 6

Nsim<-1000
npers<-seq(20,100,by=5)
ntimepoints<-10
nobs<-10
missing.freq<-0.1
intraperscorr<-matrix(0,length(npers),Nsim)
CV<-matrix(0,length(npers),Nsim)
MSEeblups<-matrix(0,length(npers),Nsim)

for (j in 1:length(npers)){
  for (i in 1:Nsim){
    anadata<-simulate.hyperalg(npers[j],nobs,ntimepoints,meanhyperalg,sd.obs,sd.pers,sd.res,missing.freq)
    fit<-lmer(response.missing~1+(1|observer)+(1|person),
              data=na.omit(anadata))
    variance<-as.numeric(summary(fit)@REmat[,3])
    meanval<-summary(fit)@coefs[1]
    intraperscorr[j,i]<-variance[1]/sum(variance)
    CV[j,i]<-sqrt(variance[3])/meanval
    eblups<-as.vector(unlist(ranef(fit)$person))
    MSEeblups[j,i]<-mean((eblups[anadata$person]-anadata$pers.term)^2)
    print(npers[j])
    print(i)


  }
}



trueICC<-sd.pers^2/(sd.pers^2+sd.res^2+sd.obs^2)
ICCmean[(5*17+1):(6*17)]<-apply(intraperscorr,1,mean)
ICCbias[(5*17+1):(6*17)]<-ICCmean[(5*17+1):(6*17)]-trueICC
ICCsd[(5*17+1):(6*17)]<-apply(intraperscorr,1,sd)
trueCV<-sd.res/meanhyperalg
CVmean[(5*17+1):(6*17)]<-apply(CV,1,mean)
CVbias[(5*17+1):(6*17)]<-CVmean[(5*17+1):(6*17)]-trueCV
CVsd[(5*17+1):(6*17)]<-apply(CV,1,sd)
```

```
sqrtMSEmean[(5*17+1):(6*17)]<-apply(sqrt(MSEeblups),1,mean)


#Collecting and saving simulation results

simresults<-data.frame(numberpers,numberobservers,numbertimepoints,
                       freqofmissing,ICCmean,ICCbias,ICCsd,CVmean,CVbias,
                       CVsd,sqrtMSEmean)
write.table(simresults, file.choose(),quote=F)

library(lattice)

group<-with(simresults,factor(numbertimepoints):factor(freqofmissing))
levels(group)<-c("Scenario 1","Scenario 4",
                 "Scenario 2","Scenario 5",
                 "Scenario 3","Scenario 6")

simresults$group<-group


#Plots of ICC

plot1<-xyplot(ICCmean~numberpers,groups=group,key=simpleKey(levels(simresults$group)),type="l",
       data=simresults,ylab="Sample mean: ICC",xlab="Number of persons")

plot2<-xyplot(ICCbias~numberpers,groups=group,type="l",
       data=simresults,ylab="sample bias: ICC",xlab="Number of persons")

plot3<-xyplot(ICCsd~numberpers,groups=group,type="l",
       data=simresults,ylab="Sample standard error: ICC",xlab="Number of persons")

dev.off()
print(plot1,split=c(1,1,1,3),more=TRUE)
print(plot2,split=c(1,2,1,3),more=TRUE)
print(plot3,split=c(1,3,1,3))

#Plots of CV

plot1<-xyplot(CVmean~numberpers,groups=group,key=simpleKey(levels(simresults$group)),type="l",
       data=simresults,ylab="Sample mean: CV",xlab="Number of persons")

plot2<-xyplot(CVbias~numberpers,groups=group,type="l",
       data=simresults,ylab="sample bias: CV",xlab="Number of persons")

plot3<-xyplot(CVsd~numberpers,groups=group,type="l",
       data=simresults,ylab="Sample standard error: CV",xlab="Number of persons")
dev.off()
print(plot1,split=c(1,1,1,3),more=TRUE)
print(plot2,split=c(1,2,1,3),more=TRUE)
print(plot3,split=c(1,3,1,3))


#Plots of RMSE-eblups

xyplot(sqrtMSEmean~numberpers,groups=group,key=simpleKey(levels(simresults$group)),type="l",
       data=simresults,ylab="Sample mean: RMSE of eblups",xlab="Number of persons")


##############################
```

```
#Numbers for 50 person sample#
##############################

simresults50<-subset(simresults,numberpers==50)
simresults50

################################
numberpers numberobservers numbertimepoints freqofmissing   ICCmean      ICCbias      ICCsd
7          50              4                4           0.0 0.7794726 -0.001172594 0.05303596
24         50              7                7           0.0 0.7756883 -0.004956840 0.04645277
41         50             10               10           0.0 0.7781922 -0.002452985 0.04341995
58         50              4                4           0.1 0.7773741 -0.003271055 0.05780629
75         50              7                7           0.1 0.7724449 -0.008200232 0.04868458
92         50             10               10           0.1 0.7739216 -0.006723540 0.04733749
CVmean       CVbias       CVsd sqrtMSEmean      group
7  0.2508317 0.0008316605 0.03108593    28.33112 Scenario 1
24 0.2525562 0.0025561947 0.02673882    23.25717 Scenario 2
41 0.2520031 0.0020031495 0.02511481    21.07783 Scenario 3
58 0.2543971 0.0043971271 0.03357481    30.87935 Scenario 4
75 0.2520360 0.0020359639 0.02870648    27.08502 Scenario 5
92 0.2525919 0.0025918701 0.02540891    24.92916 Scenario 6
####################################


#########################################################
#New simulations: spec of number of observers and visits #
#Simulation ranges from 20-100 persons                  #
#no missing at random                                   #
#summary statistics are based on 1000                   #
#simulated datasets for a given number of persons and   #
#with the above specifications                          #
#########################################################

numberpers<-rep(seq(20,100,by=5),8)
numberobservers<-rep(rep(c(2,3),rep(17,2)),4)
numbertimepoints<-rep(rep(c(3,4),rep(34,2)),2)
freqofmissing<-rep(c(0,0.1),c(68,68))
ICCbias<-rep(0,2*68)
ICCmean<-rep(0,2*68)
ICCsd<-rep(0,2*68)
CVbias<-rep(0,2*68)
CVmean<-rep(0,2*68)
CVsd<-rep(0,2*68)
sqrtMSEmean<-rep(0,2*68)

#Scenario 7
Nsim<-1000
npers<-seq(20,100,by=5)
ntimepoints<-3
nobs<-2
missing.freq<-0
intraperscorr<-matrix(0,length(npers),Nsim)
CV<-matrix(0,length(npers),Nsim)
MSEeblups<-matrix(0,length(npers),Nsim)

for (j in 1:length(npers)){
  for (i in 1:Nsim){
    anadata<-simulate.hyperalg(npers[j],nobs,ntimepoints,meanhyperalg,sd.obs,sd.pers,sd.res,missing.freq)
    fit<-lmer(response.missing~1+(1|observer)+(1|person),
```

```
              data=na.omit(anadata))
    variance<-as.numeric(summary(fit)@REmat[,3])
    meanval<-summary(fit)@coefs[1]
    intraperscorr[j,i]<-variance[1]/sum(variance)
    CV[j,i]<-sqrt(variance[3])/meanval
    eblups<-as.vector(unlist(ranef(fit)$person))
    MSEeblups[j,i]<-mean((eblups[anadata$person]-anadata$pers.term)^2)
    print(npers[j])
    print(i)


  }
}




trueICC<-sd.pers^2/(sd.pers^2+sd.res^2+sd.obs^2)
ICCmean[1:17]<-apply(intraperscorr,1,mean)
ICCbias[1:17]<-ICCmean[1:17]-trueICC
ICCsd[1:17]<-apply(intraperscorr,1,sd)
trueCV<-sd.res/meanhyperalg
CVmean[1:17]<-apply(CV,1,mean)
CVbias[1:17]<-CVmean[1:17]-trueCV
CVsd[1:17]<-apply(CV,1,sd)
sqrtMSEmean[1:17]<-apply(sqrt(MSEeblups),1,mean)

#scenario 8

Nsim<-1000
npers<-seq(20,100,by=5)
ntimepoints<-3
nobs<-3
missing.freq<-0
intraperscorr<-matrix(0,length(npers),Nsim)
CV<-matrix(0,length(npers),Nsim)
MSEeblups<-matrix(0,length(npers),Nsim)

for (j in 1:length(npers)){
  for (i in 1:Nsim){
    anadata<-simulate.hyperalg(npers[j],nobs,ntimepoints,meanhyperalg,sd.obs,sd.pers,sd.res,missing.freq)
    fit<-lmer(response.missing~1+(1|observer)+(1|person),
              data=na.omit(anadata))
    variance<-as.numeric(summary(fit)@REmat[,3])
    meanval<-summary(fit)@coefs[1]
    intraperscorr[j,i]<-variance[1]/sum(variance)
    CV[j,i]<-sqrt(variance[3])/meanval
    eblups<-as.vector(unlist(ranef(fit)$person))
    MSEeblups[j,i]<-mean((eblups[anadata$person]-anadata$pers.term)^2)
    print(npers[j])
    print(i)


  }
}




trueICC<-sd.pers^2/(sd.pers^2+sd.res^2+sd.obs^2)
ICCmean[(17+1):(2*17)]<-apply(intraperscorr,1,mean)
```

```
ICCbias[(17+1):(2*17)]<-ICCmean[(17+1):(2*17)]-trueICC
ICCsd[(17+1):(2*17)]<-apply(intraperscorr,1,sd)
trueCV<-sd.res/meanhyperalg
CVmean[(17+1):(2*17)]<-apply(CV,1,mean)
CVbias[(17+1):(2*17)]<-CVmean[(17+1):(2*17)]-trueCV
CVsd[(17+1):(2*17)]<-apply(CV,1,sd)
sqrtMSEmean[(17+1):(2*17)]<-apply(sqrt(MSEeblups),1,mean)


#scenario 9

Nsim<-1000
npers<-seq(20,100,by=5)
ntimepoints<-4
nobs<-2
missing.freq<-0
intraperscorr<-matrix(0,length(npers),Nsim)
CV<-matrix(0,length(npers),Nsim)
MSEeblups<-matrix(0,length(npers),Nsim)

for (j in 1:length(npers)){
  for (i in 1:Nsim){
    anadata<-simulate.hyperalg(npers[j],nobs,ntimepoints,meanhyperalg,sd.obs,sd.pers,sd.res,missing.freq)
    fit<-lmer(response.missing~1+(1|observer)+(1|person),
              data=na.omit(anadata))
    variance<-as.numeric(summary(fit)@REmat[,3])
    meanval<-summary(fit)@coefs[1]
    intraperscorr[j,i]<-variance[1]/sum(variance)
    CV[j,i]<-sqrt(variance[3])/meanval
    eblups<-as.vector(unlist(ranef(fit)$person))
    MSEeblups[j,i]<-mean((eblups[anadata$person]-anadata$pers.term)^2)
    print(npers[j])
    print(i)


  }
}



trueICC<-sd.pers^2/(sd.pers^2+sd.res^2+sd.obs^2)
ICCmean[(2*17+1):(3*17)]<-apply(intraperscorr,1,mean)
ICCbias[(2*17+1):(3*17)]<-ICCmean[(2*17+1):(3*17)]-trueICC
ICCsd[(2*17+1):(3*17)]<-apply(intraperscorr,1,sd)
trueCV<-sd.res/meanhyperalg
CVmean[(2*17+1):(3*17)]<-apply(CV,1,mean)
CVbias[(2*17+1):(3*17)]<-CVmean[(2*17+1):(3*17)]-trueCV
CVsd[(2*17+1):(3*17)]<-apply(CV,1,sd)
sqrtMSEmean[(2*17+1):(3*17)]<-apply(sqrt(MSEeblups),1,mean)

#Scenario 10

Nsim<-1000
npers<-seq(20,100,by=5)
ntimepoints<-4
nobs<-3
missing.freq<-0
intraperscorr<-matrix(0,length(npers),Nsim)
CV<-matrix(0,length(npers),Nsim)
```

```
MSEeblups<-matrix(0,length(npers),Nsim)

for (j in 1:length(npers)){
  for (i in 1:Nsim){
    anadata<-simulate.hyperalg(npers[j],nobs,ntimepoints,meanhyperalg,sd.obs,sd.pers,sd.res,missing.freq)
    fit<-lmer(response.missing~1+(1|observer)+(1|person),
              data=na.omit(anadata))
    variance<-as.numeric(summary(fit)@REmat[,3])
    meanval<-summary(fit)@coefs[1]
    intraperscorr[j,i]<-variance[1]/sum(variance)
    CV[j,i]<-sqrt(variance[3])/meanval
    eblups<-as.vector(unlist(ranef(fit)$person))
    MSEeblups[j,i]<-mean((eblups[anadata$person]-anadata$pers.term)^2)
    print(npers[j])
    print(i)


  }
}



trueICC<-sd.pers^2/(sd.pers^2+sd.res^2+sd.obs^2)
ICCmean[(3*17+1):(4*17)]<-apply(intraperscorr,1,mean)
ICCbias[(3*17+1):(4*17)]<-ICCmean[(3*17+1):(4*17)]-trueICC
ICCsd[(3*17+1):(4*17)]<-apply(intraperscorr,1,sd)
trueCV<-sd.res/meanhyperalg
CVmean[(3*17+1):(4*17)]<-apply(CV,1,mean)
CVbias[(3*17+1):(4*17)]<-CVmean[(3*17+1):(4*17)]-trueCV
CVsd[(3*17+1):(4*17)]<-apply(CV,1,sd)
sqrtMSEmean[(3*17+1):(4*17)]<-apply(sqrt(MSEeblups),1,mean)


#Scenario 11
Nsim<-1000
npers<-seq(20,100,by=5)
ntimepoints<-3
nobs<-2
missing.freq<-0.1
intraperscorr<-matrix(0,length(npers),Nsim)
CV<-matrix(0,length(npers),Nsim)
MSEeblups<-matrix(0,length(npers),Nsim)

for (j in 1:length(npers)){
  for (i in 1:Nsim){
    anadata<-simulate.hyperalg(npers[j],nobs,ntimepoints,meanhyperalg,sd.obs,sd.pers,sd.res,missing.freq)
    fit<-lmer(response.missing~1+(1|observer)+(1|person),
              data=na.omit(anadata))
    variance<-as.numeric(summary(fit)@REmat[,3])
    meanval<-summary(fit)@coefs[1]
    intraperscorr[j,i]<-variance[1]/sum(variance)
    CV[j,i]<-sqrt(variance[3])/meanval
    eblups<-as.vector(unlist(ranef(fit)$person))
    MSEeblups[j,i]<-mean((eblups[anadata$person]-anadata$pers.term)^2)
    print(npers[j])
    print(i)


  }
```

```
}


trueICC<-sd.pers^2/(sd.pers^2+sd.res^2+sd.obs^2)
ICCmean[(4*17+1):(5*17)]<-apply(intraperscorr,1,mean)
ICCbias[(4*17+1):(5*17)]<-ICCmean[(4*17+1):(5*17)]-trueICC
ICCsd[(4*17+1):(5*17)]<-apply(intraperscorr,1,sd)
trueCV<-sd.res/meanhyperalg
CVmean[(4*17+1):(5*17)]<-apply(CV,1,mean)
CVbias[(4*17+1):(5*17)]<-CVmean[(4*17+1):(5*17)]-trueCV
CVsd[(4*17+1):(5*17)]<-apply(CV,1,sd)
sqrtMSEmean[(4*17+1):(5*17)]<-apply(sqrt(MSEeblups),1,mean)


#scenario 12

Nsim<-1000
npers<-seq(20,100,by=5)
ntimepoints<-3
nobs<-3
missing.freq<-0.1
intraperscorr<-matrix(0,length(npers),Nsim)
CV<-matrix(0,length(npers),Nsim)
MSEeblups<-matrix(0,length(npers),Nsim)

for (j in 1:length(npers)){
  for (i in 1:Nsim){
    anadata<-simulate.hyperalg(npers[j],nobs,ntimepoints,meanhyperalg,sd.obs,sd.pers,sd.res,missing.freq)
    fit<-lmer(response.missing~1+(1|observer)+(1|person),
              data=na.omit(anadata))
    variance<-as.numeric(summary(fit)@REmat[,3])
    meanval<-summary(fit)@coefs[1]
    intraperscorr[j,i]<-variance[1]/sum(variance)
    CV[j,i]<-sqrt(variance[3])/meanval
    eblups<-as.vector(unlist(ranef(fit)$person))
    MSEeblups[j,i]<-mean((eblups[anadata$person]-anadata$pers.term)^2)
    print(npers[j])
    print(i)


  }
}


trueICC<-sd.pers^2/(sd.pers^2+sd.res^2+sd.obs^2)
ICCmean[(5*17+1):(6*17)]<-apply(intraperscorr,1,mean)
ICCbias[(5*17+1):(6*17)]<-ICCmean[(5*17+1):(6*17)]-trueICC
ICCsd[(5*17+1):(6*17)]<-apply(intraperscorr,1,sd)
trueCV<-sd.res/meanhyperalg
CVmean[(5*17+1):(6*17)]<-apply(CV,1,mean)
CVbias[(5*7+1):(6*17)]<-CVmean[(5*17+1):(6*17)]-trueCV
CVsd[(5*17+1):(6*17)]<-apply(CV,1,sd)
sqrtMSEmean[(5*17+1):(6*17)]<-apply(sqrt(MSEeblups),1,mean)


#scenario 13

Nsim<-1000
```

```
npers<-seq(20,100,by=5)
ntimepoints<-4
nobs<-2
missing.freq<-0.1
intraperscorr<-matrix(0,length(npers),Nsim)
CV<-matrix(0,length(npers),Nsim)
MSEeblups<-matrix(0,length(npers),Nsim)

for (j in 1:length(npers)){
  for (i in 1:Nsim){
    anadata<-simulate.hyperalg(npers[j],nobs,ntimepoints,meanhyperalg,sd.obs,sd.pers,sd.res,missing.freq)
    fit<-lmer(response.missing~1+(1|observer)+(1|person),
              data=na.omit(anadata))
    variance<-as.numeric(summary(fit)@REmat[,3])
    meanval<-summary(fit)@coefs[1]
    intraperscorr[j,i]<-variance[1]/sum(variance)
    CV[j,i]<-sqrt(variance[3])/meanval
    eblups<-as.vector(unlist(ranef(fit)$person))
    MSEeblups[j,i]<-mean((eblups[anadata$person]-anadata$pers.term)^2)
    print(npers[j])
    print(i)


  }
}



trueICC<-sd.pers^2/(sd.pers^2+sd.res^2+sd.obs^2)
ICCmean[(6*17+1):(7*17)]<-apply(intraperscorr,1,mean)
ICCbias[(6*17+1):(7*17)]<-ICCmean[(6*17+1):(7*17)]-trueICC
ICCsd[(6*17+1):(7*17)]<-apply(intraperscorr,1,sd)
trueCV<-sd.res/meanhyperalg
CVmean[(6*17+1):(7*17)]<-apply(CV,1,mean)
CVbias[(6*17+1):(7*17)]<-CVmean[(6*17+1):(7*17)]-trueCV
CVsd[(6*17+1):(7*17)]<-apply(CV,1,sd)
sqrtMSEmean[(6*17+1):(7*17)]<-apply(sqrt(MSEeblups),1,mean)

#Scenario 14

Nsim<-1000
npers<-seq(20,100,by=5)
ntimepoints<-4
nobs<-3
missing.freq<-0.1
intraperscorr<-matrix(0,length(npers),Nsim)
CV<-matrix(0,length(npers),Nsim)
MSEeblups<-matrix(0,length(npers),Nsim)

for (j in 1:length(npers)){
  for (i in 1:Nsim){
    anadata<-simulate.hyperalg(npers[j],nobs,ntimepoints,meanhyperalg,sd.obs,sd.pers,sd.res,missing.freq)
    fit<-lmer(response.missing~1+(1|observer)+(1|person),
              data=na.omit(anadata))
    variance<-as.numeric(summary(fit)@REmat[,3])
    meanval<-summary(fit)@coefs[1]
    intraperscorr[j,i]<-variance[1]/sum(variance)
    CV[j,i]<-sqrt(variance[3])/meanval
    eblups<-as.vector(unlist(ranef(fit)$person))
```

```
    MSEeblups[j,i]<-mean((eblups[anadata$person]-anadata$pers.term)^2)
    print(npers[j])
    print(i)


  }
}




trueICC<-sd.pers^2/(sd.pers^2+sd.res^2+sd.obs^2)
ICCmean[(7*17+1):(8*17)]<-apply(intraperscorr,1,mean)
ICCbias[(7*17+1):(8*17)]<-ICCmean[(7*17+1):(8*17)]-trueICC
ICCsd[(7*17+1):(8*17)]<-apply(intraperscorr,1,sd)
trueCV<-sd.res/meanhyperalg
CVmean[(7*17+1):(8*17)]<-apply(CV,1,mean)
CVbias[(7*17+1):(8*17)]<-CVmean[(7*17+1):(8*17)]-trueCV
CVsd[(7*17+1):(8*17)]<-apply(CV,1,sd)
sqrtMSEmean[(7*17+1):(8*17)]<-apply(sqrt(MSEeblups),1,mean)


#Collecting and saving simulation results

simresults<-data.frame(numberpers,numberobservers,numbertimepoints,
                       freqofmissing,ICCmean,ICCbias,ICCsd,CVmean,CVbias,
                       CVsd,sqrtMSEmean)
write.table(simresults, file.choose(),quote=F)

library(lattice)

group<-with(simresults,factor(numbertimepoints):factor(numberobservers):factor(freqofmissing))
levels(group)<-c("Scenario 1","Scenario 5",
                 "Scenario 2","Scenario 6",
                 "Scenario 3","Scenario 7",
                 "Scenario 4","Scenario 8")

simresults$group<-group


#Plots of ICC

plot1<-xyplot(ICCmean~numberpers,groups=group,key=simpleKey(levels(simresults$group)),type="l",
              data=simresults,ylab="Sample mean: ICC",xlab="Number of persons")

plot2<-xyplot(ICCbias~numberpers,groups=group,type="l",
              data=simresults,ylab="sample bias: ICC",xlab="Number of persons")

plot3<-xyplot(ICCsd~numberpers,groups=group,type="l",
              data=simresults,ylab="Sample standard error: ICC",xlab="Number of persons")

dev.off()
print(plot1,split=c(1,1,1,3),more=TRUE)
print(plot2,split=c(1,2,1,3),more=TRUE)
print(plot3,split=c(1,3,1,3))

#Plots of CV

plot1<-xyplot(CVmean~numberpers,groups=group,key=simpleKey(levels(simresults$group)),type="l",
              data=simresults,ylab="Sample mean: CV",xlab="Number of persons")
```

```
plot2<-xyplot(CVbias~numberpers,groups=group,type="l",
              data=simresults,ylab="sample bias: CV",xlab="Number of persons")

plot3<-xyplot(CVsd~numberpers,groups=group,type="l",
              data=simresults,ylab="Sample standard error: CV",xlab="Number of persons")
dev.off()
print(plot1,split=c(1,1,1,3),more=TRUE)
print(plot2,split=c(1,2,1,3),more=TRUE)
print(plot3,split=c(1,3,1,3))


#Plots of RMSE-eblups

xyplot(sqrtMSEmean~numberpers,groups=group,key=simpleKey(levels(simresults$group)),type="l",
       data=simresults,ylab="Sample mean: RMSE of eblups",xlab="Number of persons")

simresults50<-subset(simresults,numberpers==50)



simresults50


#########################################################

> simresults50
numberpers numberobservers numbertimepoints freqofmissing
7           50              2               3           0.0
24          50              3               3           0.0
41          50              2               4           0.0
58          50              3               4           0.0
75          50              2               3           0.1
92          50              3               3           0.1
109         50              2               4           0.1
126         50              3               4           0.1
ICCmean      ICCbias      ICCsd    CVmean      CVbias
7   0.7778314 -0.002813779 0.07210411 0.2545871 0.004587095
24  0.7756041 -0.005041078 0.06160949 0.2541242 0.004124157
41  0.7781486 -0.002496601 0.07179754 0.2536367 0.003474176
58  0.7759514 -0.004693713 0.05960432 0.2515846 0.003474176
75  0.7774007 -0.003244450 0.07275774 0.2538751 0.003474176
92  0.7758275 -0.004817703 0.06592454 0.2539972 0.003474176
109 0.7818980  0.001252878 0.06685797 0.2524710 0.002471031
126 0.7747113 -0.005933889 0.06352396 0.2536266 0.003626568
CVsd sqrtMSEmean      group
7   0.04165318    31.59060 Scenario 1
24  0.03667416    31.27784 Scenario 2
41  0.03914850    27.98742 Scenario 3
58  0.03278173    27.90517 Scenario 4
75  0.04085610    33.24145 Scenario 5
92  0.03902607    33.88289 Scenario 6
109 0.03925632    31.03389 Scenario 7
126 0.03647348    30.99810 Scenario 8

#############################################################

##########################################################
#New simulations: spec  observer=1,visits=4              #
```

```
#Simulation ranges from 20-100 persons                  #
#no missing at random                                   #
#summary statistics are based on 1000                   #
#simulated datasets for a given number of persons and   #
#with the above specifications                          #
##########################################################

numberpers<-rep(seq(20,100,by=5),2)
numberobservers<-rep(1,34)
numbertimepoints<-rep(4,34)
freqofmissing<-rep(c(0,0.1),c(17,17))
ICCbias<-rep(0,2*17)
ICCmean<-rep(0,2*17)
ICCsd<-rep(0,2*17)
CVbias<-rep(0,2*17)
CVmean<-rep(0,2*17)
CVsd<-rep(0,2*17)
sqrtMSEmean<-rep(0,2*17)

#Scenario 15
Nsim<-1000
npers<-seq(20,100,by=5)
ntimepoints<-4
nobs<-1
missing.freq<-0
intraperscorr<-matrix(0,length(npers),Nsim)
CV<-matrix(0,length(npers),Nsim)
MSEeblups<-matrix(0,length(npers),Nsim)

for (j in 1:length(npers)){
  for (i in 1:Nsim){
    anadata<-simulate.hyperalg(npers[j],nobs,ntimepoints,meanhyperalg,sd.obs,sd.pers,sd.res,missing.freq)
    fit<-lmer(response.missing~1+(1|person),
              data=na.omit(anadata))
    variance<-as.numeric(summary(fit)@REmat[,3])
    meanval<-summary(fit)@coefs[1]
    intraperscorr[j,i]<-variance[1]/sum(variance)
    CV[j,i]<-sqrt(variance[2])/meanval
    eblups<-as.vector(unlist(ranef(fit)$person))
    MSEeblups[j,i]<-mean((eblups[anadata$person]-anadata$pers.term)^2)
    print(npers[j])
    print(i)


  }
}



trueICC<-sd.pers^2/(sd.pers^2+sd.res^2)
ICCmean[1:17]<-apply(intraperscorr,1,mean)
ICCbias[1:17]<-ICCmean[1:17]-trueICC
ICCsd[1:17]<-apply(intraperscorr,1,sd)
trueCV<-sd.res/meanhyperalg
CVmean[1:17]<-apply(CV,1,mean)
CVbias[1:17]<-CVmean[1:17]-trueCV
CVsd[1:17]<-apply(CV,1,sd)
sqrtMSEmean[1:17]<-apply(sqrt(MSEeblups),1,mean)
```

```
#scenario 16

Nsim<-1000
npers<-seq(20,100,by=5)
ntimepoints<-4
nobs<-1
missing.freq<-0.1
intraperscorr<-matrix(0,length(npers),Nsim)
CV<-matrix(0,length(npers),Nsim)
MSEeblups<-matrix(0,length(npers),Nsim)

for (j in 1:length(npers)){
  for (i in 1:Nsim){
    anadata<-simulate.hyperalg(npers[j],nobs,ntimepoints,meanhyperalg,sd.obs,sd.pers,sd.res,missing.freq)
    fit<-lmer(response.missing~1+(1|person),
              data=na.omit(anadata))
    variance<-as.numeric(summary(fit)@REmat[,3])
    meanval<-summary(fit)@coefs[1]
    intraperscorr[j,i]<-variance[1]/sum(variance)
    CV[j,i]<-sqrt(variance[2])/meanval
    eblups<-as.vector(unlist(ranef(fit)$person))
    MSEeblups[j,i]<-mean((eblups[anadata$person]-anadata$pers.term)^2)
    print(npers[j])
    print(i)


  }
}



trueICC<-sd.pers^2/(sd.pers^2+sd.res^2)
ICCmean[(17+1):(2*17)]<-apply(intraperscorr,1,mean)
ICCbias[(17+1):(2*17)]<-ICCmean[(17+1):(2*17)]-trueICC
ICCsd[(17+1):(2*17)]<-apply(intraperscorr,1,sd)
trueCV<-sd.res/meanhyperalg
CVmean[(17+1):(2*17)]<-apply(CV,1,mean)
CVbias[(17+1):(2*17)]<-CVmean[(17+1):(2*17)]-trueCV
CVsd[(17+1):(2*17)]<-apply(CV,1,sd)
sqrtMSEmean[(17+1):(2*17)]<-apply(sqrt(MSEeblups),1,mean)

simresults<-data.frame(numberpers,numberobservers,numbertimepoints,
                       freqofmissing,ICCmean,ICCbias,ICCsd,CVmean,CVbias,
                       CVsd,sqrtMSEmean)
write.table(simresults, file.choose(),quote=F)

library(lattice)

group<-with(simresults,factor(freqofmissing))
levels(group)<-c("Scenario 1","Scenario 2")

simresults$group<-group


#Plots of ICC

plot1<-xyplot(ICCmean~numberpers,groups=group,key=simpleKey(levels(simresults$group)),type="l",
              data=simresults,ylab="Sample mean: ICC",xlab="Number of persons")
```

```
plot2<-xyplot(ICCbias~numberpers,groups=group,type="l",
              data=simresults,ylab="sample bias: ICC",xlab="Number of persons")

plot3<-xyplot(ICCsd~numberpers,groups=group,type="l",
              data=simresults,ylab="Sample standard error: ICC",xlab="Number of persons")

dev.off()
print(plot1,split=c(1,1,1,3),more=TRUE)
print(plot2,split=c(1,2,1,3),more=TRUE)
print(plot3,split=c(1,3,1,3))

#Plots of CV

plot1<-xyplot(CVmean~numberpers,groups=group,key=simpleKey(levels(simresults$group)),type="l",
              data=simresults,ylab="Sample mean: CV",xlab="Number of persons")

plot2<-xyplot(CVbias~numberpers,groups=group,type="l",
              data=simresults,ylab="sample bias: CV",xlab="Number of persons")

plot3<-xyplot(CVsd~numberpers,groups=group,type="l",
              data=simresults,ylab="Sample standard error: CV",xlab="Number of persons")
dev.off()
print(plot1,split=c(1,1,1,3),more=TRUE)
print(plot2,split=c(1,2,1,3),more=TRUE)
print(plot3,split=c(1,3,1,3))


#Plots of RMSE-eblups

xyplot(sqrtMSEmean~numberpers,groups=group,key=simpleKey(levels(simresults$group)),type="l",
       data=simresults,ylab="Sample mean: RMSE of eblups",xlab="Number of persons")

simresults50<-subset(simresults,numberpers==50)



simresults50


############################################################
numberpers numberobservers numbertimepoints freqofmissing
7          50              1                4           0.0
24         50              1                4           0.1
ICCmean      ICCbias      ICCsd    CVmean      CVbias
7  0.8249961 -0.003770991 0.03397677 0.2569119 0.006911918
24 0.8240489 -0.004718188 0.04022346 0.2574831 0.007483095
CVsd sqrtMSEmean      group
7  0.04919145    28.21741 Scenario 1
24 0.04916959    30.58030 Scenario 2
#############################################################
```
