# Supplementary material for: The Area of Secondary Hyperalgesia following Heat Stimulation in Healthy Male Volunteers: Inter- and Intra-Individual Variance and Reproducibility
Source: PLoS One. 2016 May 11;11(5):e0155284. doi: 10.1371/journal.pone.0155284 (PMC4864410; doi:10.1371/journal.pone.0155284)
Supplement: S4 Appendix — (DOCX) [file pone.0155284.s004.docx]

**PROTOKOL**

# TITEL

**Kan frivillige forsøgspersoners smertedisposition karakteriseres (fænotypes) på basis af deres udvikling af sekundær hyperalgesi målt ved kort termal sensibilisering?**

**Sponsor:**

Navn: Jørgen Berg Dahl

Titel: Professor

**Ansvarlig investigator:**

Navn: Morten Sejer Hansen

Tiltel: Læge, PhD -studerende

**Sub-investigatorer:**

Navn: Mohammed Sohail Asghar

Titel: Læge, PhD

Navn: Karen Lisa Hilsted

Titel: Forskningssygeplejerske

Navn: Emilia Horjales-Araujo

Titel: Msc. Biol., Lic. med., PhD

Navn: Rebecca Østervig

Titel: Scholarstipendiat, stud. Med.

Diverse navne, adresser, telefonnumre samt e-mailadresser af involverede i studiet.

- **Sponsor:**

Jørgen Berg Dahl

Professor, overlæge, dr.med., MMD

Anæstesi- og Operationsklinikken, HOC, Rigshospitalet

Blegdamsvej 9

2100 København Ø

Tlf.: 3545 3475

E-mail: [jbdahl@dadlnet.dk](mailto:jbdahl@dadlnet.dk)

- **Forsøgsansvarlig investigator:**

Morten Sejer Hansen

Læge, Cand. Med

Anæstesi og operationsklinikken, HOC, Rigshospitalet

Blegdamsvej 9

2100 København Ø

Tlf. 35453475

E-mail: [morten.sejer.hansen@regionh.dk](mailto:morten.sejer.hansen@regionh.dk)

- **Sub-investigator:**

Karen Lisa Hilsted

Forskningssygeplejerske

Anæstesi- og Operationsklinikken, HOC, Rigshospitalet

Blegdamsvej 9

2100 København Ø

Tlf.: 3545 9502

E-mail: [karen.lisa.hilsted@rh.regionh.dk](mailto:karen.lisa.hilsted@rh.regionh.dk)

Emilia Horjales-Araujo
Msc. Biol., Lic. med., PhD

Anæstesi- og operationsklinikken, HOC, Rigshospitalet

Blegdamsvej 9

2100 København Ø

Tlf.: 35453475

E-mail: [maria.emilia.horjales.araujo@regionh.dk](mailto:maria.emilia.horjales.araujo@regionh.dk)

Mohammed Sohail Asghar

Læge, PhD, Master of Disaster Management.

Anæstesi- og operationsklinikken, HOC, Rigshospitalet

Blegdamsvej 9

2100 København Ø

Tlf.: 20613737

E-mail: [sohail@dadlnet.dk](mailto:sohail@dadlnet.dk)

Rebecca Østervig

Scholarstipendiat, stud. Med.

Anæstesi- og operationsklinikken, HOC, Rigshospitalet

Blegdamsvej 9

2100 København Ø

Tlf.: 29920525

E-mail: rebeccaoestervig@hotmail.com

- **Samarbejdspartner:**

Jørn Wetterslev

Overlæge, PhD

Copenhagen Trial Unit, Centre for Clinical Intervention Research, Afd. 7812

Blegdamsvej 9

2100 København Ø

Tlf.: 45 3545 7159

E-mail: [Wetterslev@ctu.dk](mailto:Wetterslev@ctu.dk)

Christian Bressen Pipper

Lektor, Msc, PhD

Sektion for biostatistik, institut for folkesundhedsvidenskab, Det sundhedsvidenskablige fakultet, Københavns universitet

Øster Farigmagsgade 5

1014 København K

Tlf. nr.: 35327911

E-mail: [pipper@sund.ku.dk](mailto:pipper@sund.ku.dk)

**Indholdsfortegnelse**

[TITEL 1](#_Toc390945281)

[Diverse navne, adresser, telefonnumre samt e-mailadresser af involverede i studiet. 2](#_Toc390945282)

[Introduktion og rationale 5](#_Toc390945283)

[Baggrund 5](#_Toc390945284)

[Formål 8](#_Toc390945285)

[Outcomes 8](#_Toc390945286)

[Etiske overvejelser 8](#_Toc390945287)

[Overvejelser i forbindelse med afprøvningen som helhed: 8](#_Toc390945288)

[Risici, bivirkninger og ulemper mv. 9](#_Toc390945289)

[Information af og samtykke fra patienter 9](#_Toc390945290)

[Beskyttelse af data fra patienter 9](#_Toc390945291)

[Rammer for studiet 10](#_Toc390945292)

[Tidsplan 10](#_Toc390945293)

[Sted for undersøgelsens udførelse 10](#_Toc390945294)

[Studiedesign 10](#_Toc390945295)

[Studieselektion 10](#_Toc390945296)

[Inklusionskriterier 10](#_Toc390945297)

[Eksklusionskriterier 11](#_Toc390945298)

[Praktiske forhold 11](#_Toc390945299)

[Økonomiske forhold 11](#_Toc390945300)

[Forsøgspersoners gennemførelse og afbrydelse af forsøget 11](#_Toc390945301)

[Årsager til forsøgspersoners afbrydelse af forsøget 11](#_Toc390945302)

[Procedure for patienter, der afbryder forsøget 12](#_Toc390945303)

[Metodologi 12](#_Toc390945304)

[Generel fremgangsmåde 12](#_Toc390945305)

[Kliniske vurderinger 15](#_Toc390945306)

[Bivirkninger 16](#_Toc390945307)

[Bivirkninger/uønskede hændelser (AEs=adverse events) 16](#_Toc390945308)

[Retningslinier for uønskede hændelsers eventuelle sammenhæng med behandling 16](#_Toc390945309)

[Gradering af uønskede hændelser 17](#_Toc390945310)

[Rapportering af AEs og SAEs bivirkninger 17](#_Toc390945311)

[Statistiske analyser 17](#_Toc390945312)

[Beregning af antal patienter 17](#_Toc390945313)

[Databearbejdning 17](#_Toc390945314)

[Dataregistrering, samt regler for kontrol af undersøgelsesprocedurer 18](#_Toc390945315)

[Case Report Form 18](#_Toc390945316)

[Uddannelse 18](#_Toc390945317)

[Yderligere krav og generel information 19](#_Toc390945318)

[Forsikring 19](#_Toc390945319)

[Offentliggørelse af resultater 19](#_Toc390945320)

Introduktion og rationale

Baggrund

**Fysiologiske og disponerende faktorer for udvikling akutte og kroniske smerter**

Det antages at en række psykologiske og sociale faktorer har betydning for udvikling og oplevelse af smerte hos det enkelte individ.

Vi ved mindre om de fysiologiske mekanismer der betinger akut og kronisk smerte. Det vides såle-des ikke om, der er individer med et særligt overfølsomt smertesystem, og om en sådan overføl-somhed er genetisk betinget og dermed arvelig.([1](#_ENREF_1))

I givet fald vil en nedarvet tilbøjelighed kunne øge en risiko for udvikling af bl.a. svære akutte og kroniske smertetilstande. Smerteoverfølsomhed af centralnervesystemet antages at være en vigtig faktor ved en lang række forskellige smertetilstande, herunder fibromyalgi, slidgigt samt smerter efter operation.

Vor forskergruppe har stor erfaring med fysiologiske modeller til udløsning af kortvarig smerte-overfølsomhed hos forsøgspersoner.([2-4](#_ENREF_2)) Vi har i en analyse af data fra vores tidligere undersøgelser vist, at individer der udsættes for identiske smertestimuli udviser meget forskellige grader af smerteoverfølsomhed. Samtidig antyder disse undersøgelser at smerteoverfølsomhed er et fysiologisk og reproducérbart fænomen, der karakteriserer det enkelte individ.([4](#_ENREF_4))

Ved hjælp af billeddannende teknikker ((f)MRI) har vi endvidere vist, at der eksisterer såvel anatomiske som funktionelle forskelle i hjernen hos individer, der udvikler henholdsvis lav og høj smerteoverfølsomhed efter at have været udsat for identiske smertestimuli.([5](#_ENREF_5))

Endelig antyder vores undersøgelser en sammenhæng imellem udvikling af smerteoverfølsomhed, og psykisk vulnerabilitet. Disse fund er nye, og har ikke tidligere været publicerede.([5](#_ENREF_5))

Resultater fra andre forskergrupper antyder, at smerteoverfølsomhed er arvelig, med et anslået 50 % genetisk bidrag til smertevariationen. Endvidere, at smertefølsomhed vurderet inden en operation delvist afgør graden af smerter efter operation, hvilket bekræfter, at den er bestemt af individuelle faktorer.([6-8](#_ENREF_6))

**Hyperalgesi modeller og fænotyping af forsøgspersoner**

En standardiseret, førstegrads brandskade udløser hos for­søgs­personer reversibel primær og sekundær hy­peralgesi i et velaf­grænset hudområde.([9](#_ENREF_9), [10](#_ENREF_10)) Den primære hyperal­gesi opstår svaren­de til selve det traumatiserede område og er karakteriseret ved reducerede tærskler for såvel termale som mekaniske stimu­li. Den sekundære hy­peralgesi kan påvises omkrin­g det primære hyperalgetiske område, men adskiller sig fra dette ved udelukkende at frembyde reducerede tærskler for mekaniske stimu­li. Tidligere under­søgelser har vist, at den sekundære hyperalgesi skyldes et ændret centralt respons på signaler i ikke-noci­cepti­ve Aß-fibre, hvorfor denne forsøgsopstilling kan anvendes til at undersøge basale smertefysiologiske problemstillinger, samt forskellige medikamen­ters indflydel­se på traumeinducerede forandringer af såvel det perifere som det centrale nervesy­stem.([10](#_ENREF_10), [11](#_ENREF_11)) Sekundær hyperalgesi kan udløses med en række forskellige konditionerende stimuli og antages at være et robust reproducerbart fænomen.([10-12](#_ENREF_10)) Tidligere studier har dog undersøgt sekundær hyperalgesi med multiple stimulationsmodaliteter på samme forsøgsdag, hvorfor mulig carry-over effekt ikke kan udelukkes, og reproducerbarheden af den sekundære hyperalgesi derfor kræver yderligere undersøgelse.

Forskningsgruppens undersøgelse har påvist at forsøgspersoner, der udsættes for identiske smertestimuli efterfølgende, udviser meget forskellige grader af smerteoverfølsomhed. Samtidig har vi observeret, at graden af den individuelle overfølsomhed synes at være reproducérbar, hvorfor smerteoverfølsomhed antages at være et fysiologisk fænomen, der karakteriserer det enkelte individ.([4](#_ENREF_4))

**Association imellem smerteoverfølsomhed og psykologisk vulnerabilitet**

Forskningsgruppens undersøgelser har antydet, at der findes en association imellem graden af smerteoverfølsomhed og psykologisk vulnerabilitet, hos den enkelte forsøgsperson.([5](#_ENREF_5)) Endvidere har flere studier vist en association med psykologiske variabler og oplevelse af smerte.([13-15](#_ENREF_13))

Til vurdering af psykologisk vulnerabilitet kan der bl.a. anvendes Hospital Anxiety and Depression Scale (HADS),([16](#_ENREF_16)) og Pain Catasthrophizing Scale (PCS).([17](#_ENREF_17))

HADS består af et spørgeskema med i alt 14 spørgsmål, der sigter mod at identificere personer med symptomer på depression og angst. Systematiske reviews viser at HADS udmærker sig ved at være i stand til at screene for angst og depression, og er anvendelig både hos patienter og i den ikke-hospitaliserede population,([18](#_ENREF_18)) hvilket gør den egnet til brug hos raske forsøgspersoner

Pain Catastrophizing Scale (PCS)([17](#_ENREF_17)) udgøres af et 13 punkts spørgeskema der omhandler de tre centrale elementer i ”catastrophizing”; ruminering, forstærkning og hjælpeløshed. PCS er applicerbar hos indlagte patienter såvel som ikke-indlagte raske personer.([19](#_ENREF_19))

Vi ønsker med den aktuelle prospektive undersøgelse at undersøge om smerteoverfølsomhed er et fysiologisk og reproducérbart fænomen, der karakteriserer det enkelte individ. Endvidere ønsker vi at undersøge om der er association imellem central smerteoverfølsomhed (vurderet ved graden af sekundær hyperalgesi) og individuelle, psykologiske karakteristika, belyst ved HADS og PCS, hos frivillige mandlige forsøgspersoner. De fremtidige perspektiver vil derfor være at opnå en øget viden om det enkelte individs smertedisposition, samt om nervesystemets bearbejdning af smertestimuli, og muligvis kunne anvendes til individualiseret forebyggelse og behandling af akutte og kroniske smertetilstande

References

1. Woolf CJ. Central sensitization: implications for the diagnosis and treatment of pain. Pain. 2011;152(3 Suppl):S2-15. doi: 0.1016/j.pain.2010.09.030. Epub Oct 18.

2. Dirks J, Petersen KL, Dahl JB. The heat/capsaicin sensitization model: a methodologic study. J Pain. 2003;4(3):122-8.

3. Mathiesen O, Imbimbo BP, Hilsted KL, Fabbri L, Dahl JB. CHF3381, a N-methyl-D-aspartate receptor antagonist and monoamine oxidase-A inhibitor, attenuates secondary hyperalgesia in a human pain model. J Pain. 2006;7(8):565-74.

4. Werner MU, Petersen KL, Rowbotham MC, Dahl JB. Healthy volunteers can be phenotyped using cutaneous sensitization pain models. PLoS One. 2013;8(5):e62733. doi: 10.1371/journal.pone.0062733. Print 2013.

5. Pereira MP, Werner MU, Ringsted TK, Rowbotham MC, Taylor BK, Dahl JB. Does naloxone reinstate secondary hyperalgesia in humans after resolution of a burn injury? A placebo-controlled, double-blind, randomized, cross-over study. PLoS One. 2013;8(5):e64608. doi: 10.1371/journal.pone.0064608. Print 2013.

6. Norbury TA, MacGregor AJ, Urwin J, Spector TD, McMahon SB. Heritability of responses to painful stimuli in women: a classical twin study. Brain. 2007;130(Pt 11):3041-9. Epub 2007 Oct 11.

7. Tegeder I, Adolph J, Schmidt H, Woolf CJ, Geisslinger G, Lotsch J. Reduced hyperalgesia in homozygous carriers of a GTP cyclohydrolase 1 haplotype. Eur J Pain. 2008;12(8):1069-77. doi: 10.16/j.ejpain.2008.02.004. Epub Apr 18.

8. Williams FM, Scollen S, Cao D, Memari Y, Hyde CL, Zhang B, et al. Genes contributing to pain sensitivity in the normal population: an exome sequencing study. PLoS Genet. 2012;8(12):e1003095. doi: 10.1371/journal.pgen.. Epub 2012 Dec 20.

9. Latremoliere A, Woolf CJ. Central sensitization: a generator of pain hypersensitivity by central neural plasticity. J Pain. 2009;10(9):895-926. doi: 10.1016/j.jpain.2009.06.012.

10. Naert AL, Kehlet H, Kupers R. Characterization of a novel model of tonic heat pain stimulation in healthy volunteers. Pain. 2008;138(1):163-71. doi: 10.1016/j.pain.2007.11.018. Epub 8 Jan 22.

11. Petersen KL, Rowbotham MC. A new human experimental pain model: the heat/capsaicin sensitization model. Neuroreport. 1999;10(7):1511-6.

12. Staahl C, Olesen AE, Andresen T, Arendt-Nielsen L, Drewes AM. Assessing efficacy of non-opioid analgesics in experimental pain models in healthy volunteers: an updated review. Br J Clin Pharmacol. 2009;68(3):322-41. doi: 10.1111/j.365-2125.009.03433.x.

13. Lee JE, Watson D, Frey-Law LA. Psychological factors predict local and referred experimental muscle pain: a cluster analysis in healthy adults. Eur J Pain. 2013;17(6):903-15. doi: 10.1002/j.532-2149.012.00249.x. Epub 2012 Nov 19.

14. Sullivan MJ, Thorn B, Haythornthwaite JA, Keefe F, Martin M, Bradley LA, et al. Theoretical perspectives on the relation between catastrophizing and pain. Clin J Pain. 2001;17(1):52-64.

15. Papaioannou M, Skapinakis P, Damigos D, Mavreas V, Broumas G, Palgimesi A. The role of catastrophizing in the prediction of postoperative pain. Pain Med. 2009;10(8):1452-9. doi: 10.111/j.526-4637.2009.00730.x. Epub 2009 Oct 26.

16. Zigmond AS, Snaith RP. The hospital anxiety and depression scale. Acta Psychiatr Scand. 1983;67(6):361-70.

17. Sullivan MJL, Bishop SR, Pivik J. The Pain Catastrophizing Scale: Development and validation. Psychol Assess. 1995;7(4):524-32.

18. Bjelland I, Dahl AA, Haug TT, Neckelmann D. The validity of the Hospital Anxiety and Depression Scale. An updated literature review. J Psychosom Res. 2002;52(2):69-77.

19. Van Damme S, Crombez G, Bijttebier P, Goubert L, Van Houdenhove B. A confirmatory factor analysis of the Pain Catastrophizing Scale: invariant factor structure across clinical and non-clinical populations. Pain. 2002;96(3):319-24.

20. Smarr KL, Keefer AL. Measures of depression and depressive symptoms: Beck Depression Inventory-II (BDI-II), Center for Epidemiologic Studies Depression Scale (CES-D), Geriatric Depression Scale (GDS), Hospital Anxiety and Depression Scale (HADS), and Patient Health Questionnaire-9 (PHQ-9). Arthritis Care Res (Hoboken). 2011;63(Suppl 11):S454-66. doi: 10.1002/acr.20556.

Formål

Formålet med dette prospektive, observationelle og deskriptive studie er at validere tidligere resultater fra retrospektive undersøgelser der indikerer, at sekundær hyperalgesi er et robust og reproducerbart fænomen med lav intra-individuel variabilitet og stor inter-individuel variabilitet; samt at validere at graden af sekundær hyperalgesi kan anvendes som et fænotypisk fænomen, og biomarkør for centralnervesystemets excitabilitet. Endvidere at graden af sekundær hyperalgesi kan korreleres med individets psykiske vulnerabilitet.

**I en prospektiv kohorte på 50 frivillige forsøgspersoner vil vi:**

1. Bestemme graden af hyperalgesi udløst af konditionering med kort termal sensiblisering på fire forskellige forsøgsdage
2. Bestemme tærskler for varme-smerte (heat-pain-detection-threshold, HPDT) og smerter under opvarmning af huden til 45 °C i 1 min
3. Fastlægge forsøgspersonernes psykologiske vulnerabilitet, baseret på ”Hospital Anxiety and Depression Scale”, og ”Pain Catastrophizing Scale”

Outcomes

**På basis af ovenstående fænotypiske bestemmelser af forsøgspersonerne vil vi:**

**Primært outcome:**

Undersøge den intra-individuelle og inter-individuelle variabilitet, baseret på dag-til-dag målinger ved kort termal sensibilisering

**Sekundært outcome:**

Sammenligne psykisk vulnerabilitet vurderet ved PCS og HADS med størrelsen på arealet af det hyperalgetiske område.

Sammenligne tærskler for varme-smerte og smerter under opvarmning af huden til 45 °C i 1 minut med størrelsen på arealet af det hyperalgetiske område udløst ved kort termal sensibilisering.

Resultaterne fra ovenstående undersøgelser skal danne grundlag for beregning af materialestørrelser i fremtidige forsøg, specielt med henblik på videre undersøgelser af forskelle i psykologisk vulnerabilitet, strukturelle forskelle i det centrale nervesystem, samt genetiske polymorfier, hos høj- og lav-responders

Etiske overvejelser

Overvejelser i forbindelse med afprøvningen som helhed:

Ved anvendelse af kliniske smertemodeller på raske forsøgspersoner bliver det muligt at studere basale smertemekanismer hos raske mennesker under standardiserede forhold. Resultater fra sådanne undersøgelser kan anvendes til at optimere og/eller ændre væsentlige dele af den nuværende kliniske smertebehandling, til fordel for en stor patientpopulation.

Vores overordnede mål med dette studie er at vi i fremtiden ved simple undersøgelser kan fænotypere patienters smertedisposition, dvs. bestemme patienters risiko for udvikling af smerte, og derved bidrage til en øget individualisering af smertebehandlingen til den enkelte patient.

En optimeret akut smertebehandling vil muligvis kunne mindske forekomsten af kroniske smerter. Dette vil være af stor betydning for den enkelte patient, hvis livskvalitet højnes, samt have samfundsøkonomisk betydning, idet en stor del af de kroniske smertepatienter ofte helt eller delvist er udelukket fra arbejdsmarkedet.

Forsøget vil blive udført i overensstemmelse med principperne i Helsinki Deklarationen. Protokollen vil blive indsendt til den lokale Videnskabsetiske Komité og Datatilsynet til godkendelse. Investigator vil desuden informere den Videnskabsetiske komité og Datatilsynet om væsentlige eller større ændringer i protokollen. Undersøgelsen vil blive anmeldt på den internationale database [www.clinicaltrial.gov](http://www.clinicaltrial.gov)

Risici, bivirkninger og ulemper mv.

Kort termal sensibilisering med varmetermode medfører ingen skade på huden. I enkelte tilfælde kan ses forandringer der kan sammenlignes med de forandringer der ses i solskoldet hud, dvs. en meget let 1. Grads forbrænding.

En eventuel første grads forbrænding kan forventes at medføre rødme og let smerte/ubehag i op til 24 timer efter stimulus. I sjældne tilfælde kan der udvikles en mindre vabel, der forsvinder uden mén og behandling.

Det er vores vurdering, at bivirkninger og risici ved deltagelse i denne undersøgelse er minimale, hvorfor det skønnes at være forbundet med meget lav risiko at deltage i forsøget.

At deltage i forsøget forventes ikke at indebære yderligere gener eller undersøgelser end ovenfor beskrevet.

##

Information af og samtykke fra patienter

De raske frivillige mandlige forsøgspersoner vil blive rekrutteret ved hjælp af et opslag i det Sundhedsvidenskabelige fakultets blad for studerende, ”MOK”, i København.

De raske frivillige mandlige forsøgspersoner der henvender sig, vil få tilsendt en kopi af den skriftlige deltager information, og vil blive mundtligt informeret om at tage en bisidder med til den mundtlige information, hvis de ønsker dette. Forsøgspersonerne vil blive mundtligt informeret af den forsøgsansvarlige læge, i en form der er forståelig for dem, i et aflukket lokale hvorved informationen vil foregå uden unødig forstyrrelser. Der vil blive givet deltagerne den tid, de har behov for og mulighed for at få svar på spørgsmål.

Beskyttelse af data fra patienter

Forsøget vil blive anmeldt til Datatilsynet. Lov om behandling af personoplysninger vil blive overholdt.

Alle oplysninger vil blive behandlet fortroligt og ved indberetning af forsøgsresultater vil forsøgsdeltageren være anonym og de ansvarlige personer for dette forsøg er underlagt tavshedspligt.

Indsamlede data i form af Case Report Form, underskrevne informerede samtykkeformularer og journal oprettet til studiet vil kun blive gjort tilgængelige for inspektion af autoriserede repræsentanter fra relevante myndigheder.

Rammer for studiet

Tidsplan

Undersøgelsen forventes påbegyndt: d. 1/5-2014

Undersøgelsen forventes afsluttet: 1/6-2015

Sted for undersøgelsens udførelse

Rigshospitalet

Anæstesiologisk afd. 4231, HOC

Smertelaboratoriet

Studiedesign

Forsøgstype: Eksplorativt studie

Deltagere: Frivillige forsøgspersoner, mænd mellem 18-35 år.

Antal: 50 evaluerbare forsøgspersoner

Randomisering Rækkefølge af LTS og HPDT randomiseres efter randomiseringsskema udarbejdet af Copenhagen Trial Unit.

Blinding Besvarede psykologiske tests opbevares i uigennemsigtige konvolutter. Blindingen brydes når alle forsøgspersoner har gennemført alle 4 forsøgsdage, hvorefter data kan analyseres.

Studieselektion

Inklusionskriterier

Forsøgspersonerne skal opfylde alle følgende kriterier for at være egnede til inklusion i undersøgelsen:

- Alder ≥ 18 år og ≤ 35 år
- Skal kunne tale og forstå dansk
- Køn: Mand
- Forsøgspersoner, som har givet deres skriftlige informerede samtykke til at deltage i undersøgelsen efter at have forstået denne.
- Ingen receptpligtig medicin indenfor den sidste måned.

Eksklusionskriterier

Forsøgspersoner, der opfylder et eller flere af følgende kriterier, er ikke egnede til inklusion i denne undersøgelse:

- Forsøgspersoner, som ikke kan samarbejde til undersøgelsen.
- Forsøgspersoner med alkohol- og/eller medicinmisbrug – efter investigators skøn.
- Forsøgspersoner der har indtaget et eller flere analgetika 2 dage før undersøgelsen.
- Forsøgspersoner der indenfor den sidste måned har indtaget antidepressiva.
- Forsøgspersoner med kroniske smerter
- Forsøgspersoner med psykiatriske diagnoser
- Forsøgspersoner med tatoveringer på ekstremiteterne
- BMI>30 eller BMI<18

##

Praktiske forhold

Økonomiske forhold

Denne undersøgelse er investigator-initieret og iværksat af Professor Jørgen B. Dahl og læge og PhD-studerende Morten Sejer Hansen. Udgifter i forbindelse med studiet relaterer sig til aflønning af raske frivillige forsøgspersoner samt udgifter til udstyr. Forsøgspersonerne vil modtage 150,- kr. per time for deltagelse i forsøget (forventet 1 time til screening samt 4 besøg á 1 time til selve forsøget) – beløbet er skattepligtigt. Udgifter til forsøget vil blive søgt dækket fra diverse fonde. Disse penge vil blive sat ind på Professor Jørgen B. Dahls forskningsfond. Denne er underlagt offentlig revision. Forsøgspersonerne vil blive informeret hvis der opnås fondsstøtte. De forsøgsansvarlige er ansat på Anæstesi- og operationsklinikken, HOC, 4231, Rigshospitalet og har ingen økonomiske interesser i forsøget.

Forsøgspersoners gennemførelse og afbrydelse af forsøget

- En forsøgsperson, der har gennemført undersøgelsen, er en forsøgsperson, der har fulgt forsøgsplanen i de foreskrevne 4 forsøgsdage.
- En forsøgsperson, der ikke har gennemført forsøget, er en forsøgsperson, som inkluderes i forsøget, dvs. giver informeret samtykke og ikke gennemfører forsøget.
- Har en forsøgsperson ikke gennemført forsøget skal der redegøres for om, eller hvorledes denne følges i øvrigt i studiet, - dette gælder også drop-outs - samt hvilke data der indsamles fra disse forsøgspersoner.

Årsager til forsøgspersoners afbrydelse af forsøget

En forsøgsperson kan tages ud af forsøget under følgende omstændigheder:

- Hvis investigator skønner, at en ændring af forsøgsplanen vil være det bedste for forsøgspersonen
- Hvis forsøgspersonen ønsker at udgå af forsøget
- Overtrædelse af protokollens regler

##

Procedure for patienter, der afbryder forsøget

I overensstemmelse med Helsinki Deklarationen har forsøgspersonerne ret til at afbryde forsøget på ethvert tidspunkt af hvilken som helst årsag. Investigator har også ret til at trække en patient ud af forsøget på ethvert tidspunkt.

Årsagen til at en patient tages ud af forsøget før planlagt, skal noteres i patientens Case Report Form.

Metodologi

Generel fremgangsmåde

På forsøgsdagene testes alle forsøgspersoner liggende fladt på ryggen. Undersøgelsen finder sted i et stille aflukket rum, hvor kun forsøgspersonen og investigator er til stede.

Forsøgspersonerne testes med 1 smerte-konditioneringstype, kort termal sensibilisering, på 4 forskellige forsøgsdage hvor der bestemmes sekundær hyperalgesi (se afsnit: Kliniske vurderinger). Yderligere foretages der 2 undersøgelser: Varme-smerte tærskel (Heat-pain detection threshold, HPDT) og long thermal stimulation (LTS) (se afsnit: Kliniske vurderinger). HPDT og LTS foretages efter kort termal sensibilisering, men rækkefølgen af stimulationerne (HPDT og LTS) randomiseres for hver patient og forsøgsdag efter randomiseringsskema udarbejdet af Copenhagen Trial Unit, så denne varierer tilfældigt for forsøgsperson og fra forsøgsdag til forsøgsdag. Observatørerne, investigatorer med ansvar for stimulation og dataregistrering, tildeles forsøgsdage således at samme observatør ikke forekommer på 2 konsekutive forsøgsdage. Der er i alt 2 forskellige observatører.

Forsøgspersonerne undersøges med en række forskellige testtyper:

1. Kort termal sensibilisering (Brief thermal sensitization = BTS). Målingen foretages midt på den anteriore del af det højre lår til tiden 0 min. (se afsnit: Kliniske vurderinger).
2. Varme-smerte tærskel (Heat pain detection threshold, HPDT). Målingen foretages midt på den anteriore del af den dominante underarm, til tiden 7 eller 10 min. afhængig af randomiseringen (se afsnit: Kliniske vurderinger).
3. Long thermal stimulation (LTS). Målingen foretages anteriort midt på den ikke-dominante underarm. Der foretages kontinuerlig VAS-måling og der beregnes en middel, og maksimum VAS. LTS foretages til tiden 7 eller 10 min. afhængig af randomiseringen (se afsnit: Kliniske vurderinger).

Forsøget strækker sig over 4 identiske forsøgsdage samt en screeningsdag før forsøget går i gang. Forsøgsdagene og screeningsdagen vil forløbe således:

Screening

- Mundtlig information om projektet gives af forsøgsansvarlig. Der vil blive givet den betænkningstid, deltagerne har brug for.
- Kort journaloptagelse ved forsøgsansvarlig investigator, hvor in- og eksklusionskriterier gennemgås, højde, vægt, BT og puls måles.
- Konditioneringstests (BTS, HPDT og LTS) afprøves, for at gøre deltagerne kendt med proceduren.

Der udleveres spørgeskemaer ”Hospital anxiety and depression scale” og ”Pain catastrophizing scale” (på dansk) samt en uigennemsigtig konvolut hvori de besvarede spørgeskemaer skal lægges i ved aflevering på forsøgsdag 1. Eventuelle spørgsmål eller uklarheder omhandlende de to spørgeskemaer vil blive besvaret.

Forsøgsdag 1-4. Der skal minimum være 7 dage mellem hver forsøgsdag:

- Der kontrolleres, at der foreligger underskrevet samtykke og fuldmagt.
- Forsøgspersoner udleverer besvarede psykologiske tests, PCS og HADS, i en lukket uigennemsigtig konvolut (forsøgsdag 1). Konvolutten åbnes først når alle forsøgspersoner har gennemført alle forsøgsdage.
- Forsøgsperson placeres på et leje, fladt liggende på ryggen.
- Forsøget påbegyndes: Nedenfor ses en detaljeret gennemgang af forsøget punkt for punkt med tidsangivelser.
  1. Tiden 0 min: Måling af BTS
  2. Tiden 7 min: Måling af enten LTS eller HPDT (afhængig af randomisering)
  3. Tiden 10 min: Måling af enten LTS eller HPDT (afhængig af randomisering)

**Kliniske vurderinger**

**Vurdering af akut smerte**

Visuel analog skala,(VAS) index fra 0-100mm, hvor 0 mm repræsenterer ”ingen smerte” og 100mm repræsenterer den ”værste smerte”.

**Kort termal sensibilisering (Brief thermal sensitization)**

En computer-kontrolleret termode placeres på forsøgspersonens hud og varmes op til 45 °C i 3 min. Efter 3 min. testes for sekundær hyperanalgesi (se nedenfor), mens elektroden stadig er 45 °C og placeret på huden. BTS foretages til tiden 0 min.

**Vurdering af sekundær hyperanalgesi**

Arealet af hyperalgesi kvantificeres efter stimulering med et 19G von Frey hår. Grænsen for hyperalgesi bestemmes ved at stimulere langs fire linjer der danner et kvadrat omkring stimuleringsstedet, indtil deltageren angiver en klar ændring i fornemmelsen. Grænserne vil blive markeret med en filtpen og afstandene til senere overfladeberegning vil blive målt.

**Long thermal stimulation/lang termal stimulation**

Long thermal stimulation (LTS) består i at opvarme huden til 45 °C ved hjælp af den samme computer-kontrollerede termode i 1 min. Deltageren skal vurdere smerten på en elektronisk Visuel Analog Skala (VAS), som er en VAS- lineal tilsluttet computeren, som i hver ende er mærket med beskrivelsen “ingen smerte” (0) og “værst mulig smerte” (100). Smerten måles kontinuerligt i 1 min. idet smerte normalt fluktuerer under denne måling. Det elektroniske udstyr beregner automatisk en middel VAS og en maksimum VAS i denne tidsperiode. Deltageren vil ikke kunne se VAS målingen på skærmen under selve målingen og hver VAS vurdering er uafhængig af den tidligere.

Måling af LTS foretages til tiden 7 eller 10 min. afhængig af randomiseringen.

**Varme-smerte tærskel (Heat-pain-detection threshold, HPDT)**

Tærskelværdi, hvor varme udløser smerte (heat pain detection threshold, HPDT) repræsenterer den laveste temperatur, som opfattes som smertefuld. Starttemperaturen for termoden er 32°C, og hastigheden for temperaturøgningen er 1°C/sek. Forsøgspersonen bedes om at angive sin tærskel ved at trykke på en knap. Hvis grænsen for temperaturøgning på 52 °C nås før der registreres en tærskelværdi, går termoden automatisk tilbage til sin starttemperatur og 52 °C registreres. Hver tærskelværdi beregnes som gennemsnittet af fire stimuleringer; stimuleringerne lægges med 6-10 sek. mellemrum. Målingen af HPDT foretages til tiden 7 eller 10 min. afhængig af randomiseringen.

**Hospital Anxiety and Depression Scale (HADS)**

HADS består af et spørgeskema med i alt 14 spørgsmål, og er en 4-point Likert skala der går fra 0-3, og kan underopdeles i HADS-A der omhandler angst og HADS-D der omhandler depression. Den maksimale samlede score der kan opnås er 42, og en samlet vurdering af HADS vil være en samlet vurdering af forsøgspersonens generelle ”distress”.^15^ Hvis man ønsker at screene for angst og/eller depression, skal henholdsvis HADS-A og HADS-D vurderes separat. Den samlede score i de 2 subtests vil derfor være 21 for henholdsvis HADS-A og HADS-D.([20](#_ENREF_20)) Scoren opnået i henholdsvis HADS-A og HADS-D fortolkes således:

- 0-7: Normal
- 8-10: Mild
- 11-15: Moderat
- >16: Svær

Spørgeskemaet besvares inden første forsøgsdag.

**Pain Catastrophizing Scale (PCS)**

PCS^16^ består af et spørgeskema med i alt 13 spørgsmål. PCS er en 5 punkts Likert skala der går fra 0-4, og kan underopdeles i 3 subskalaer der hver især omhandler ruminering, forstærkning og hjælpeløshed.

Den maksimale score er 52, og ved vurdering af de 3 subskalaer findes den maksimale score ved at summere svarene efter nedenstående skema:

- Ruminering: Sum af spørgsmål 8, 9, 10, 11. Maksimal sum på 16 point.
- Forstærkning: Sum af spørgsmål 6, 7, 13. Maksimal sum på 12 point.
- Hjælpeløshed: Sum af spørgsmål 1, 2, 3, 4, 5, 12: Maksimal sum på 24

Spørgeskemaet besvares inden første forsøgsdag.

Bivirkninger

Bivirkninger/uønskede hændelser (AEs=adverse events)

Bivirkninger defineres som enhver skadelig og uønsket hændelse, tegn eller symptomer, der optræder under deltagelse i undersøgelsen, som er tidsrelateret til udførsel af forsøget hvad enten denne uønskede hændelse anses for at have forbindelse med undersøgelsen eller ej. Alle uønskede hændelser skal noteres i patientens Case Report Form. Hvis en utilsigtet hændelse opstår mere end 48 timer efter seneste udførsel af forsøget, og der ikke er en tilsyneladende årsagssammenhæng eller forbindelse med undersøgelse, anses denne ikke for en utilsigtet hændelse.

Start og slutdato/tidspunkt, sværhedsgrad og følger efter målingerne skal noteres for enhver uønsket hændelse. Sværhedsgraden af den uønskede hændelse og sammenhængen med målingerne skal vurderes i overensstemmelse med nedenfor beskrevne retningslinjer.

Investigator skal vurdere sammenhæng mellem en uønsket hændelse og målingerne ved hjælp af følgende retningslinjer:

Retningslinier for uønskede hændelsers eventuelle sammenhæng med behandling

1. Ikke relateret - ingen tidsmæssig sammenhæng, andre ætiologier meget sandsynligt årsagen
2. Muligvis relateret - mindre klar sammenhæng, andre ætiologier er også mulige
3. Sandsynligvis relateret - klar tidsmæssig sammenhæng med bedring ved afbrydelse af medicinering, og ikke rimeligt forklaret ved patientens kendte kliniske tilstand.
4. Relateret - klar tidsmæssig sammenhæng med genbehandlingstest eller klinisk vurdering.

Forsøgspersoner, der har uønskede hændelser, vil blive monitoreret med relevante kliniske vurderinger og laboratorieundersøgelser efter behandlende læges beslutning. Alle uønskede hændelser vil blive fulgt til tilfredsstillende restituering eller stabilisering.

Ved en alvorlig uønsket hændelse forstås en hændelse uanset forsøgsudførsel som medfører en betydelig risiko for død eller handikap hos patienten inkluderende, men ikke begrænsende sig til, en hændelse som resulterer i:

- død
- er livstruende – forsøgspersonen var efter investigators skøn i umiddelbar risiko for at dø af den uønskede hændelse, da den optrådte
- medfører hospitalsindlæggelse
- er varigt invaliderende

Gradering af uønskede hændelser

Den lægelige investigator skal forsøge at finde frem til alle kliniske og objektive reaktioner fra forsøgspersonen i behandling og fastslå deres sammenhæng med forsøget. Reaktioner, hvis der er nogen, skal graderes efter følgende skala:

1 = let

2 = moderat

3 = svær

4 = livstruende

##

Rapportering af AEs og SAEs bivirkninger

Investigator er ansvarlig for at alle uønskede hændelser skal registreres i patientens Case Report Form.

Investigator er ansvarlig for den løbende overvågning af forsøgets risk/benefit forhold. Opstår eller erkendes der situationer, der kan have betydning for forsøgspersonernes sikkerhed eller forsøgets udførelse skal dette **altid** straks rapporteres til alle involverede investigatorer og Videnskabsetiske Komiteer.

Sponsor skal af investigator løbende holdes orienteret om adverse events. Ved forsøgets afslutning skal den endelige rapport indeholde en beskrivelse af alle indtrufne bivirkninger.

Statistiske analyser

Beregning af antal patienter

Estimationen af det nødvendige antal på 50 forsøgspersoner og 4 forsøgsdage er *a priori* foretaget på baggrund af tidligere gennemførte forsøg.([2](#_ENREF_2)) Disse forsøg har dog ikke stringent adskilt BTS stimulation fra andre stimulationsmetoder og kan teoretisk set have rapporteret ’carry over’ effekter ligesom det primære formål i disse forsøg ikke har været estimationen af forholdet mellem intra- og inter-individuel variation. Selvom forholdet mellem den intra-individuelle variation og den inter-individuelle variation vil vise sig at være større end de tidligere rapporterede 25% ønsker vi at tage højde for dette ved at anvende et lidt større antal forsøgspersoner og forsøgsdage end i tidligere forsøg for stadig at kunne detektere en evt. reproducerbarhed af hyperalgesi arealer hos den enkelte forsøgsdeltager. Den til forsøget tilknyttede statistiker vil inden forsøgets start ved simulering af mulige forsøgsresultater på baggrund af de tidligere rapporterede forsøgsresultater præcisere estimationen af antal forsøgsdeltagere og forsøgsdage i forhold til valgte relevante scenarier for ønsket præcision af varians bestemmelserne. Simulationen vil desuden inkludere scenarier med op til 10% missing data i et missing at random (MAR) scenario hvor risikoen for missingness er forøget på forsøgsdage efter 1. forsøgsdag hos personer med store hyperalgesi arealer på 1. forsøgsdag.

Databearbejdning

Den primære analyse for det primære formål vil blive en varianskomponentanalyse m.h.p. at bestemme variansen på hyperalgesiarealer hidrørende fra henholdsvis forsøgsperson, forsøgsdag og observatør og estimere hvilke af disse 3 varianstyper der er mest betydende. For evt. association mellem hyperalgesiareal og depressions/angst score vil der blive foretaget lineær regression med estimering af 95% konfidens- og prædiktionsintervaller for middelværdi og placeringen af 95% af forsøgspersonerenes prædikterede værdi af depressions/angst score for et givet hyperalgesiareal. ’Limits of agreements’ plot for overensstemmelsen mellem hyperalgesiarealer på forskellige forsøgsdage og forskellige observatører vil blive udarbejdet og evalueret i overensstemmelse med relevante kriterier for klinisk reproducerbarhed. For alle analyser vil der blive tilstræbt foretaget en intention-to-treat analyse inkluderende alle forsøgspersoner der har deltaget i 1. forsøgsdag medmindre missingness mønstret er missing complete at random (MCAR) i hvilket tilfælde analyserne vil blive foretaget udelukkende på forsøgspersoner med komplette data. Evt. missing data vil blive imputeret ved hjælp af multiple imputation såfremt missingness er større end 5% og Littles test er signifikant så MCAR kan udelukkes og MAR ikke kan udelukkes. P<0.05 vil blive betragtet som statistisk signifikant og mulig indflydelse af anvendelsen af multiple test på statistisk signifikans vil blive vurderet efter family wise error justering, samt single step adjustment

Dataregistrering, samt regler for kontrol af undersøgelsesprocedurer

Undersøgelsen skal gennemføres i overensstemmelse med de gældende regler for kliniske forsøg, der omfatter mennesker vedrørende kvalitetskontrol og kvalitetsstyring. Investigator er tilknyttet Rigshospitalet og er ansvarlige for håndtering og arkivering af data efter gældende regler herunder lov om behandling af personoplysninger og sundhedsloven.

Data tilhører sponsor.

Projektet er anmeldt til datatilsynet.

Case Report Form

For hver patient inkluderet i undersøgelsen vil en Case Report Form (CRF) blive udfyldt. Sourcedata vil bestå af følgende komponenter

- Alder (år)
- Højde, vægt og BMI
- Blodtryk og puls
- Pain catastrophizing scale (PCS), udfyldt.
- Hospital pain and anxiety scale (HADS), udfyldt.
- For forsøgsdag 1-4:
- Resultat af Varme-smerte tærskel.
- Resultat af Long thermal stimulation.
- Resultat af Brief thermal sensitization
- Beregnet areal af Brief thermal sensitization
- Afkrydsningsfelter der indikerer følgende er udført: BTS udført, LTS udført, HPDT udført, BTS-areal beregnet, PCS modtaget, HADS modtaget.
- Felt til udfyldning af eventuelle bivirkninger

Uddannelse

Koordinerende investigator Morten Sejer Hansen og/eller forskningssygesygeplejerske, Karen Lisa Hilsted, vil sikre, at det involverede personale er passende uddannet og instrueret og har de fornødne oplysninger til udførelse af undersøgelsen.

Yderligere krav og generel information

##

Forsikring

For frivillige forsøgspersoner er gældende, at med hensyn til enhver skade, forårsaget direkte eller indirekte af undersøgelsesmetoderne i denne kliniske undersøgelse, påtager Rigshospitalet, Anæstesi- og operationsklinikken, HOC, sig det lovmæssige ansvar på investigator og dennes medarbejderes vegne. Forudsat at investigator og dennes medarbejdere har fulgt de instruktioner, som er givet i denne protokol samt eventuelle tillæg dertil. Samt at investigator og dennes medarbejdere har udført undersøgelsen videnskabeligt og i overensstemmelse med gældende regler og accepterede teknikker.

Forsøgspersoner er i tilfælde af skade eller død uden sammenhæng med undersøgelsens gennemførelse forsikret af hospitalets forsikring.

Offentliggørelse af resultater

På basis af data vil investigator skrive en rapport over undersøgelsen. Denne rapport vil blive fremsendt til relevante myndigheder. Rapporten vil også danne basis for et manuskript til publikation med følgende forfatterrækkefølge:

- - - 1. Morten Sejer Hansen
      2. Rebecca Østervig
      3. Jørn Wetterslev
      4. Christian Bressen Piper
      5. Mohammed Sohail Asghar
      6. Emilia Horjales-Araujo
      7. Karen Lisa Hilsted
      8. Jørgen Berg Dahl

Såvel negative som positive forsøgsresultater, samt inkonklusive resultater vil blive offentliggjort.
