# Supplementary material for: The Area of Secondary Hyperalgesia following Heat Stimulation in Healthy Male Volunteers: Inter- and Intra-Individual Variance and Reproducibility
Source: PLoS One. 2016 May 11;11(5):e0155284. doi: 10.1371/journal.pone.0155284 (PMC4864410; doi:10.1371/journal.pone.0155284)
Supplement: S5 Appendix — (DOCX) [file pone.0155284.s005.docx]

**PROTOCOL**

# **TITLE**

**Can the pain disposition of healthy volunteers be characterized based on the development of secondary hyperalgesia following brief thermal sensitization?**

**Sponsor:**

Name: Jørgen Berg Dahl

**Primary investigator:**

Name: Morten Sejer Hansen

Title**:** MD

**Sub investigators:**

Name: Mohammed Sohail Asghar

Title: PhD, M.D.

Name: Karen Lisa Hilsted

Title: Research nurse

Name: Emilia Horjales-Araujo

Title: Msc. Biol., Lic. med., PhD

Name: Rebecca Østervig

Title: Medical student

# **Name and addresses:**

- **Sponsor**:

Jørgen B. Dahl

Dr.med., MMD

Department of Anesthesiology

Bispebjerg and Frederiksberg Hospitals

Bispebjerg Bakke 23

2400 Copenahagen

Mobile: 2137 9772

E-mail: jbdahl@dadlnet.dk

- **Primary investigator:**

Morten Sejer Hansen

M.D.

Department of Anesthesiology, 4231

Centre of Head and Orthopedics

Rigshospitalet, Copenhagen, Denmark

Blegdamsvej 9

2100 Copenhagen, Denmark

E-mail: [morten.sejer.hansen@regionh.dk](mailto:morten.sejer.hansen@regionh.dk)

- **Sub investigators:**

Karen Lisa Hilsted

Research nurse

Department of Anesthesiology, 4231

Centre of Head and Orthopedics

Rigshospitalet, Copenhagen, Denmark

Blegdamsvej 9

2100 Copenhagen, Denmark

Telephone.: 3545 9502

E-mail: [karen.lisa.hilsted@rh.regionh.dk](mailto:karen.lisa.hilsted@rh.regionh.dk)

Emilia Horjales-Araujo
Msc. Biol., Lic. med., PhD

Department of Anesthesiology, 4231

Centre of Head and Orthopedics

Rigshospitalet, Copenhagen, Denmark

Blegdamsvej 9

2100 Copenhagen, Denmark

Telephone.: 35453475

E-mail: [maria.emilia.horjales.araujo@regionh.dk](mailto:maria.emilia.horjales.araujo@regionh.dk)

Mohammed Sohail Asghar

M.D., PhD, Master of Disaster Management.

Department of Anesthesiology, 4231

Centre of Head and Orthopedics

Rigshospitalet, Copenhagen, Denmark

Blegdamsvej 9

2100 Copenhagen, Denmark

Telephone.: 20613737

E-mail: [sohail@dadlnet.dk](mailto:sohail@dadlnet.dk)

Rebecca Østervig

Medical student

Department of Anesthesiology, 4231

Centre of Head and Orthopedics

Rigshospitalet, Copenhagen, Denmark

Blegdamsvej 9

2100 Copenhagen, Denmark

E-mail: rebeccaoestervig@hotmail.com

- **Collaborators:**

Jørn Wetterslev

Overlæge, PhD

Copenhagen Trial Unit, Centre for Clinical Intervention Research, dep. 7812

Blegdamsvej 9

2100 København Ø

Telephone.: 45 3545 7159

E-mail: [Wetterslev@ctu.dk](mailto:Wetterslev@ctu.dk)

Christian Bressen Pipper

Lektor, Msc, PhD

Section of biostatistics, Faculty of health, Copenhagen University

Øster Farigmagsgade 5

1014 Copenhagen, Denmark

E-mail: [pipper@sund.ku.dk](mailto:pipper@sund.ku.dk)

Telephone.: 35327911

E-mail: [pipper@sund.ku.dk](mailto:pipper@sund.ku.dk)

# **Introduction and background**

**Physiologic and predisposing factors in the development of acute and chronic pain**

The experience and development of pain may be influenced by a number of physiological, psychological and psychosocial factors. However, our knowledge of the mechanisms responsible for acute and chronic pain is still incomplete. We now little of the physiological mechanisms responsible for acute and chronic pain, thus it is yet unknown whether individuals with a particular sensitive pain perception exists, and if this ability is hereditary. Hereditary pain sensitivity may increase the risk of developing acute and chronic pain pathology.

It has been hypothesized that the predisposition for developing central sensitization in response to injury is hereditary, and may explain differences in susceptibility to developing severe acute, and chronic pain(1). Thus, central neuronal sensitization is presumed to play an important role in a number of different pain conditions, such as osteoarthritis, fibromyalgia and post-operative pain (1).

In order to investigate the basic physiological mechanisms of pain, our research group has worked extensively with experimental physiological pain models (2-4). In an analysis of previous studies we have demonstrated that healthy individuals exposed to identical pain stimuli demonstrate very different degrees of secondary hyperalgesia areas. Concomitantly, results from these studies suggest, that secondary hyperalgesia areas may be a physiological phenomenon that can be identified. Further, that areas of secondary hyperalgesia may predict pain responses of the single individual (4).

In a preliminary study we further investigated subjects with large and small areas of secondary hyperalgesia, by applying imaging techniques ((f)MRI), and demonstrated anatomical and functional cerebral differences between these phenotypes (5). Finally, our investigations suggest that pain sensitivity and psychological vulnerability may be associated(5).

Results from other studies indicate that pain sensitivity may be hereditary with a 50% genetic contribution to the pain variation. Moreover, that pain sensitivity evaluated preoperatively, may predict the magnitude of the postoperative pain. This confirms that pain sensitivity may be influenced by individual factors. (6-8)

**Hyperalgesia models and phenotyping of study participants**

A standardised burn injury in the skin provokes reversible primary and secondary hyperalgesia in healthy volunteers. Injury-induced primary hyperalgesia is located in the traumatised area and is characterised by reduced thresholds for thermal and mechanical stimulation. Secondary hyperalgesia is located around the traumatised area, and is characterised by reduced thresholds for mechanical stimulation only (9, 10).

Previous studies have demonstrated that secondary hyperalgesia is a result of central changes in part due to sensitization in response to nerve signals transmitted in non-nociceptive Aβ-nervefibers (1, 9). Thus, this method can be used in investigation of the basic physiologic pain response.

Secondary hyperalgesia can be provoked by a number of different conditioning stimuli, and according to previous studies this is a robust phenomenon (10-12). Hence, this method can be used to investigate basic pain physiology, and applied when investigating different pharmaceutical interventions in reducing secondary hyperalgesia.

Previous studies have investigated secondary hyperalgesia with multiple stimulation modalities, and possible carry-over effect cannot be ruled out. Studies investigating the reproducibility of the area of secondary hyperalgesia are therefore needed.

**Association between pain sensitivity and psychological vulnerability**

Finally, our studies indicate that an association between psychological vulnerability and secondary hyperalgesia areas may exist (5). Several clinical studies have demonstrated an association between psychological variables and the experience of pain (13-15). To evaluate psychological vulnerability, the two psychological tests, the Pain Catastrophizing Scale (PCS) (16) and the Hospital Anxiety and Depression Score (HADS) supplement each other (17). HADS is a 14 point questionnaire that aims to identify symptoms of depression and anxiety, and is applicable in both a hospitalised and non-hospitalised population (18). PCS is a 13 point questionnaire designed to identify the three central elements in catastrophizing: Rumination, magnification, and helplessness, and is also applicable in both the hospitalised and non-hospitalised population (19).

With this prospective study we aim to investigate if pain sensitivity, evaluated by areas of secondary hyperalgesia following a standardised heat injury, is a physiological and reproducible phenomenon that characterizes the single individual. Furthermore we aim to investigate the association between the areas of secondary hyperalgesia and the individual psychological characteristics evaluated by PCS and HADS in healthy male volunteers.

# **Aim**

The aim with this prospective, observational and descriptive study is to validate previous results from retrospective investigations that indicate that secondary hyperalgesia is a robust and reproducible phenomenon, with a low intra-individual and a high inter-individual variability. Moreover, we aim to validate that the degree of secondary hyperalgesia can be applied as a phenotypic phenomenon and a biomarker for the excitability of the central nervous system. Lastly we aim to investigate if the area of secondary hyperalgesia can be associated with the individual psychological vulnerability in the study participants.

In a prospective cohort of 50 healthy volunteers we aim to:

1. Investigate the size of the area of secondary hyperalgesia following brief thermal sensitization on four different study days.
2. Investigate heat pain detection thresholds and pain during 1 min. thermal stimulation of the skin.
3. Determine the psychological vulnerability evaluated by the Hospital Anxiety and Depression Scale and the Pain Catastrophizing Scale.

# **Outcomes**

**Primary outcome**

To determine the intra- and inter-individual variance, and the inter- and intra-observer variance of the secondary hyperalgesia areas following brief thermal sensitization on 4 separate experimental days with two different observers.

**Secondary outcomes**

To evaluate how precise the scores of PCS and HADS, total and sub-scores, predict the size of the area of secondary hyperalgesia following brief thermal sensitization.

To evaluate how precise HPDT and pain during 1 min. thermal stimulation of the skin (LTS) predict the size of the area of secondary hyperalgesia following brief thermal sensitization.

The results from this study are to be used in evaluating sample size in future studies.

# **Ethics**

## **Considerations in connection with the study as a whole**

By applying experimental pain models on healthy volunteers it is possible to study basic pain mechanisms under standardised conditions. Results from such studies may be used to optimise or change current clinical analgesic therapies, which may benefit a large population of patients.

Our overall aim with this study is that we in the future, by simple tests will be able to phenotype patients and estimate their risk of developing pain, thus estimating their pain disposition. An individualised approach to pain and analgesic treatment may be a way to reduce the incidence of acute and chronic pain, by individualising the analgesic regime.

A reduction of the incidence of acute and chronic pain will be of substantial significance to the individual patient. This will in addition also have a great socioeconomic importance, given that a large proportion of the patients with chronic pain are fully or partly excluded from active work.
The study will be conducted in accordance with the principles of the Declaration of Helsinki. The protocol will be submitted to the local Research Ethics Committees and the Danish Data Protection Agency for approval. The investigator will also inform the Research Ethics Committee and the Danish Data Protection Agency of any significant or major changes in the protocol. The study will be registered in the international database clinicaltrials.gov.

## **Risks, adverse effects, disadvantages, etc.**

Brief Thermal Sensitisation with computer-controlled thermode does not cause skin damage. In rare cases, changes comparable with a first degree sun burn may appear.

Long thermal Stimulation and evaluation of Heat Pain Detection Thresholds do not cause skin damage, and is not associated with any known adverse effects. It is our assessment that adverse effects and risks in this study are very low, hence, participation in this study is considered to be of very low risk.

Participation in the study is not expected to result in further harm, tests or examinations than described above.

## **Information about and consent from patients**

The study participants will be recruited by advertisement in the student magazine “Medicinske studenter Organisationers Kommunikationsorgan” (MOK), at the Faculty of Health, Copenhagen University, and on the website: [www.forsøgspersoner.dk](http://www.forsøgspersoner.dk).

The volunteers that sign up for the study will receive a copy of the written study information for participants, and the participant is verbally informed about the possibility of having a companion present to the following interview. At the interview the volunteer is verbally informed about the study in an easy comprehensible form. The verbal information will be given by one of the investigators. The interview will be conducted in a closed room without distractions or interruptions. The volunteer will be given time to consider the request.

All volunteers taking part in the study must provide their oral and written informed consent before they are included in the study. It is the investigator's duty to inform the participants so that they are fully aware of all aspects of the study.

Participants may at any time withdraw their consent to participate in the study. If the participant decides to do this, this will not impair the participant’s relationship with the investigator and the investigator will not question the decision of the participant.

## **Protection of patient data**

The study will be submitted to the Danish Data Protection Agency. Relevant legislation concerning personal information will be followed.

All information will be treated confidentially and in the reporting of test results, patients will be anonymous and the persons responsible for this trial are bound to confidentiality.

Data collected in the form of a Case Report Form, signed informed consents, and records will only be made available to authorised representatives from relevant authorities, in accordance with Danish law.

# **FRAMEWORK FOR THE STUDY**

## **Schedule**

The study is scheduled to start: 1/5-2014

The study is scheduled for completion: 1/6-2015

## **Location of the study:**

Rigshospitalet

Department of Anaesthesia 4231, HOC, Rigshospitalet.

Pain laboratory.

## **Study Design**

Type of trial: Explorative study

Participants: Healthy volunteers, males, age: 18-35 years.

Number: 50

Randomization: Sequential order of LTS and HPDT will be randomized in accordance with randomization schedule developed by Copenhagen trial unit.

Blinding: Completed psychological tests will be kept in opaque sealed envelopes. The blinding will be broken when all volunteers have completed the four study days.

# **STUDY SELECTION**

## **Inclusion criteria.**

Patients must meet all the following criteria to be suitable for inclusion in the study:

- Age >18 years and <35 years
- Speak and understand Danish
- Male gender
- Study participants who have understood and signed the informed consent
- No prescription medicine during the last 30 days.

## **Exclusion criteria.**

Study participants who meet one or more of the following criteria are not suitable for inclusion in this study:

- Study participants that cannot cooperate to the test.
- Study participants with an alcohol and/or substance abuse, assessed by the investigator
- Study participants with a substance abuse, assessed by the investigator.
- Study participants, who have consumed analgesics less than 2 days before experimental day.
- Study participants, who have consumed antidepressant medication during the last 30 days before experimental day.
- Study participants with chronic pain.
- Study participants with psychiatric diagnoses.
- Study participants with tattoos on the extremities.
- Study participants with a Body Mass Index of >30 kg/m^2^ or <18 kg/m^2^

# **Other**

## **Funding**

This study was investigator-initiated, and implemented by MD Jørgen Berg Dahl, and MD Morten Sejer Hansen.

Costs in connection with the study relate to the pay of the study participants as well expenses related to equipment. The participants will receive 150 DKK per hour (1 hour for screening and 2 study days with 1 hour per study day). The pay is taxable. External funding will be applied for in order to cover the expenses related to the study. The funds will be transferred to individual research accounts established specifically to the individual fund. The accounts are subjected to official audit. The study participants will be informed if any external funding is granted.

The investigator is employed at the Department of Anaesthesia 4231, HOC, Rigshospitalet, and has no financial interest in the trial.

## **Participant’s completion of and withdrawal from the trial**

- A study participant who has completed the study is defined as a study participant who has followed the trial's plan for the prescribed 2 study days.
- A study participant who has not completed the study is defined as a participant included in the trial, i.e. who has given informed consent, but did not complete the trial.
- If a study participant does not complete the trial an account should be given as to whether and how this participant is followed in the trial – this also applies to drop-outs – as well as what data has been collected from these participants.

## **Reasons for participant’s withdrawal from the trial**

A study participant can be withdrawn from the trial under the following conditions:

- If the investigator believes that a change of trial plan will be in the best interest of the study participant.
- If the participant wishes to withdraw from the trial.
- Violation of protocol rules.

## **Procedure for participants who withdraw from the trial**

In accordance with the Declaration of Helsinki, study participants have the right to withdraw from the trial at any time for any given reason. The investigator also has the right to withdraw a study participant from the trial at any time.

The reason why a study participant is withdrawn from the trial before the scheduled time shall be recorded in the study participant’s Case Report Form. If the participant will not describe the reason of withdrawal, it will also be recorded in the participants Case Report Form.

# **METHODOLOGY**

## **General procedure**

On the study days all study participants will be tested lying in the supine position. The study will be conducted in a quiet secluded room, where only the study participant and the investigator will be present.

The study participants will be tested with 3 types of pain conditioning on four separate study days. The 3 types of pain conditioning consist of Brief Thermal Sensitisation (BTS), Heat Pain Detection

Threshold (HPDT), and Long Thermal Stimulation (LTS), (see Clinical evaluations). HPDT and LTS are conducted subsequent to BTS. , the order of the stimulations (HPDT and LTS) is randomised for each patient and each experimental day, by a randomisation schedule developed by Copenhagen Trial Unit. Likewise, the investigators/observers responsible for testing and data registration will also be randomised, so the same investigator will not be responsible for 2 consecutive experimental days. There will be 2 different investigators responsible for the testing. The randomisation schedule is stored in sealed and opaque envelopes to secure adequate allocation concealment

The study participants will be tested with three types of pain conditioning on four separate experimental days. Furthermore, the study participants will complete the two psychological tests, PCS and HADS (see Assessments and tests)

The study participants will be tested with the following types of pain conditioning:

1. Brief Thermal Sensitisation (BTS). The test is conducted centrally on the anterior part of the right thigh. BTS will be conducted at the time: 0 min. (see Assessments and tests).
2. Heat pain detection threshold (HPDT). The test is conducted centrally on the anterior part of the dominant lower arm. HPDT (see Assessments and tests) will be conducted at the time: 7 or 10 min. depending on the randomisation (see Randomisation).
3. Long thermal stimulation (LTS). The test is conducted centrally on the anterior part of the non-dominant lower arm. A continuous VAS-measurement will be performed during the test. The test will be conducted at the time: 7 or 10 min. depending on the randomisation (see Randomisation).

The entire study consists of one screening day and four identical study days. The screening and study days will proceed as follows:

Screening day:

- Oral information regarding the project is delivered by the primary investigator. The participant will be given time to consider the information.
- Short medical history by the primary investigator. Thorough screening of in- and exclusion criteria. Measurement of height, weight, blood pressure and pulse.
- In order to familiarise the study participant with the types of pain conditioning, the 3 types of pain conditioning test are presented and performed on the study participant.
- Distribution of PCS and HADS (in Danish), together with an opaque envelope, in which the answered test are to be put, and a sealed envelope will be returned to the primary investigator on the first study day.
- Potentially clarifying questions concerning the study or PCS and HADS are answered.

Study day 1, 2, 3, and 4:

- Inspection of signed informed consent and written authority.
- Returning of the filled in PCS and HADS questionnaires in a sealed opaque envelope to ensure that the investigators cannot access the data prematurely (study day 1). The envelope is opened when the study participant have completed the four experimental days.
- The study participant is placed on a bed in a supine position.
- Commencement of study:
  - 0 min: Brief thermal sensitisation.
  - 7 min: HPDT or LTS (depending on the randomisation)
  - 10 min: HPDT or LTS (depending on the randomisation)

# **Clinical evaluations**

**Pain assessment**

Visual analogue scale (VAS), index from 0-100 mm, where 0 mm represents “no pain”, and 100 mm represents “the worst pain imaginable”.

**Brief Thermal Sensitization (BTS)**

A computer-controlled thermode (Somedic MSA Thermotester^TM^; size 2.5x5 cm) is placed on the participant’s skin, and the skin is heated to 45°C for 3 min. After 3 min., while the thermode still has contact with the skin, the assessment of secondary hyperalgesia is conducted.

**Assessment of secondary hyperalgesia**

The area of secondary hyperalgesia is quantified after stimulation with a 19G monofilament (von Frey hair) in 4 linear paths arranged in 90° around the centre of stimulation. Stimulation will begin 15 cm. from the centre of stimulation and advance in steps of 5 mm. with 1 second intervals towards the centre of stimulation. When the participant states a clear change in sensation the place will be marked with a felt pen and the transverse and longitudinal axes will be measured for later area calculation.

**Long Thermal Stimulation (LTS)**

The computer-controlled thermode is placed on the participant’s skin, and the skin is heated to 45°C for 1 min. During this the participant evaluates the pain with an electronic VAS-scale. The participant will continuously evaluate the pain using the electronic VAS-scale because of the fluctuations in pain intensity during this type of stimulation. The equipment automatically calculates a VAS-score under the curve (VAS-AUC) and a maximum VAS score for the time period. The participant will not be able to see the computer screen during the measurement, and each pain evaluation will be independent of the previous evaluation.

**Heat Pain Detection Threshold (HPDT)**

Heat pain detection threshold represents the lowest temperature that is perceived as painful, when heating the skin with the computer-controlled thermode. The initial temperature is 32°C, and temperature is increased 1°C/sec. The participant is asked to press a button when the heat is perceived as painful. If 52°C (the maximum temperature for the thermode) is reached before the participant’s threshold has been registered, the thermode will automatically return to the initial temperature of 32°C. The HPDT is calculated as an average of four stimulations. Each stimulation will be performed with an interval of 6-10 seconds.

**Hospital Anxiety and Depression scale (HADS)**

HADS is a questionnaire consisting of 14 questions, and is a 4-point Likert scale with values from 0-3. HADS can be subdivided into HADS-A, evaluating anxiety, and HADS-D, evaluating depression. The highest achievable score is 42, and a total HADS-score will estimate the participant’s level of distress. To evaluate anxiety and depression separately, HADS-A and HADS-D must be evaluated separately, with a maximum score of 21 in the two subtests (20).

The interpretation of the score in HADS-A and HADS-D is as follows:

- 0-7: Normal
- 8: Mild level of anxiety/depression
- 11-15: Moderate level of anxiety/depression
- >16: Severe level of anxiety/depression

The HADS questionnaire is to be completed before study day 1.

**Pain Catastrophizing Scale (PCS)**

PCS is a questionnaire consisting of 13 questions. PCS is a 5-point Likert scale with values from 0-4, and can be subdivided into 3 subtests, that each evaluates the central elements in catastrophizing: Rumination, magnification and helplessness. The highest achievable score is 52, and with separate evaluation of the three subtests, the 3 different elements can be assessed individually. To evaluate the 3 elements separately the 13 questions must be evaluated in the 3 following subgroups:

- Rumination: The sum of question 8, 9, 10, 11. Maximal sum =16
- Magnification: The sum of question 6, 7, 13. Maximal sum = 12
- Helplessness: The sum of question 1, 2, 3, 4, 5, 12. Maximal sum = 24

The PCS questionnaire is to be completed before study day 1.

# **ADVERSE EFFECTS**

## **Adverse effects/adverse events (AEs)**

Any adverse event, signs or symptoms that occur during participation in the study, which are time related to study procedure, whether the adverse event is considered to be related to the study procedure or not will be registered in the study participant’s Case Report Forms. If an adverse event occurs more than 48 hours after the last procedure of the trial, and there is no apparent causal connection or relation to the study procedure, this shall not be deemed to be an adverse event.

Start and end date/time, severity and effects after the study procedure must be recorded for all incidents. The severity of the adverse event and the relationship with the study procedure must be assessed in accordance with the guidelines described below.

## **Guidelines for adverse events' possible relationship to treatment:**

1. Not related – no temporal relationship, other aetiologies are very likely the cause
2. Possibly related – less clear correlation, other aetiologies are also possible
3. Probably related – clear temporal correlation with improvement on discontinuation of medication, and not reasonably explained by the patient's known clinical condition.
4. Related – clear temporal relationship with repeated treatment test or clinical assessment.

Study participants with adverse events will be monitored with appropriate clinical assessments and laboratory tests according to the decision of the attending doctor. All adverse events will be followed until satisfactory recovery or stabilization.

A serious adverse event (SAE) is understood to be any incident involving a significant risk of the death or disability of the study participants (or their offspring), including, but not limited to, an event that results in:

- death
- life-threatening episodes – in the investigator's opinion the study participant was in immediate risk of death from the adverse event when it appeared
- requires hospitalization or prolongs existing hospitalization
- is permanently disabling

- is a congenital anomaly

## **Grading of adverse events:**

The medical investigator should attempt to identify all clinical and objective reactions from the study participants in the treatment and determine their relationship with the study procedure. Reactions, if there are any, should be graded according to the following scale:

1 = slight

2 = moderate

3 = severe

4 = life-threatening

## **Reporting of adverse (AE) and serious adverse effects of (SAEs)**

The investigator is responsible for ensuring that all adverse events are recorded in the participant’s Case Report Form.

The sponsor is responsible for ongoing monitoring of the study's risk/benefit relation. If there arise or are seen to be situations that may affect the safety of the study participants or the performance of the study, these must **always** be immediately reported to all investigators involved with the study, as well as the relevant Research Ethic Comities.

The sponsor shall at all-time be kept informed by the investigator of any adverse events. At the end of the trial, the final report shall contain a description of all side effects.

Serious adverse events – SAEs - should be reported immediately by the investigator to the sponsor (sponsor-investigator). SAEs must be reported annually by the Investigator to the Research Ethics Committee throughout the whole trial period, together with a report on the safety of the study participants.

# **Statistical analyses**

## **Sample size estimation**

The estimation of the number participants, experimental days and number of observers will be based on simulations performed with selected relevant scenarios for the desired precision of the involved variance components. The simulations will be based on raw data obtained from a previous study (2). Even though the ratio between the intra-individual variance and the inter-individual variance in secondary hyperalgesia may exceed 25% we aim to include a slightly higher number of participants and implement a larger number of study days in order to investigate the reproducibility the area of secondary hyperalgesia.

The simulations will include scenarios with up to 10% missing data in a missing at random (MAR) scenario, where the risk of missingness is increased on the second experimental day in study participants who have large areas of secondary hyperalgesia on the first experimental day.

## **Plan of statistical analysis**

To determine the variance of the areas of secondary hyperalgesia derived respectively from the study participant, the experimental day and the observer, a variance component model will be employed, to investigate which of these three types of variance is the most influential of the total variance.

’Limits of agreements’ plot for agreement between areas of secondary hyperalgesia on different experimental days and different observers will be developed and evaluated in accordance with the relevant measures of reproducibility. For each of the secondary outcomes, the ability of PCS, HADS, HPDT, Max-VAS and VAS-AUC (following LTS), to predict individual variations in areas of secondary hyperalgesia will be investigated by linear regression. Significance of the predictors will be assessed by Analysis of variance (ANOVA) methods and their predictive abilities will be quantified by various summaries of prediction errors including 95% prediction intervals for the predictions

For all analyses, an intention-to-test analysis will be performed including all subjects who participated in the first experimental day. Analyses will be based on all observed data. If missingness exceeds 5% and there is indication of violation of “missing completely at random” (MCAR) a sensitivity analysis based on an appropriate model for missingness at random (MAR) or not at random (MNAR) will be performed.

A family wise error rate of 0.05 will be used and simultaneous assessment of multiple p-values will be subjected to single-step adjustment

#

# **DATA REGISTRATION AND RULES FOR THE CONTROL OF INVESTIGATION PROCEDURES**

The study will be conducted in accordance with the applicable rules on clinical trials involving people in respect of quality control and quality management.

The investigator at the hospital is responsible for managing and archiving data in accordance with current regulations. The data belongs to the sponsor and investigators at Rigshospitalet, Department of Anaesthesia 4231, HOC.

The study will be submitted to the Danish Data Protection Agency, the Danish Research Ethics Committee, and will be registered at the website: www.clinicaltrials.gov.

## **Case Report Forms**

A Case Report Form (CRF) will be completed for each volunteer included in the study. It will be signed by the investigator to confirm the accuracy of the data.

Source data will consist of the following data:

- Age (year)
- Height, weight, and Body Mass Index (BMI)
- Blood pressure and pulse
- Pain catastrophizing scale (PCS), completed
- Hospital pain and anxiety scale (HADS), completed
- For study 1-4:
- Test results
  - Result of Heat Pain Detection Threshold
  - Result of Long Thermal Stimulation
  - Result of Brief Thermal Sensitisation
  - Calculated area of secondary hyperalgesia, following Brief Thermal Sensitisation
- Check boxes indicating following procedures are executed:
  - BTS executed, LTS executed, HPDT executed, completed PCS received, completed HADS received.
- Field to fill in possible side effects and/or complications.

## **Training**

The investigator will ensure that the personnel involved are appropriately trained and qualified, and have the necessary information to carry out the study.

# **ADDITIONAL REQUIREMENTS AND GENERAL INFORMATION**

# **Insurance**

For healthy volunteers at Rigshospitalet, the Anesthesia and Surgical Clinic, Centre of Head and Orthopaedics, Rigshospitalet, accepts statutory responsibility on behalf of the investigator and the investigator's colleagues for any injury caused directly or indirectly by the study procedure in this clinical study, provided that the investigator and the investigator's colleagues have followed the instructions given in this protocol and any supplements thereto, and that the investigator and the investigator's colleagues conducted the study scientifically and in accordance with applicable rules and accepted techniques. In the event of injury or death unrelated to the conduct of the study, participants are covered by the hospital's insurance.

## **Publication of results**

On the basis of the data the investigator will write a report of the study. This report will be forwarded to the relevant authorities. The report will also form the basis of a manuscript to be submitted for publication with the following order of authors:

- - - 1. **Morten Sejer Hansen**
      2. **Rebecca Østervig**
      3. **Jørn Wetterslev**
      4. **Christian Bressen Piper**
      5. **Mohammed Sohail Asghar**
      6. **Emilia Horjales-Araujo**
      7. **Karen Lisa Hilsted**
      8. **Jørgen Berg Dahl**

Negative, positive, conclusive, and inconclusive test results will be published.

# **References**

1. Woolf CJ. Central sensitization: implications for the diagnosis and treatment of pain. Pain. 2011;152(3 Suppl):S2-15. doi: 0.1016/j.pain.2010.09.030. Epub Oct 18.

2. Dirks J, Petersen KL, Dahl JB. The heat/capsaicin sensitization model: a methodologic study. J Pain. 2003;4(3):122-8.

3. Mathiesen O, Imbimbo BP, Hilsted KL, Fabbri L, Dahl JB. CHF3381, a N-methyl-D-aspartate receptor antagonist and monoamine oxidase-A inhibitor, attenuates secondary hyperalgesia in a human pain model. J Pain. 2006;7(8):565-74.

4. Werner MU, Petersen KL, Rowbotham MC, Dahl JB. Healthy volunteers can be phenotyped using cutaneous sensitization pain models. PLoS One. 2013;8(5):e62733. doi: 10.1371/journal.pone.0062733. Print 2013.

5. Pereira MP, Werner MU, Ringsted TK, Rowbotham MC, Taylor BK, Dahl JB. Does naloxone reinstate secondary hyperalgesia in humans after resolution of a burn injury? A placebo-controlled, double-blind, randomized, cross-over study. PLoS One. 2013;8(5):e64608. doi: 10.1371/journal.pone.0064608. Print 2013.

6. Norbury TA, MacGregor AJ, Urwin J, Spector TD, McMahon SB. Heritability of responses to painful stimuli in women: a classical twin study. Brain. 2007;130(Pt 11):3041-9. Epub 2007 Oct 11.

7. Tegeder I, Adolph J, Schmidt H, Woolf CJ, Geisslinger G, Lotsch J. Reduced hyperalgesia in homozygous carriers of a GTP cyclohydrolase 1 haplotype. Eur J Pain. 2008;12(8):1069-77. doi: 10.16/j.ejpain.2008.02.004. Epub Apr 18.

8. Williams FM, Scollen S, Cao D, Memari Y, Hyde CL, Zhang B, et al. Genes contributing to pain sensitivity in the normal population: an exome sequencing study. PLoS Genet. 2012;8(12):e1003095. doi: 10.1371/journal.pgen.. Epub 2012 Dec 20.

9. Latremoliere A, Woolf CJ. Central sensitization: a generator of pain hypersensitivity by central neural plasticity. J Pain. 2009;10(9):895-926. doi: 10.1016/j.jpain.2009.06.012.

10. Naert AL, Kehlet H, Kupers R. Characterization of a novel model of tonic heat pain stimulation in healthy volunteers. Pain. 2008;138(1):163-71. doi: 10.1016/j.pain.2007.11.018. Epub 8 Jan 22.

11. Petersen KL, Rowbotham MC. A new human experimental pain model: the heat/capsaicin sensitization model. Neuroreport. 1999;10(7):1511-6.

12. Staahl C, Olesen AE, Andresen T, Arendt-Nielsen L, Drewes AM. Assessing efficacy of non-opioid analgesics in experimental pain models in healthy volunteers: an updated review. Br J Clin Pharmacol. 2009;68(3):322-41. doi: 10.1111/j.365-2125.009.03433.x.

13. Lee JE, Watson D, Frey-Law LA. Psychological factors predict local and referred experimental muscle pain: a cluster analysis in healthy adults. Eur J Pain. 2013;17(6):903-15. doi: 10.1002/j.532-2149.012.00249.x. Epub 2012 Nov 19.

14. Sullivan MJ, Thorn B, Haythornthwaite JA, Keefe F, Martin M, Bradley LA, et al. Theoretical perspectives on the relation between catastrophizing and pain. Clin J Pain. 2001;17(1):52-64.

15. Papaioannou M, Skapinakis P, Damigos D, Mavreas V, Broumas G, Palgimesi A. The role of catastrophizing in the prediction of postoperative pain. Pain Med. 2009;10(8):1452-9. doi: 10.111/j.526-4637.2009.00730.x. Epub 2009 Oct 26.

16. Zigmond AS, Snaith RP. The hospital anxiety and depression scale. Acta Psychiatr Scand. 1983;67(6):361-70.

17. Sullivan MJL, Bishop SR, Pivik J. The Pain Catastrophizing Scale: Development and validation. Psychol Assess. 1995;7(4):524-32.

18. Bjelland I, Dahl AA, Haug TT, Neckelmann D. The validity of the Hospital Anxiety and Depression Scale. An updated literature review. J Psychosom Res. 2002;52(2):69-77.

19. Van Damme S, Crombez G, Bijttebier P, Goubert L, Van Houdenhove B. A confirmatory factor analysis of the Pain Catastrophizing Scale: invariant factor structure across clinical and non-clinical populations. Pain. 2002;96(3):319-24.

20. Smarr KL, Keefer AL. Measures of depression and depressive symptoms: Beck Depression Inventory-II (BDI-II), Center for Epidemiologic Studies Depression Scale (CES-D), Geriatric Depression Scale (GDS), Hospital Anxiety and Depression Scale (HADS), and Patient Health Questionnaire-9 (PHQ-9). Arthritis Care Res (Hoboken). 2011;63(Suppl 11):S454-66. doi: 10.1002/acr.20556.
